# Smoking prevalence and attributable disease burden in 195 countries and territories, 1990–2015: a systematic analysis from the Global Burden of Disease Study 2015

**DOI:** 10.1016/S0140-6736(17)30819-X

**Published:** 2017-05-13

**Authors:** GBD 2015 Tobacco Collaborators, Marissa B Reitsma, Nancy Fullman, Marie Ng, Joseph S Salama, Amanuel Abajobir, Kalkidan Hassen Abate, Cristiana Abbafati, Semaw Ferede Abera, Biju Abraham, Gebre Yitayih Abyu, Akindele Olupelumi Adebiyi, Ziyad Al-Aly, Alicia V Aleman, Raghib Ali, Ala'a Al Alkerwi, Peter Allebeck, Rajaa Mohammad Al-Raddadi, Azmeraw T Amare, Alemayehu Amberbir, Walid Ammar, Stephen Marc Amrock, Carl Abelardo T Antonio, Hamid Asayesh, Niguse Tadela Atnafu, Peter Azzopardi, Amitava Banerjee, Aleksandra Barac, Tonatiuh Barrientos-Gutierrez, Ana Cristina Basto-Abreu, Shahrzad Bazargan-Hejazi, Neeraj Bedi, Brent Bell, Aminu K Bello, Isabela M Bensenor, Addisu Shunu Beyene, Neeraj Bhala, Stan Biryukov, Kaylin Bolt, Hermann Brenner, Zahid Butt, Fiorella Cavalleri, Kelly Cercy, Honglei Chen, Devasahayam Jesudas Christopher, Liliana G Ciobanu, Valentina Colistro, Mercedes Colomar, Leslie Cornaby, Xiaochen Dai, Solomon Abrha Damtew, Lalit Dandona, Rakhi Dandona, Emily Dansereau, Kairat Davletov, Anand Dayama, Tizta Tilahun Degfie, Amare Deribew, Samath D Dharmaratne, Balem Demtsu Dimtsu, Kerrie E Doyle, Aman Yesuf Endries, Sergey Petrovich Ermakov, Kara Estep, Emerito Jose Aquino Faraon, Farshad Farzadfar, Valery L Feigin, Andrea B Feigl, Florian Fischer, Joseph Friedman, Tsegaye Tewelde G/hiwot, Seana L Gall, Wayne Gao, Richard F Gillum, Audra L Gold, Sameer Vali Gopalani, Carolyn C Gotay, Rahul Gupta, Rajeev Gupta, Vipin Gupta, Randah Ribhi Hamadeh, Graeme Hankey, Hilda L Harb, Simon I Hay, Masako Horino, Nobuyuki Horita, H Dean Hosgood, Abdullatif Husseini, Bogdan Vasile Ileanu, Farhad Islami, Guohong Jiang, Ying Jiang, Jost B Jonas, Zubair Kabir, Ritul Kamal, Amir Kasaeian, Chandrasekharan Nair Kesavachandran, Yousef S Khader, Ibrahim Khalil, Young-Ho Khang, Sahil Khera, Jagdish Khubchandani, Daniel Kim, Yun Jin Kim, Ruth W Kimokoti, Yohannes Kinfu, Luke D Knibbs, Yoshihiro Kokubo, Dhaval Kolte, Jacek Kopec, Soewarta Kosen, Georgios A Kotsakis, Parvaiz A Koul, Ai Koyanagi, Kristopher J Krohn, Hans Krueger, Barthelemy Kuate Defo, Burcu Kucuk Bicer, Chanda Kulkarni, G Anil Kumar, Janet L Leasher, Alexander Lee, Mall Leinsalu, Tong Li, Shai Linn, Patrick Liu, Shiwei Liu, Loon-Tzian Lo, Alan D Lopez, Stefan Ma, Hassan Magdy Abd El Razek, Azeem Majeed, Reza Malekzadeh, Deborah Carvalho Malta, Wondimu Ayele Manamo, Jose Martinez-Raga, Alemayehu Berhane Mekonnen, Walter Mendoza, Ted R Miller, Karzan Abdulmuhsin Mohammad, Lidia Morawska, Kamarul Imran Musa, Gabriele Nagel, Sudan Prasad Neupane, Quyen Nguyen, Grant Nguyen, In-Hwan Oh, Abayomi Samuel Oyekale, Mahesh PA, Adrian Pana, Eun-Kee Park, Snehal T Patil, George C Patton, Joao Pedro, Mostafa Qorbani, Anwar Rafay, Mahfuzar Rahman, Rajesh Kumar Rai, Usha Ram, Chhabi Lal Ranabhat, Amany H Refaat, Nickolas Reinig, Hirbo Shore Roba, Alina Rodriguez, Yesenia Roman, Gregory Roth, Ambuj Roy, Rajesh Sagar, Joshua Salomon, Juan Sanabria, Itamar de Souza Santos, Benn Sartorius, Maheswar Satpathy, Monika Sawhney, Susan Sawyer, Mete Saylan, Michael P Schaub, Neil Schluger, Aletta Elisabeth Schutte, Sadaf G Sepanlou, Berrin Serdar, Masood Ali Shaikh, Jun She, Min-Jeong Shin, Rahman Shiri, Kawkab Shishani, Ivy Shiue, Inga Dora Sigfusdottir, Jonathan I Silverberg, Jasvinder Singh, Virendra Singh, Erica Leigh Slepak, Samir Soneji, Joan B Soriano, Sergey Soshnikov, Chandrashekhar T Sreeramareddy, Dan J Stein, Saverio Stranges, Michelle L Subart, Soumya Swaminathan, Cassandra E I Szoeke, Worku Mekonnen Tefera, Roman Topor-Madry, Bach Tran, Nikolaos Tsilimparis, Hayley Tymeson, Kingsley Nnanna Ukwaja, Rachel Updike, Olalekan A Uthman, Francesco Saverio Violante, Sergey K Vladimirov, Vasiliy Vlassov, Stein Emil Vollset, Theo Vos, Elisabete Weiderpass, Chi-Pan Wen, Andrea Werdecker, Shelley Wilson, Mamo Wubshet, Lin Xiao, Bereket Yakob, Yuichiro Yano, Penpeng Ye, Naohiro Yonemoto, Seok-Jun Yoon, Mustafa Z Younis, Chuanhua Yu, Zoubida Zaidi, Maysaa El Sayed Zaki, Anthony Lin Zhang, Ben Zipkin, Christopher J L Murray, Mohammad H Forouzanfar, Emmanuela Gakidou

## Abstract

**Background:**

The scale-up of tobacco control, especially after the adoption of the Framework Convention for Tobacco Control, is a major public health success story. Nonetheless, smoking remains a leading risk for early death and disability worldwide, and therefore continues to require sustained political commitment. The Global Burden of Diseases, Injuries, and Risk Factors Study (GBD) offers a robust platform through which global, regional, and national progress toward achieving smoking-related targets can be assessed.

**Methods:**

We synthesised 2818 data sources with spatiotemporal Gaussian process regression and produced estimates of daily smoking prevalence by sex, age group, and year for 195 countries and territories from 1990 to 2015. We analysed 38 risk-outcome pairs to generate estimates of smoking-attributable mortality and disease burden, as measured by disability-adjusted life-years (DALYs). We then performed a cohort analysis of smoking prevalence by birth-year cohort to better understand temporal age patterns in smoking. We also did a decomposition analysis, in which we parsed out changes in all-cause smoking-attributable DALYs due to changes in population growth, population ageing, smoking prevalence, and risk-deleted DALY rates. Finally, we explored results by level of development using the Socio-demographic Index (SDI).

**Findings:**

Worldwide, the age-standardised prevalence of daily smoking was 25·0% (95% uncertainty interval [UI] 24·2–25·7) for men and 5·4% (5·1–5·7) for women, representing 28·4% (25·8–31·1) and 34·4% (29·4–38·6) reductions, respectively, since 1990. A greater percentage of countries and territories achieved significant annualised rates of decline in smoking prevalence from 1990 to 2005 than in between 2005 and 2015; however, only four countries had significant annualised increases in smoking prevalence between 2005 and 2015 (Congo [Brazzaville] and Azerbaijan for men and Kuwait and Timor-Leste for women). In 2015, 11·5% of global deaths (6·4 million [95% UI 5·7–7·0 million]) were attributable to smoking worldwide, of which 52·2% took place in four countries (China, India, the USA, and Russia). Smoking was ranked among the five leading risk factors by DALYs in 109 countries and territories in 2015, rising from 88 geographies in 1990. In terms of birth cohorts, male smoking prevalence followed similar age patterns across levels of SDI, whereas much more heterogeneity was found in age patterns for female smokers by level of development. While smoking prevalence and risk-deleted DALY rates mostly decreased by sex and SDI quintile, population growth, population ageing, or a combination of both, drove rises in overall smoking-attributable DALYs in low-SDI to middle-SDI geographies between 2005 and 2015.

**Interpretation:**

The pace of progress in reducing smoking prevalence has been heterogeneous across geographies, development status, and sex, and as highlighted by more recent trends, maintaining past rates of decline should not be taken for granted, especially in women and in low-SDI to middle-SDI countries. Beyond the effect of the tobacco industry and societal mores, a crucial challenge facing tobacco control initiatives is that demographic forces are poised to heighten smoking's global toll, unless progress in preventing initiation and promoting cessation can be substantially accelerated. Greater success in tobacco control is possible but requires effective, comprehensive, and adequately implemented and enforced policies, which might in turn require global and national levels of political commitment beyond what has been achieved during the past 25 years.

**Funding:**

Bill & Melinda Gates Foundation and Bloomberg Philanthropies.

## Introduction

Smoking was the second leading risk factor for early death and disability worldwide in 2015.^1^ It has claimed more than 5 million lives every year since 1990,^1^ and its contribution to overall disease burden is growing, especially in lower income countries. The negative effects of smoking extend well beyond individual and population health^2^ as billions of dollars in lost productivity and health-care expenditure are related to smoking every year.^3^ Successfully combatting the tobacco industry's pursuit of new smokers has been further complicated by the substantive—and sometimes rapid—social, demographic, and economic shifts occurring worldwide.^4^, ^5^, ^6^ As the tobacco industry moves to target previously untapped markets,^6^, ^7^, ^8^ strong tobacco control policies and timely monitoring of smoking patterns are imperative.

**Research in context**

**Evidence before this study**

Smoking is a widely recognised risk factor for premature morbidity and mortality, but adequate monitoring of smoking levels and trends throughout the world has been challenging. Increasing investments in multi-country survey series has improved the availability of data for smoking behaviours, especially in lower income countries, but such surveys are quite infrequent and differences in survey questions and definitions can hinder appropriate comparisons between countries and across time. Through the Global Burden of Diseases, Injuries, and Risk Factors 2013 Study (GBD 2013), researchers collated diverse data sources and synthesised them to produce comprehensive, comparable estimates of daily smoking prevalence, by sex and age group, for 188 countries from 1990 to 2013. Additional analyses, including those by Bilano and colleagues in 2015, have applied similar methods to project trends in tobacco use through 2025 in 173 countries for men and 178 countries for women.

**Added value of this study**

With the 2015 update to the GBD, the number of data sources included was substantially increased and the estimation process for both smoking prevalence and attributable disease burden, as measured by disability-adjusted life-years (DALYs), has been improved. Two novel analyses are also provided through the GBD 2015 study: a birth cohort analysis of smoking patterns over time and a decomposition analysis to parse out changes in total DALYs attributable to smoking to changes in population growth, population ageing, smoking prevalence, and risk-deleted DALY rates. The latter assessment can assist with identifying what factors are contributing to changes in disease burden due to smoking–demographic trends, efforts to address smoking, or some combination of these factors. Further, we used the Socio-demographic Index (SDI), a new summary measure of overall development from GBD 2015, to assess levels and trends in smoking prevalence and attributable burden across the development spectrum.

**Implications of all the available evidence**

Amid gains in tobacco control worldwide, smoking remains a leading risk factor for early death and disability. Although there have been some success stories, for many countries and territories, faster annualised rates of decline in smoking prevalence occurred between 1990 and 2005 than between 2005 and 2015. Although smoking prevalence and risk-deleted DALY rates fell across SDI quintiles, population growth and ageing ultimately offset these gains and contributed to overall increases in smoking-attributable disease burden in low to middle SDI geographies. Intensified tobacco control and strengthened monitoring are required to further reduce smoking prevalence and attributable burden, especially in view of the fact that demographic factors like population ageing are not easily amenable to intervention.

The past decade has brought a substantial expansion and strengthening of tobacco control initiatives, harnessing a wide range of effective interventions and policy instruments for addressing the tobacco epidemic.^9^, ^10^, ^11^, ^12^, ^13^, ^14^, ^15^, ^16^ Successful strategies include taxation of tobacco products,^9^ bans on smoking in public places and instituting smoke-free zones,^10^, ^11^ restrictions on the marketing and promotion of cigarettes, including plain packaging laws,^12^ community-wide and nation-wide smoking cessation interventions,^13^, ^14^ and enforcement of both text and pictorial warning labels on tobacco products.^15^, ^16^ Efforts to implement comprehensive tobacco control policies culminated in the adoption of the WHO Framework Convention on Tobacco Control (FCTC) in 2003.^17^ The FCTC, the world's first public health treaty, is viewed as a key driver of recent progress in reducing tobacco consumption and smoking prevalence in many regions of the world.^18^ As of 2016, 180 parties have ratified the FCTC,^19^ and many use WHO's MPOWER measures,^20^ established in 2008, to guide national and local FCTC compliance.^21^ More recently, WHO introduced the 25×25 non-communicable disease (NCD) targets, which include decreasing tobacco use by 30% between 2010 and 2025.^22^ Several countries have committed to an even stronger anti-smoking goal, setting national targets to become tobacco-free.^23^ Additionally, strengthening FCTC implementation was explicitly included in the United Nations' Sustainable Development Goals (SDGs).^24^ With tobacco control's increasing prioritisation on the global stage, accurately monitoring patterns in smoking and associated health outcomes is critical for identifying optimal intervention strategies across geographies, demographic groups, and the development spectrum.

Previous analyses of smoking prevalence and attributable disease burden often were hindered by poor data availability, methodological limitations, or both.^25^, ^26^, ^27^ Investments in survey series focused on tobacco, such as the Global Adult Tobacco Surveys (GATS) and the Global Youth Tobacco Surveys (GYTS), have supported more in-depth assessments of national tobacco use.^28^ Nonetheless, remaining data gaps across countries and time, as well as differences in smoking-related questions and definitions among available data sources, necessitated large analytical improvements to produce a systematic and consistent understanding of smoking patterns. As part of the Global Burden of Diseases, Injuries, and Risk Factors 2013 Study (GBD 2013), Ng and colleagues generated the first comprehensive, comparable estimates of smoking prevalence and tobacco consumption for 188 countries from 1980 to 2013.^29^ Since then, other studies have used similar data synthesis approaches to project smoking trends from 2010 to 2025 in 173 countries for men and 178 countries for women.^30^ Previous GBD studies^31^, ^32^ have assessed the contribution of smoking to overall disease burden through the comparative risk assessment framework developed by Murray and Lopez.^33^ Recent studies have quantified the global effects of tobacco on achieving NCD mortality targets^34^ and life expectancy,^35^ while several assessed smoking-attributable mortality and non-fatal health outcomes for specific locations.^36^, ^37^

In this analysis, we assess smoking prevalence and smoking-attributable disease burden, based on deaths and disability-adjusted life-years (DALYs), by sex and age group for 195 countries and territories from 1990 to 2015. We also investigate differences in smoking prevalence and attributable burden according to the Socio-demographic Index (SDI), a summary measure of income per capita, educational attainment, and total fertility rate.^38^ Additionally, we assess age and sex patterns by birth cohort across levels of development. Finally, we perform a decomposition analysis of potential drivers of smoking-attributable disease burden over time.

## Methods

This study follows the overall GBD 2015 comparative risk assessment framework, details of which have been previously published.^1^ Here we summarise the main steps in the estimation process; the appendix provides more details about data inputs and modelling strategies (pp 5–9). This study fully adheres to the Guidelines for Accurate and Transparent Health Estimates Reporting (GATHER).^39^

### Estimating smoking exposure

Improving upon methods used by Ng and colleagues,^29^ we calculated two exposure measures: prevalence of daily smoking of tobacco and the smoking impact ratio. We defined a daily smoker as an individual using any type of smoked tobacco product on a daily basis.^40^, ^41^ We used 2818 data sources, covering 2928 geography-years of data, identified through the Global Health Data Exchange (GHDx), WHO InfoBase Database, and International Smoking Statistics Database; the appendix provides additional details on data sources (pp 5, 6). For any data that did not match our exposure definition we adjusted for frequency of use or type of tobacco consumed to avoid potential biases. We adjusted for smoking frequency and type simultaneously, which allowed us to account for their mutual correlations with each other (appendix pp 7, 8). Second-hand smoke exposure is estimated separately in GBD and is not included in this analysis.

We generated estimates of smoking prevalence by sex and 5-year age groups starting at age 10 years. Any data that spanned multiple age groups or were reported for both sexes combined were split based on the age-sex patterns recorded from data sources with multiple age-sex groupings.^29^ We then used spatiotemporal Gaussian process regression (ST-GPR), a data synthesis method widely used in GBD,^1^ which allowed us to draw strength across geography, time, and age, incorporate both data and model uncertainty, and produce a full time-series of estimates for all 195 geographies. The appendix provides full details on the modelling strategy (pp 5–9).

The second exposure measure, the smoking impact ratio, was first described by Peto and Lopez^42^ as part of a method to estimate smoking-attributable burden in the absence of information about smoking patterns. The smoking impact ratio is defined as the population lung cancer mortality rate in excess of the background lung cancer mortality rate recorded in non-smokers in the population, relative to the excess lung cancer mortality rate recorded in a reference group of smokers. We computed the smoking impact ratio for each analytic unit using the geography-specific, year-specific, age-specific, and sex-specific population lung cancer mortality rates from GBD 2015,^20^ and reference group lung cancer mortality rates from prospective cohort studies (appendix p 9).

### Defining risk-outcome pairs

We assessed all available evidence that supported causal associations between smoking and 38 health outcomes using a systematic approach adapted from Hill's criteria for causation^43^ and the World Cancer Research Fund evidence grading schema (appendix p 9).^44^ We added seven new outcomes to those used in GBD 2013:^31^ larynx cancer, peptic ulcer disease, rheumatoid arthritis, cataract, macular degeneration, hip fracture, and non-hip fracture.

### Estimating attributable burden

We used 5-year lagged smoking prevalence in estimating smoking attributable burden for cardiovascular diseases, tuberculosis, diabetes, lower respiratory infections, asthma, cataracts, macular degeneration, fractures, rheumatoid arthritis, and peptic ulcer disease. We chose a 5-year lag based on findings showing that most risk-reduction occurs within 5 years of quitting smoking.^45^ We used the smoking impact ratio in estimating smoking- attributable burden for cancers, chronic obstructive pulmonary disease (COPD), interstitial lung disease, other chronic respiratory diseases, and pneumoconiosis. The appendix provides a complete list of outcomes and their associated exposure metric (pp 31, 32).

For each outcome included in this analysis we used relative risk estimates derived from prospective cohort studies comparing smokers to never smokers (appendix p 9). Population attributable fractions were calculated based on estimates of exposure, relative risks, and the theoretical minimum risk exposure level for smoking (zero smoking). Following population attributable fraction calculation, we multiplied estimates of deaths and DALYs by outcome-specific population attributable fractions, and then summed them across all 38 outcomes to compute overall disease burden attributable to smoking (appendix p 9).

### Uncertainty analysis

We captured and propagated uncertainty through all steps of the analysis, including sampling uncertainty from data extraction, uncertainty from models used to adjust data reported in non-standard frequency-type combinations, uncertainty in the ST-GPR model, and uncertainty in deaths and DALYs for the 38 included outcomes. Ultimately, we produced 1000 draws of exposure and attributable burden estimates, for each geography, year, age, and sex, from which 95% uncertainty intervals (UIs) were taken using the 2·5 percentile and 97·5 percentile of the distribution.

### Decomposing changes in DALYs

To parse out the drivers of changes in smoking-attributable DALYs from 2005 to 2015, we assessed the relative contribution of four factors: population growth, population age structure, risk-deleted DALY rates, and smoking exposure. Risk-deleted rates are defined as the DALY rates that would have been recorded had we removed smoking as a risk factor. We estimated risk-deleted DALY rates by multiplying the observed cause-specific DALY rates by one minus the cause-specific population attributable fractions. For the decomposition analysis, we used the methods developed by Das Gupta (appendix p 10).^46^

### Smoking and its association with SDI

We present results aggregated by level of SDI, a composite indicator of development estimated for each geography based on lag-distributed income per capita, average educational attainment among individuals over age 15 years, and total fertility rate. SDI values were scaled to a range from 0 to 1.^38^ The appendix provides SDI values for each geography (pp 21–25).

### Role of the funding source

The funders of the study had no role in study design, data collection, data analysis, data interpretation, or writing of the report. The corresponding author had full access to all the data in the study and had final responsibility for the decision to submit for publication.

## Results

### Global, regional, and national levels and trends of daily smoking

**Figure 1:**
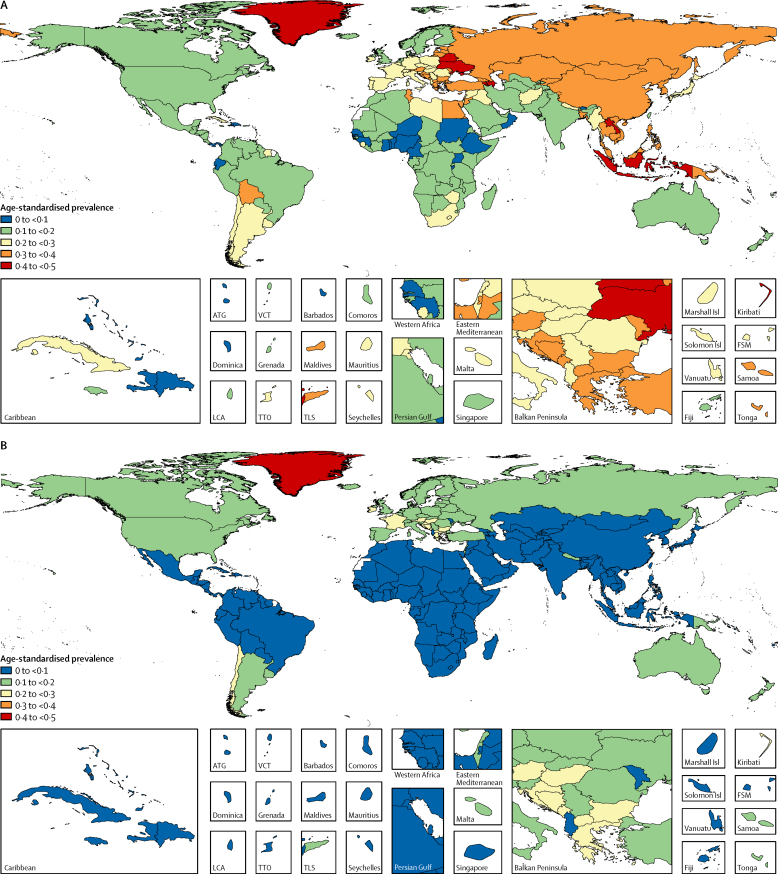
Age-standardised prevalence of daily smoking for men (A) and women (B), in 2015 ATG=Antigua and Barbuda. VCT=Saint Vincent and the Grenadines. LCA=Saint Lucia. TTO=Trinidad and Tobago. TLS=Timor-Leste. FSM=Federated States of Micronesia.

**Figure 2:**
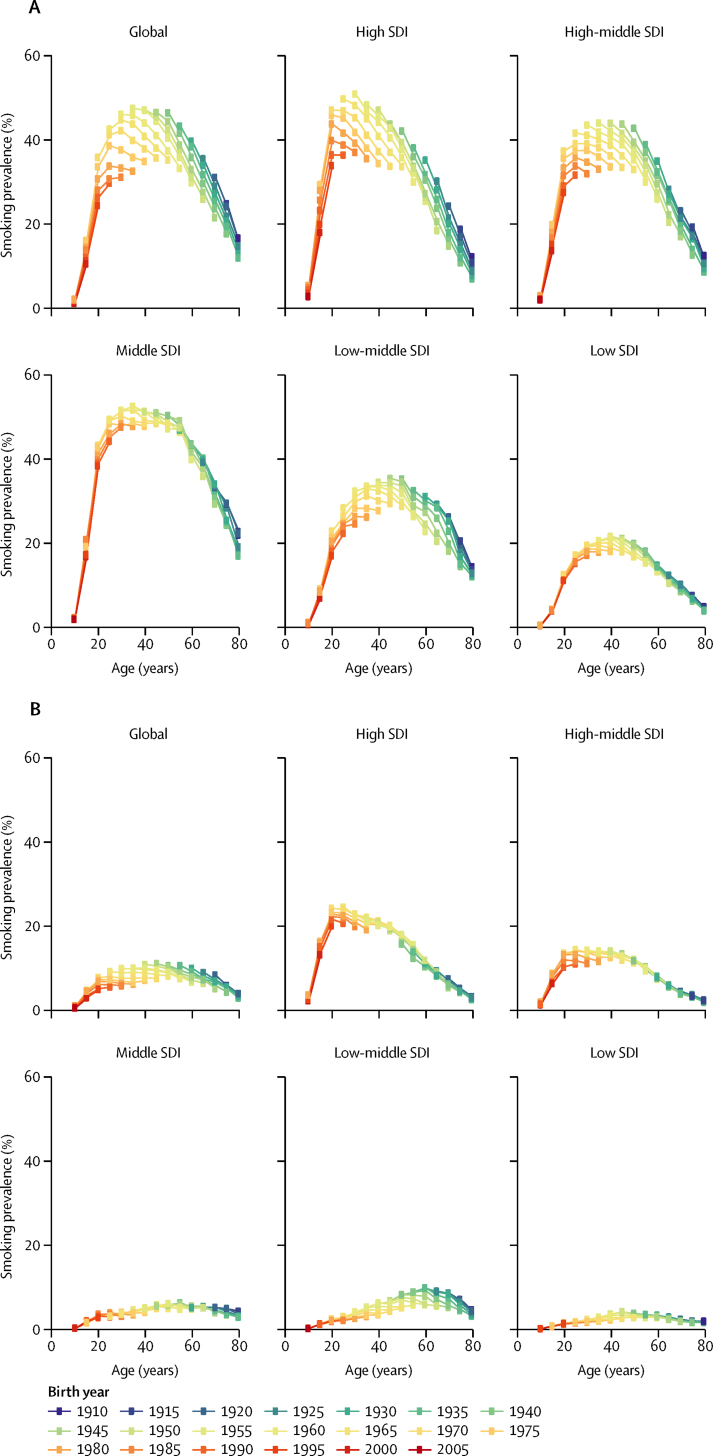
Prevalence of daily smoking across birth cohorts over time, at the global level and by SDI quintile, for men (A) and women (B) Birth cohorts are colour-coded by 5-year intervals, with the most recent birth cohort in red (2005) to the least recent birth cohort in dark blue (1910). Every dot represents the prevalence of daily smoking for a given birth cohort and age group. SDI=Socio-demographic Index.

Worldwide in 2015, the age-standardised prevalence of daily smoking was 25·0% (95% uncertainty interval [UI] 24·2–25·7) in men and 5·4% (5·1–5·7) in women (table 1). 51 countries and territories had significantly higher prevalence of smoking than the global average for men, and these countries were located mainly in central and eastern Europe and southeast Asia (figure 1). For women, 70 countries, mainly in western and central Europe, significantly exceeded the global average. Among men, prevalence of daily smoking was highest in middle SDI countries, whereas for women high SDI countries had the highest prevalence of daily smokers (figure 2). Compared with other SDI levels, low SDI geographies generally had the lowest prevalence of daily smoking for both sexes.

**Table 1:** Age-standardised prevalence of daily smoking in 2015 and annualised rate of change in age-standardised prevalence from 1990–2015, 1990–2005, and 2005–2015 for men and women

	**SDI level**	**2015 female age-standardised prevalence**	**2015 male age-standardised prevalence**	**Annualised rate of change, female 1990–2015**	**Annualised rate of change, male 1990–2015**	**Annualised rate of change, female 1990–2005**	**Annualised rate of change, male 1990–2005**	**Annualised rate of change, female 2005–2015**	**Annualised rate of change, male 2005–2015**
Global		5·4 (5·1 to 5·7)	25·0 (24·2 to 25·7)	−1·7 (−2·0 to −1·4)	−1·3 (−1·5 to −1·2)	−1·6 (−2·0 to −1·2)	−1·2 (−1·4 to −1·0)	−1·8 (−2·4 to −1·1)	−1·5 (−1·9 to −1·1)
Afghanistan	Low SDI	7·0 (4·6 to 9·7)	21·4 (18·4 to 24·7)	0·1 (−2·0 to 2·3)	0·5 (−0·4 to 1·3)	−0·1 (−3·5 to 3·1)	0·6 (−0·8 to 2·0)	0·4 (−4·2 to 4·6)	0·2 (−1·7 to 2·1)
Albania	High-middle SDI	2·3 (1·8 to 2·9)	29·0 (26·2 to 31·8)	0·0 (−1·3 to 1·2)	−0·5 (−1·0 to 0·0)	−0·8 (−2·6 to 1·1)	−1·0 (−1·7 to −0·3)	1·2 (−1·3 to 3·6)	0·2 (−0·8 to 1·2)
Algeria	Middle SDI	2·2 (1·5 to 3·2)	17·5 (14·9 to 20·4)	−5·3 (−7·4 to −3·3)	−1·2 (−2·1 to −0·3)	−5·4 (−8·6 to −2·5)	−1·7 (−3·0 to −0·4)	−5·1 (−9·6 to −0·7)	−0·4 (−2·1 to 1·5)
American Samoa	High-middle SDI	12·8 (10·3 to 15·4)	27·2 (23·7 to 31·1)	−0·1 (−1·3 to 1·0)	−0·4 (−1·2 to 0·3)	0·4 (−1·2 to 2·0)	−0·4 (−1·4 to 0·7)	−0·9 (−3·0 to 1·2)	−0·4 (−1·9 to 1·0)
Andorra	High SDI	18·4 (15·9 to 21·0)	24·9 (21·9 to 27·6)	−0·5 (−1·4 to 0·2)	−1·0 (−1·6 to −0·3)	−0·3 (−1·5 to 0·9)	−0·8 (−1·7 to 0·1)	−0·9 (−2·5 to 0·7)	−1·1 (−2·4 to 0·1)
Angola	Low-middle SDI	1·6 (0·9 to 2·6)	14·2 (12·5 to 16·1)	−0·7 (−3·5 to 2·2)	0·5 (−0·2 to 1·3)	−1·2 (−5·7 to 3·3)	0·4 (−0·8 to 1·5)	0·0 (−6·0 to 5·7)	0·8 (−0·8 to 2·3)
Antigua and Barbuda	High SDI	2·2 (1·6 to 3·0)	4·4 (3·4 to 5·7)	1·4 (−0·4 to 3·3)	0·6 (−0·9 to 2·0)	1·8 (−1·1 to 4·7)	1·8 (−0·9 to 4·1)	0·9 (−2·7 to 4·9)	−1·2 (−4·1 to 2·0)
Argentina	High-middle SDI	14·6 (12·7 to 16·6)	21·1 (18·6 to 23·6)	−1·1 (−1·9 to −0·4)	−1·0 (−1·7 to −0·3)	−1·0 (−2·2 to 0·2)	−1·0 (−2·0 to 0·1)	−1·2 (−3·0 to 0·4)	−1·1 (−2·6 to 0·4)
Armenia	High-middle SDI	1·5 (1·1 to 2·1)	43·5 (40·0 to 46·9)	0·3 (−1·5 to 2·2)	0·0 (−0·4 to 0·5)	1·1 (−1·7 to 4·0)	0·6 (−0·1 to 1·3)	−0·8 (−4·5 to 3·1)	−0·7 (−1·7 to 0·1)
Australia	High SDI	13·3 (12·4 to 14·3)	15·6 (14·5 to 16·6)	−2·1 (−2·4 to −1·8)	−1·9 (−2·2 to −1·6)	−2·3 (−2·7 to −1·9)	−1·7 (−2·1 to −1·4)	−1·9 (−2·7 to −1·1)	−2·2 (−3·0 to −1·4)
Austria	High SDI	22·7 (20·2 to 25·5)	30·0 (27·4 to 32·6)	0·3 (−0·3 to 1·0)	−0·3 (−0·7 to 0·2)	1·1 (0·2 to 1·9)	−0·3 (−0·9 to 0·3)	−0·8 (−2·1 to 0·5)	−0·2 (−1·3 to 0·8)
Azerbaijan	High-middle SDI	1·6 (1·1 to 2·1)	40·2 (36·5 to 43·7)	1·1 (−0·7 to 2·7)	0·9 (0·3 to 1·5)	0·9 (−2·0 to 3·7)	0·6 (−0·3 to 1·5)	1·3 (−2·7 to 5·2)	1·3 (0·1 to 2·4)
Bahrain	High-middle SDI	6·2 (4·4 to 8·9)	12·1 (10·1 to 14·3)	−0·2 (−2·2 to 1·7)	−1·3 (−2·3 to −0·4)	−0·5 (−3·6 to 2·5)	−1·9 (−3·3 to −0·5)	0·2 (−3·7 to 4·5)	−0·3 (−2·4 to 1·7)
Bangladesh	Low-middle SDI	1·8 (1·1 to 2·6)	38·0 (34·1 to 42·6)	−2·9 (−5·2 to −0·4)	0·3 (−0·4 to 1·0)	−1·9 (−5·2 to 1·7)	0·4 (−0·5 to 1·4)	−4·3 (−9·7 to 0·6)	0·0 (−1·2 to 1·3)
Barbados	High-middle SDI	2·1 (1·5 to 3·0)	6·9 (5·4 to 8·9)	1·3 (−0·6 to 3·3)	0·3 (−1·1 to 1·7)	1·5 (−1·5 to 4·6)	1·0 (−1·0 to 3·2)	1·1 (−3·2 to 5·5)	−0·7 (−3·8 to 2·2)
Belarus	High SDI	13·5 (11·4 to 15·9)	42·4 (39·7 to 45·1)	0·7 (−0·3 to 1·8)	−0·8 (−1·2 to −0·5)	0·3 (−1·6 to 2·1)	−1·5 (−2·0 to −0·9)	1·3 (−1·0 to 3·8)	0·1 (−0·7 to 1·0)
Belgium	High SDI	16·7 (15·0 to 18·4)	21·2 (19·4 to 23·2)	−1·1 (−1·6 to −0·6)	−1·6 (−2·0 to −1·2)	−1·0 (−1·8 to −0·2)	−1·5 (−2·1 to −0·9)	−1·3 (−2·7 to 0·1)	−1·7 (−2·8 to −0·5)
Belize	Middle SDI	2·1 (1·6 to 2·9)	13·3 (10·7 to 16·0)	−0·9 (−2·5 to 0·9)	−1·0 (−2·2 to 0·1)	−1·2 (−3·9 to 1·6)	−1·4 (−3·0 to 0·4)	−0·4 (−4·1 to 3·5)	−0·6 (−3·0 to 1·7)
Benin	Low SDI	1·0 (0·7 to 1·5)	8·6 (7·3 to 10·2)	−3·8 (−6·2 to −1·6)	−0·8 (−1·8 to 0·2)	−4·4 (−7·9 to −1·0)	−0·5 (−1·9 to 0·9)	−3·0 (−7·7 to 1·6)	−1·3 (−3·4 to 0·8)
Bermuda	High SDI	4·7 (3·5 to 6·3)	13·3 (10·8 to 16·1)	−1·1 (−2·8 to 0·5)	−1·0 (−2·2 to 0·2)	−1·5 (−4·1 to 1·3)	−1·0 (−2·8 to 1·0)	−0·6 (−4·2 to 3·0)	−0·9 (−3·6 to 1·6)
Bhutan	Low-middle SDI	3·8 (2·9 to 4·8)	8·5 (7·2 to 9·9)	0·3 (−1·8 to 2·4)	−0·5 (−1·6 to 0·7)	−0·2 (−3·9 to 3·6)	−1·1 (−3·0 to 1·1)	1·0 (−3·3 to 5·5)	0·4 (−2·1 to 3·0)
Bolivia	Middle SDI	8·8 (7·1 to 10·7)	32·1 (27·5 to 37·1)	−1·1 (−2·3 to 0·0)	−0·3 (−1·1 to 0·5)	−0·4 (−2·0 to 1·3)	0·8 (−0·1 to 1·9)	−2·2 (−4·5 to 0·1)	−1·9 (−3·5 to −0·4)
Bosnia and Herzegovina	High-middle SDI	21·1 (18·0 to 24·5)	36·0 (33·3 to 38·7)	0·5 (−0·4 to 1·5)	0·2 (−0·2 to 0·7)	0·5 (−0·8 to 1·9)	0·6 (0·0 to 1·3)	0·5 (−1·2 to 2·1)	−0·3 (−1·1 to 0·5)
Botswana	Middle SDI	4·3 (3·2 to 5·5)	18·3 (16·3 to 20·5)	−1·0 (−2·4 to 0·5)	−0·4 (−1·1 to 0·2)	−1·1 (−3·4 to 1·2)	−0·6 (−1·5 to 0·3)	−0·8 (−4·1 to 2·3)	−0·2 (−1·5 to 1·1)
Brazil	Middle SDI	8·2 (7·5 to 9·0)	12·6 (11·8 to 13·5)	−3·3 (−3·9 to −2·7)	−3·3 (−3·8 to −2·9)	−3·4 (−4·3 to −2·6)	−3·8 (−4·4 to −3·2)	−3·0 (−4·1 to −1·9)	−2·6 (−3·5 to −1·8)
Brunei	High SDI	3·7 (3·1 to 4·4)	19·8 (18·0 to 21·8)	−0·3 (−1·2 to 0·6)	−0·7 (−1·3 to −0·2)	−0·4 (−1·8 to 1·1)	−0·8 (−1·7 to −0·1)	−0·3 (−2·3 to 1·7)	−0·6 (−1·7 to 0·5)
Bulgaria	High-middle SDI	28·3 (24·5 to 32·0)	35·2 (32·4 to 38·0)	0·6 (−0·3 to 1·5)	−0·6 (−1·0 to −0·1)	1·3 (0·1 to 2·8)	0·0 (−0·6 to 0·6)	−0·5 (−2·1 to 1·1)	−1·4 (−2·4 to −0·5)
Burkina Faso	Low SDI	4·2 (2·8 to 6·3)	12·5 (10·7 to 14·9)	−1·0 (−3·4 to 1·2)	−0·6 (−1·5 to 0·4)	−1·0 (−4·3 to 2·4)	−0·6 (−2·2 to 1·0)	−1·0 (−5·3 to 3·5)	−0·5 (−2·6 to 1·7)
Burundi	Low SDI	0·9 (0·7 to 1·2)	9·7 (7·9 to 11·7)	−1·1 (−2·4 to 0·2)	−0·9 (−2·0 to 0·2)	−1·5 (−3·4 to 0·5)	−1·3 (−3·1 to 0·3)	−0·6 (−3·2 to 2·1)	−0·3 (−2·8 to 2·3)
Cambodia	Low-middle SDI	3·8 (2·8 to 5·1)	34·2 (31·8 to 36·6)	−1·8 (−3·7 to 0·2)	−1·0 (−1·4 to −0·6)	−2·1 (−4·5 to 0·5)	−0·5 (−1·0 to 0·1)	−1·3 (−4·8 to 1·9)	−1·8 (−2·6 to −1·0)
Cameroon	Low-middle SDI	1·6 (1·2 to 2·1)	8·3 (7·1 to 9·6)	−1·5 (−3·6 to 0·6)	−1·1 (−2·0 to −0·2)	−2·0 (−5·7 to 1·7)	−0·9 (−2·4 to 0·8)	−0·7 (−4·8 to 3·8)	−1·6 (−3·7 to 0·6)
Canada	High SDI	12·4 (10·8 to 14·2)	14·5 (12·6 to 16·7)	−2·8 (−3·3 to −2·2)	−2·5 (−3·0 to −1·9)	−3·7 (−4·4 to −3·1)	−3·0 (−3·6 to −2·4)	−1·4 (−2·8 to 0·0)	−1·6 (−3·1 to 0·0)
Cape Verde	Low-middle SDI	2·5 (1·7 to 3·6)	9·8 (8·0 to 11·7)	−0·9 (−3·1 to 1·3)	−0·6 (−1·6 to 0·6)	−1·1 (−4·3 to 2·4)	−0·7 (−2·2 to 0·8)	−0·6 (−5·2 to 4·0)	−0·3 (−2·5 to 1·8)
Central African Republic	Low SDI	1·4 (0·8 to 2·2)	11·6 (10·1 to 13·4)	−0·8 (−3·6 to 1·9)	0·3 (−0·4 to 1·1)	−1·2 (−6·1 to 3·0)	0·2 (−1·0 to 1·3)	−0·2 (−6·5 to 6·1)	0·6 (−1·0 to 2·2)
Chad	Low SDI	1·9 (1·3 to 2·8)	11·5 (9·6 to 13·8)	−0·8 (−3·0 to 1·4)	−0·2 (−1·2 to 0·8)	−0·8 (−4·1 to 2·5)	−0·2 (−1·5 to 1·3)	−0·7 (−5·2 to 3·8)	−0·2 (−2·2 to 1·5)
Chile	High-middle SDI	22·7 (20·1 to 25·3)	27·7 (24·8 to 30·8)	0·9 (0·2 to 1·6)	0·3 (−0·3 to 1)	1·9 (1·0 to 2·9)	1·2 (0·4 to 2·0)	−0·6 (−2·0 to 0·7)	−1·0 (−2·3 to 0·3)
China	Middle SDI	2·2 (2·1 to 2·4)	37·5 (36·9 to 38·0)	−2·6 (−3·2 to −2·1)	−1·0 (−1·1 to −0·9)	−3·3 (−4·1 to −2·5)	−1·0 (−1·2 to −0·9)	−1·6 (−2·7 to −0·4)	−1·0 (−1·2 to −0·8)
Colombia	High-middle SDI	6·0 (4·4 to 7·8)	14·4 (11·6 to 17·5)	−2·2 (−3·7 to −0·6)	−1·8 (−2·9 to −0·7)	−1·8 (−4·1 to 0·4)	−1·4 (−3·0 to 0·2)	−2·8 (−6·4 to 0·9)	−2·3 (−4·6 to 0·1)
Comoros	Low SDI	1·2 (1·0 to 1·5)	14·0 (11·9 to 16·2)	−0·8 (−1·9 to 0·5)	−0·2 (−1·2 to 0·9)	−1·0 (−2·8 to 1·0)	−0·1 (−1·7 to 1·7)	−0·5 (−3·1 to 2·0)	−0·4 (−2·8 to 1·9)
Congo (Brazzaville)	Low-middle SDI	1·2 (0·7 to 1·9)	11·0 (9·5 to 12·7)	0·5 (−2·3 to 3·5)	0·9 (0·1 to 1·7)	0·1 (−4·3 to 4·7)	0·5 (−0·6 to 1·7)	1·1 (−5·1 to 7·1)	1·6 (0·1 to 3·3)
Costa Rica	High-middle SDI	4·8 (3·5 to 6·3)	10·4 (8·3 to 12·7)	−1·1 (−2·8 to 0·7)	−1·8 (−2·9 to −0·7)	−1·1 (−3·7 to 1·4)	−2·0 (−3·4 to −0·5)	−1·1 (−4·4 to 2·5)	−1·5 (−3·9 to 0·7)
Côte d'Ivoire	Low SDI	1·4 (0·9 to 2·0)	14·2 (12·0 to 16·5)	−2·4 (−4·7 to −0·1)	0·8 (−0·2 to 1·8)	−2·1 (−5·8 to 1·5)	0·4 (−1·1 to 2·0)	−3·0 (−7·8 to 1·5)	1·3 (−0·7 to 3·3)
Croatia	High-middle SDI	25·9 (22·3 to 29·7)	30·4 (27·7 to 33·3)	0·0 (−0·9 to 0·8)	−0·9 (−1·4 to −0·4)	−0·8 (−2·0 to 0·4)	−1·3 (−1·9 to −0·6)	1·2 (−0·6 to 2·9)	−0·4 (−1·4 to 0·7)
Cuba	High-middle SDI	9·4 (7·2 to 11·9)	20·9 (17·4 to 24·8)	−2·3 (−3·7 to −0·9)	−2·0 (−3·0 to −1·1)	−1·5 (−3·7 to 0·6)	−1·5 (−2·8 to −0·1)	−3·6 (−6·6 to −0·8)	−2·9 (−4·8 to −0·9)
Cyprus	High SDI	14·5 (12·5 to 16·5)	37·5 (34·6 to 40·5)	0·5 (−0·4 to 1·4)	0·5 (0·0 to 1·0)	1·1 (−0·2 to 2·5)	1·1 (0·4 to 1·8)	−0·4 (−2·2 to 1·3)	−0·5 (−1·5 to 0·4)
Czech Republic	High SDI	19·4 (16·6 to 22·3)	28·7 (26·0 to 31·1)	−0·5 (−1·3 to 0·3)	−0·6 (−1·1 to −0·1)	−0·8 (−1·8 to 0·4)	−1·0 (−1·7 to −0·3)	−0·2 (−1·9 to 1·5)	0·0 (−1·0 to 1·0)
Democratic Republic of the Congo	Low SDI	0·9 (0·5 to 1·4)	14·1 (12·6 to 15·6)	−1·0 (−3·9 to 1·8)	−0·1 (−0·7 to 0·6)	−1·1 (−5·9 to 3·6)	−0·3 (−1·4 to 0·9)	−1·0 (−7·7 to 5·1)	0·2 (−1·3 to 1·7)
Denmark	High SDI	16·2 (14·7 to 17·6)	17·5 (16·1 to 19·1)	−3·0 (−3·4 to −2·6)	−3·0 (−3·4 to −2·6)	−3·5 (−3·9 to −3·1)	−2·4 (−2·8 to −2·0)	−2·3 (−3·3 to −1·3)	−3·8 (−4·8 to −2·8)
Djibouti	Low-middle SDI	2·8 (2·2 to 3·4)	21·6 (18·2 to 25·0)	0·0 (−1·2 to 1·3)	−0·5 (−1·5 to 0·5)	0·1 (−2·0– to 1·9)	−0·5 (−1·8 to 1·0)	0·0 (−2·4 to 2·6)	−0·6 (−2·5 to 1·2)
Dominica	High-middle SDI	1·2 (0·9 to 1·7)	6·5 (5·0 to 8·3)	−0·9 (−2·8 to 1·0)	−0·7 (−2·2 to 0·7)	−0·7 (−3·7 to 2·2)	−0·6 (−2·7 to 1·7)	−1·2 (−5·3 to 2·8)	−0·9 (−3·9 to 2·0)
Dominican Republic	High-middle SDI	5·2 (3·9 to 7·0)	8·7 (7·1 to 10·6)	−2·7 (−4·2 to −1·2)	−2·4 (−3·6 to −1·2)	−2·5 (−4·6 to −0·5)	−2·3 (−3·9 to −0·7)	−3·1 (−6·1 to −0·1)	−2·5 (−4·6 to −0·3)
Ecuador	High-middle SDI	1·9 (1·5 to 2·3)	8·9 (7·5 to 10·6)	−1·8 (−2·9 to −0·6)	−2·3 (−3·3 to −1·3)	−0·4 (−2·3 to 1·5)	−2·9 (−4·5 to −1·3)	−3·8 (−6·2 to −1·4)	−1·4 (−3·4 to 0·7)
Egypt	Middle SDI	0·6 (0·4 to 0·8)	31·7 (28·8 to 35·0)	−0·9 (−2·9 to 1·4)	0·2 (−0·4 to 0·9)	−1·1 (−4·4 to 2·5)	0·0 (−0·9 to 0·9)	−0·6 (−5·2 to 3·6)	0·6 (−0·7 to 1·9)
El Salvador	Middle SDI	3·3 (2·4 to 4·5)	10·0 (7·8 to 12·4)	−1·0 (−3·0 to 0·8)	−0·3 (−1·6 to 1·0)	−1·0 (−3·8 to 1·6)	−0·3 (−2·5 to 1·7)	−1·0 (−4·7 to 2·6)	−0·2 (−3·1 to 2·7)
Equatorial Guinea	Middle SDI	1·2 (0·7 to 1·9)	8·6 (7·5 to 9·9)	−0·4 (−3·3 to 2·5)	0·0 (−0·7 to 0·9)	−0·6 (−5·3 to 3·9)	0·0 (−1·2 to 1·2)	0·0 (−6·2 to 6·2)	0·1 (−1·7 to 1·8)
Eritrea	Low SDI	0·6 (0·5 to 0·8)	10·2 (8·3 to 12·4)	−1·7 (−2·9 to −0·5)	−0·9 (−2·1 to 0·2)	−1·6 (−3·5 to 0·2)	−0·9 (−2·4 to 0·5)	−1·7 (−4·2 to 0·7)	−1·0 (−3·2 to 1·1)
Estonia	High SDI	14·8 (12·8 to 16·9)	30·2 (28·0 to 32·3)	−0·7 (−1·4 to 0·0)	−0·9 (−1·3 to −0·6)	−0·3 (−1·1 to 0·5)	−0·3 (−0·7 to 0·1)	−1·4 (−3 to 0·2)	−1·9 (−2·8 to −1·1)
Ethiopia	Low SDI	0·8 (0·7 to 1·0)	7·1 (5·6 to 8·7)	−1·5 (−2·8 to −0·3)	−0·4 (−1·7 to 0·8)	−1·4 (−3·2 to 0·4)	−0·9 (−2·7 to 1·0)	−1·8 (−4·3 to 0·6)	0·3 (−2·1 to 2·6)
Federated States of Micronesia	Middle SDI	6·5 (5·1 to 8·1)	20·8 (17·7 to 24·2)	−0·2 (−1·5 to 1·1)	−0·5 (−1·3 to 0·3)	−0·2 (−2·1 to 1·7)	−0·5 (−1·8 to 0·8)	−0·2 (−2·9 to 2·6)	−0·4 (−2·2 to 1·4)
Fiji	High-middle SDI	4·2 (3·4 to 5·2)	17·5 (15·3 to 19·9)	−0·2 (−1·3 to 1·0)	−0·5 (−1·3 to 0·3)	−0·5 (−2·3 to 1·4)	−0·4 (−1·8 to 0·9)	0·4 (−2·1 to 2·8)	−0·5 (−2·2 to 1·1)
Finland	High SDI	15·5 (13·8 to 17·4)	19·3 (17·4 to 21·1)	−0·4 (−0·9 to 0·2)	−1·4 (−1·9 to −1·0)	−0·4 (−1·0 to 0·1)	−1·5 (−1·8 to −1·2)	−0·4 (−1·7 to 0·9)	−1·3 (−2·4 to −0·4)
France	High SDI	21·5 (19·2 to 23·9)	25·3 (22·9 to 27·6)	−0·6 (−1·2 to −0·1)	−1·5 (−2·0 to −1·1)	−0·4 (−1·2 to 0·3)	−1·5 (−2·1 to −0·9)	−1·0 (−2·3 to 0·4)	−1·6 (−2·7 to −0·4)
Gabon	Middle SDI	2·2 (1·3 to 3·6)	14·7 (13·1 to 16·4)	−0·2 (−2·9 to 2·6)	0·4 (−0·2 to 1·2)	0·0 (−4·4 to 4·2)	0·6 (−0·5 to 1·8)	−0·3 (−6·1 to 5·4)	0·1 (−1·4 to 1·6)
Georgia	High-middle SDI	3·8 (2·9 to 4·8)	38·9 (35·5 to 42·2)	−0·1 (−1·7 to 1·5)	0·5 (−0·1 to 1·0)	−0·3 (−2·8 to 2·3)	0·8 (0·0 to 1·6)	0·1 (−3·1 to 3·4)	−0·1 (−1·2 to 1·0)
Germany	High SDI	19·4 (17·3 to 21·7)	25·2 (22·8 to 27·4)	−0·3 (−0·9 to 0·2)	−0·9 (−1·4 to −0·5)	−0·2 (−0·8 to 0·4)	−1·1 (−1·6 to −0·6)	−0·5 (−1·9 to 0·7)	−0·6 (−1·7 to 0·4)
Ghana	Low-middle SDI	0·9 (0·6 to 1·3)	5·8 (4·8 to 6·9)	−0·8 (−2·9 to 1·4)	−1·1 (−2·1 to 0·0)	−0·8 (−3·9 to 2·4)	−0·9 (−2·4 to 0·7)	−0·9 (−5·4 to 3·6)	−1·4 (−3·4 to 0·5)
Greece	High-middle SDI	27·2 (24·6 to 29·6)	36·6 (34·0 to 39·0)	0·5 (−0·1 to 0·9)	−0·8 (−1·1 to −0·5)	1·2 (0·5 to 1·9)	−0·8 (−1·3 to −0·4)	−0·6 (−1·7 to 0·5)	−0·7 (−1·5 to 0·1)
Greenland	High-middle SDI	44·3 (41·1 to 47·6)	42·7 (39·4 to 45·9)	−0·8 (−1·1 to −0·4)	−1·0 (−1·4 to −0·6)	−0·8 (−1·3 to −0·2)	−1·1 (−1·6 to −0·5)	−0·7 (−1·6 to 0·1)	−0·8 (−1·7 to 0·1)
Grenada	High-middle SDI	2·5 (1·8 to 3·4)	10·5 (8·3 to 13·1)	−0·6 (−2·5 to 1·2)	0·3 (−1·0 to 1·6)	−0·3 (−3·1 to 2·5)	1·0 (−1·1 to 3·0)	−1·1 (−4·9 to 2·7)	−0·7 (−3·4 to 2·0)
Guam	High SDI	14·5 (12·1 to 17·1)	22·1 (19·4 to 24·8)	−1·0 (−2·1 to 0·0)	−0·9 (−1·6 to −0·1)	−0·5 (−2·1 to 1·1)	−0·7 (−1·8 to 0·4)	−1·7 (−4·0 to 0·6)	−1·2 (−2·8 to 0·4)
Guatemala	Low-middle SDI	2·5 (1·8 to 3·4)	13·4 (10·8 to 16·4)	−1·0 (−2·7 to 0·8)	0·3 (−0·9 to 1·6)	−1·7 (−4·4 to 1·0)	0·0 (−1·8 to 1·8)	0·2 (−3·5 to 3·8)	0·9 (−1·7 to 3·4)
Guinea	Low SDI	1·4 (0·9 to 2·1)	6·9 (5·6 to 8·4)	−1·0 (−3·5 to 1·3)	−0·6 (−1·7 to 0·5)	−1·4 (−5·0 to 2·3)	−0·8 (−2·5 to 1·0)	−0·6 (−5·7 to 4·1)	−0·4 (−2·9 to 2·0)
Guinea-Bissau	Low SDI	1·0 (0·6 to 1·5)	11·4 (9·4 to 13·5)	−0·9 (−3·4 to 1·6)	−0·3 (−1·4 to 0·8)	−1·2 (−5·2 to 2·6)	−0·5 (−2·1 to 1·1)	−0·4 (−5·4 to 4·7)	0·1 (−2·2 to 2·2)
Guyana	Middle SDI	2·0 (1·4 to 2·8)	15·8 (13·0 to 18·9)	−0·9 (−2·7 to 0·9)	0·8 (−0·3 to 1·9)	−0·1 (−3·0 to 2·7)	2·3 (0·6 to 4·1)	−2·2 (−6·1 to 1·7)	−1·5 (−3·9 to 0·8)
Haiti	Low-middle SDI	3·2 (2·3 to 4·3)	8·2 (6·6 to 10·1)	−1·5 (−3·3 to 0·2)	−2·6 (−3·8 to −1·4)	−2·1 (−4·7 to 0·7)	−2·8 (−4·7 to −0·8)	−0·6 (−4·2 to 3·0)	−2·4 (−5·0 to 0·1)
Honduras	Middle SDI	1·8 (1·2 to 2·4)	16·4 (13·8 to 19·2)	−3·2 (−5·1 to −1·4)	−1·0 (−2·0 to 0·0)	−3·9 (−6·7 to −1·0)	−0·8 (−2·4 to 0·9)	−2·2 (−6·3 to 1·9)	−1·3 (−3·5 to 0·9)
Hungary	High SDI	22·8 (19·5 to 26·1)	27·5 (25·0 to 29·9)	−0·1 (−1·0 to 0·8)	−1·1 (−1·7 to −0·7)	0·4 (−0·8 to 1·7)	−0·8 (−1·5 to −0·1)	−0·9 (−2·8 to 0·9)	−1·7 (−2·7 to −0·6)
Iceland	High SDI	14·4 (12·5 to 16·4)	14·5 (13·0 to 16·3)	−2·8 (−3·4 to −2·2)	−2·8 (−3·4 to −2·3)	−3·2 (−3·9 to −2·5)	−2·9 (−3·5 to −2·2)	−2·2 (−3·7 to −0·7)	−2·8 (−4·2 to −1·4)
India	Low-middle SDI	2·8 (2·6 to 3·2)	17·4 (16·8 to 18·2)	−0·3 (−1·0 to 0·3)	−2·1 (−2·3 to −1·8)	1·0 (−0·3 to 2·2)	−1·4 (−1·8 to −1·0)	−2·2 (−3·7 to −0·7)	−3·1 (−3·7 to −2·4)
Indonesia	Middle SDI	3·8 (2·7 to 5·1)	46·7 (43·9 to 49·5)	1·8 (0·0 to 3·7)	0·2 (−0·1 to 0·6)	5·3 (2·7 to 8·1)	0·3 (−0·2 to 0·9)	−3·4 (−7·1 to 0·2)	0·1 (−0·7 to 0·9)
Iran	High-middle SDI	2·1 (1·4 to 3·0)	17·9 (15·3 to 20·6)	−0·8 (−3·0 to 1·4)	0·1 (−0·8 to 1·0)	−1·6 (−4·6 to 1·4)	0·3 (−0·9 to 1·5)	0·3 (−3·7 to 4·6)	−0·2 (−1·9 to 1·5)
Iraq	Middle SDI	3·0 (2·0 to 4·3)	23·8 (20·4 to 27·6)	0·1 (−2·1 to 2·3)	−0·4 (−1·2 to 0·4)	0·5 (−2·7 to 4·0)	−0·4 (−1·4 to 0·7)	−0·6 (−4·7 to 3·5)	−0·5 (−2·2 to 1·0)
Ireland	High SDI	21·9 (19·5 to 24·5)	20·6 (18·4 to 22·9)	−0·5 (−1·1 to 0·0)	−1·4 (−1·9 to −0·9)	−0·7 (−1·5 to 0·1)	−1·4 (−2·0 to −0·7)	−0·3 (−1·6 to 1·1)	−1·4 (−2·7 to −0·2)
Israel	High SDI	13·0 (11·4 to 14·9)	23·4 (21·0 to 26·0)	−1·6 (−2·3 to −0·8)	−1·2 (−1·8 to −0·6)	−2·6 (−3·7 to −1·6)	−1·8 (−2·6 to −0·9)	0·0 (−1·6 to 1·6)	−0·4 (−1·7 to 0·8)
Italy	High SDI	17·1 (15·3 to 19·0)	23·2 (21·2 to 25·5)	−0·9 (−1·5 to −0·3)	−1·1 (−1·5 to −0·6)	−0·8 (−1·5 to −0·2)	−1·2 (−1·6 to −0·7)	−1·0 (−2·2 to 0·2)	−0·9 (−1·9 to 0·2)
Jamaica	High-middle SDI	6·3 (4·8 to 8·1)	12·7 (10·1 to 15·7)	0·2 (−1·4 to 1·8)	−1·4 (−2·6 to −0·2)	0·8 (−1·5 to 3·2)	−0·4 (−2·0 to 1·4)	−0·7 (−3·8 to 2·3)	−2·9 (−5·3 to −0·5)
Japan	High SDI	9·3 (8·9 to 9·6)	26·6 (26·1 to 27·1)	−0·7 (−0·9 to −0·5)	−2·4 (−2·5 to −2·3)	0·7 (0·4 to 1·0)	−1·7 (−1·8 to −1·6)	−2·8 (−3·2 to −2·3)	−3·4 (−3·6 to −3·2)
Jordan	High-middle SDI	6·8 (5·1 to 8·8)	30·7 (26·9 to 34·6)	−0·4 (−2·2 to 1·4)	−0·1 (−0·8 to 0·6)	−0·7 (−3·3 to 2·0)	0·6 (−0·3 to 1·7)	0·0 (−3·5 to 3·3)	−1·2 (−2·5 to 0·2)
Kazakhstan	High-middle SDI	4·1 (3·2 to 5·1)	37·0 (34·4 to 39·5)	0·4 (−1·2 to 2·0)	−0·1 (−0·6 to 0·4)	1·8 (−0·8 to 4·5)	−0·1 (−0·9 to 0·7)	−1·7 (−4·9 to 1·6)	−0·1 (−1·2 to 0·9)
Kenya	Low SDI	1·0 (1·0 to 1·1)	14·9 (14·4 to 15·4)	−1·7 (−2·0 to −1·5)	−0·8 (−1·0 to −0·6)	−1·3 (−1·7 to −1·0)	−0·5 (−0·8 to −0·2)	−2·4 (−2·8 to −1·9)	−1·4 (−1·8 to −0·9)
Kiribati	Low-middle SDI	24·7 (21·3 to 28·4)	47·8 (43·8 to 51·5)	−0·6 (−1·4 to 0·1)	−0·3 (−0·7 to 0·2)	0·1 (−0·9 to 1·2)	0·1 (−0·5 to 0·8)	−1·8 (−3·4 to −0·2)	−0·8 (−1·8 to 0·1)
Kuwait	High SDI	5·2 (4·1 to 6·5)	23·2 (20·9 to 25·6)	3·6 (1·7 to 5·4)	−0·6 (−1·2 to 0·1)	1·7 (−1·1 to 4·8)	−0·6 (−1·7 to 0·4)	6·4 (3·1 to 9·8)	−0·6 (−1·9 to 0·7)
Kyrgyzstan	Middle SDI	2·8 (2·1 to 3·8)	32·9 (30·1 to 35·8)	2·9 (1·2 to 4·7)	0·2 (−0·3 to 0·7)	3·0 (0·4 to 5·6)	0·3 (−0·5 to 1·1)	2·7 (−0·8 to 6·5)	0·0 (−1·0 to 1·2)
Laos	Low-middle SDI	9·7 (6·9 to 13·3)	46·5 (42·6 to 50·3)	−0·1 (−2·0 to 1·7)	−0·3 (−0·8 to 0·1)	0·5 (−1·7 to 3)	−0·1 (−0·7 to 0·5)	−1·0 (−4·5 to 2·4)	−0·6 (−1·5 to 0·2)
Latvia	High SDI	16·1 (13·8 to 18·6)	38·3 (35·9 to 40·6)	0·2 (−0·7 to 1·2)	0·1 (−0·3 to 0·5)	0·7 (−0·6 to 2·1)	0·6 (0 to 1·2)	−0·4 (−2·1 to 1·2)	−0·6 (−1·3 to 0·1)
Lebanon	High-middle SDI	17·9 (13·8 to 22·8)	28·0 (24·5 to 31·8)	−0·2 (−1·6 to 1·3)	−1·7 (−2·4 to −1·1)	−0·2 (−2·2 to 1·9)	−2·1 (−2·9 to −1·2)	−0·2 (−3·2 to 2·6)	−1·2 (−2·7 to 0·2)
Lesotho	Low-middle SDI	0·9 (0·7 to 1·1)	28·0 (25·7 to 30·5)	−1·9 (−3·6 to −0·3)	−0·4 (−0·9 to 0·1)	−2·5 (−5·1 to 0·2)	−0·4 (−1·1 to 0·3)	−1·0 (−4·7 to 2·6)	−0·4 (−1·5 to 0·6)
Liberia	Low SDI	0·9 (0·6 to 1·4)	10·4 (9·0 to 12·0)	−2·5 (−4·9 to −0·2)	−0·6 (−1·5 to 0·3)	−2·8 (−6·5 to 0·8)	−0·2 (−1·7 to 1·2)	−2·0 (−7·5 to 3·4)	−1·2 (−3·4 to 0·7)
Libya	Middle SDI	0·4 (0·2 to 0·6)	24·8 (21·8 to 28·1)	−0·3 (−2·8 to 2·3)	0·2 (−0·6 to 0·9)	−0·7 (−4·4 to 3·2)	0·4 (−0·6 to 1·5)	0·3 (−4·9 to 5·5)	−0·1 (−1·7 to 1·5)
Lithuania	High SDI	14·0 (12·0 to 16·5)	32·8 (30·4 to 35·2)	0·2 (−0·7 to 1·2)	−0·4 (−0·8 to 0·0)	0·1 (−1·2 to 1·4)	−0·4 (−0·9 to 0·2)	0·4 (−1·5 to 2·1)	−0·4 (−1·4 to 0·4)
Luxembourg	High SDI	18·5 (16·0 to 21·3)	23·8 (21·0 to 26·5)	−0·7 (−1·5 to −0·1)	−0·9 (−1·5 to −0·4)	−0·5 (−1·6 to 0·4)	−0·7 (−1·4 to 0·1)	−1·1 (−2·6 to 0·6)	−1·3 (−2·6 to −0·1)
Macedonia	High-middle SDI	23·2 (20·1 to 26·6)	36·1 (33·4 to 38·9)	1·0 (−0·1 to 1·9)	0·4 (−0·1 to 0·9)	1·2 (−0·3 to 2·7)	0·7 (0·0 to 1·4)	0·7 (−1·3 to 2·8)	0·0 (−1·0 to 1·0)
Madagascar	Low SDI	1·5 (1·2 to 1·9)	19·0 (15·9 to 22·2)	−3·8 (−5·0 to −2·6)	−1·6 (−2·5 to −0·6)	−3·8 (−5·5 to −1·9)	−1·2 (−2·4 to 0·1)	−3·9 (−6·3 to −1·1)	−2·2 (−4·2 to −0·3)
Malawi	Low SDI	1·4 (1·1 to 1·8)	15·3 (13·0 to 17·7)	−1·7 (−3·1 to −0·4)	−1·2 (−2·2 to −0·3)	−1·5 (−3·5 to 0·5)	−0·9 (−2·3 to 0·5)	−2·2 (−4·9 to 0·8)	−1·8 (−3·8 to 0·0)
Malaysia	High-middle SDI	1·7 (1·2 to 2·3)	31·9 (28·8 to 35·1)	−1·7 (−3·7 to 0·4)	−0·7 (−1·2 to −0·1)	−1·9 (−5·0 to 1·4)	−0·6 (−1·4 to 0·2)	−1·3 (−5·4 to 2·6)	−0·7 (−2·0 to 0·4)
Maldives	Middle SDI	6·8 (5·1 to 8·7)	30·8 (27·7 to 33·9)	−1·2 (−2·9 to 0·6)	0·7 (0·1 to 1·4)	−0·7 (−3·3 to 2·2)	0·4 (−0·7 to 1·5)	−1·9 (−5·6 to 1·7)	1·2 (−0·2 to 2·6)
Mali	Low SDI	0·7 (0·5 to 1·1)	10·8 (9·1 to 12·7)	−4·6 (−6·8 to −2·3)	0·2 (−0·9 to 1·1)	−3·7 (−7·4 to −0·2)	0·1 (−1·4 to 1·8)	−5·9 (−10·8 to −1·0)	0·2 (−1·9 to 2·4)
Malta	High-middle SDI	15·1 (13·1 to 17·1)	23·4 (20·8 to 25·9)	−1·4 (−2·2 to −0·6)	−1·9 (−2·5 to −1·3)	−1·9 (−3·0 to −0·8)	−2·4 (−3·2 to −1·7)	−0·6 (−2·3 to 0·9)	−1·1 (−2·4 to 0·2)
Marshall Islands	Middle SDI	4·2 (3·4 to 5·2)	22·8 (19·4 to 26·5)	−0·5 (−1·8 to 0·9)	−0·7 (−1·6 to 0·1)	−0·7 (−2·6 to 1·2)	−0·6 (−1·8 to 0·6)	−0·3 (−2·8 to 2·4)	−0·8 (−2·5 to 0·6)
Mauritania	Low SDI	2·4 (1·5 to 3·6)	14·9 (12·4 to 17·8)	−0·7 (−3·3 to 1·6)	−0·2 (−1·2 to 0·7)	−0·8 (−4·2 to 2·7)	0·2 (−1·2 to 1·7)	−0·6 (−5·5 to 3·5)	−0·9 (−2·9 to 1·1)
Mauritius	High-middle SDI	2·7 (1·8 to 3·9)	28·3 (24·9 to 31·7)	−0·8 (−3·0 to 1·4)	−1·0 (−1·6 to −0·4)	−2·5 (−6·0 to 0·9)	−1·8 (−2·6 to −1·0)	1·8 (−2·9 to 6·0)	0·3 (−1·2 to 1·5)
Mexico	Middle SDI	4·8 (4·5 to 5·2)	15·0 (14·4 to 15·7)	−3·2 (−3·6 to −2·7)	−2·5 (−2·7 to −2·3)	−5·5 (−6·1 to −4·8)	−4·2 (−4·5 to −3·8)	0·3 (−0·6 to 1·2)	0·0 (−0·5 to 0·5)
Moldova	High-middle SDI	5·1 (4·2 to 6·1)	32·5 (30·6 to 34·6)	0·4 (−0·9 to 1·6)	−0·9 (−1·3 to −0·5)	0·3 (−1·8 to 2·3)	−1·0 (−1·6 to −0·4)	0·5 (−2·3 to 3·2)	−0·7 (−1·6 to 0·2)
Mongolia	High-middle SDI	5·1 (3·9 to 6·4)	37·0 (33·4 to 40·5)	0·4 (−1·1 to 2·0)	−0·3 (−0·8 to 0·2)	0·3 (−1·7 to 2·4)	0·1 (−0·5 to 0·8)	0·5 (−2·7 to 3·4)	−0·8 (−1·9 to 0·2)
Montenegro	High-middle SDI	26·4 (23·4 to 29·5)	33·0 (30·6 to 35·5)	1·7 (0·9 to 2·6)	0·9 (0·4 to 1·3)	2·2 (0·6 to 3·7)	1·5 (0·7 to 2·4)	1·0 (−0·8 to 2·8)	−0·2 (−1·2 to 0·8)
Morocco	Low-middle SDI	0·9 (0·6 to 1·3)	16·0 (13·4 to 18·9)	−2·5 (−4·8 to −0·2)	−1·3 (−2·2 to −0·3)	−4·3 (−8·0 to −0·7)	−1·1 (−2·4 to 0·2)	0·2 (−4·4 to 4·8)	−1·6 (−3·4 to 0·3)
Mozambique	Low SDI	3·1 (2·5 to 3·8)	17·2 (14·5 to 20·1)	−1·5 (−2·7 to −0·2)	−0·5 (−1·5 to 0·5)	−0·5 (−2·3 to 1·2)	0·6 (−0·7 to 1·9)	−2·9 (−5·2 to −0·7)	−2·1 (−3·9 to −0·5)
Myanmar	Low-middle SDI	6·5 (5·0 to 8·4)	25·8 (23·5 to 28·4)	−1·3 (−2·9 to 0·4)	−1·6 (−2·1 to −1·0)	−0·3 (−2·6 to 2·3)	−1·7 (−2·5 to −1·0)	−2·7 (−5·7 to 0·3)	−1·3 (−2·4 to −0·2)
Namibia	Middle SDI	6·8 (5·3 to 8·6)	18·3 (16·5 to 20·1)	−1·8 (−3·0 to −0·5)	−1·1 (−1·7 to −0·6)	−1·2 (−3·1 to 0·8)	−1·0 (−1·8 to −0·1)	−2·8 (−5·4 to −0·3)	−1·4 (−2·6 to −0·3)
Nepal	Low-middle SDI	12·7 (9·6 to 16·0)	27·4 (23·9 to 31·4)	−2·5 (−3·9 to −0·9)	−1·7 (−2·4 to −1·0)	−0·9 (−2·7 to 1·3)	−1·1 (−1·9 to −0·2)	−4·8 (−7·7 to −2·2)	−2·6 (−4·0 to −1·1)
Netherlands	High SDI	16·6 (15·0 to 18·4)	19·0 (17·1 to 20·8)	−1·7 (−2·2 to −1·3)	−1·8 (−2·2 to −1·4)	−1·4 (−1·9 to −0·9)	−1·2 (−1·6 to −0·8)	−2·2 (−3·3 to −1·1)	−2·7 (−3·8 to −1·7)
New Zealand	High SDI	14·9 (14·0 to 15·9)	16·3 (15·3 to 17·2)	−1·8 (−2·1 to −1·5)	−1·5 (−1·8 to −1·3)	−1·3 (−1·7 to −0·9)	−1·2 (−1·5 to −0·8)	−2·5 (−3·2 to −1·7)	−2·1 (−2·8 to −1·5)
Nicaragua	Middle SDI	5·4 (3·9 to 7·2)	12·6 (10·0 to 15·7)	−0·6 (−2·4 to 1·1)	−0·9 (−2·1 to 0·4)	−1·0 (−3·6 to 1·6)	−1·3 (−3·1 to 0·6)	−0·2 (−3·9 to 3·4)	−0·3 (−2·8 to 2·5)
Niger	Low SDI	0·7 (0·4 to 1·0)	8·0 (6·6 to 9·5)	−1·9 (−4·4 to 0·6)	0·7 (−0·5 to 1·7)	−1·8 (−5·5 to 1·8)	0·2 (−1·3 to 2·0)	−1·9 (−6·5 to 2·9)	1·3 (−1·0 to 3·6)
Nigeria	Low-middle SDI	1·3 (0·9 to 1·9)	5·5 (4·6 to 6·7)	−4·4 (−6·3 to −2·4)	−3·2 (−4·1 to −2·4)	−7·1 (−10·2 to −3·9)	−3·9 (−5·2 to −2·7)	−0·3 (−4·9 to 4·4)	−2·1 (−4·3 to 0·1)
North Korea	Middle SDI	0·9 (0·6 to 1·4)	36·7 (33·6 to 39·8)	−0·8 (−3·4 to 1·9)	−0·7 (−1·2 to −0·3)	−0·6 (−4·9 to 3·9)	−0·5 (−1·1 to 0·1)	−1·0 (−7·0 to 5·1)	−1·1 (−2·1 to −0·1)
Northern Mariana Islands	High SDI	25·1 (21·1 to 29·6)	45·9 (41·7 to 50·1)	−0·2 (−1·1 to 0·7)	−0·3 (−0·7 to 0·2)	−0·1 (−1·6 to 1·3)	−0·2 (−0·9 to 0·5)	−0·3 (−2·1 to 1·5)	−0·4 (−1·4 to 0·7)
Norway	High SDI	14·8 (13·1 to 16·7)	15·0 (13·3 to 16·7)	−2·6 (−3·2 to −2·0)	−2·8 (−3·3 to −2·2)	−2·7 (−3·7 to −1·9)	−2·5 (−3·4 to −1·7)	−2·4 (−3·9 to −0·8)	−3·1 (−4·5 to −1·8)
Oman	High-middle SDI	1·5 (1·0 to 2·1)	9·5 (8·0 to 11·4)	0·6 (−1·6 to 2·9)	−1·4 (−2·4 to −0·5)	1·6 (−1·4 to 4·6)	−2·2 (−3·5 to −0·9)	−0·9 (−5·3 to 3·5)	−0·2 (−2·2 to 1·8)
Pakistan	Low-middle SDI	4·3 (3·4 to 5·5)	16·9 (14·9 to 19·2)	−0·4 (−2·3 to 1·6)	−2·0 (−2·8 to −1·1)	−0·7 (−3·9 to 2·9)	−1·1 (−2·3 to 0·3)	0·1 (−3·4 to 3·8)	−3·3 (−5·0 to −1·7)
Palestine	Middle SDI	2·5 (1·7 to 3·5)	30·4 (27·2 to 34·0)	−0·8 (−2·9 to 1·2)	−0·4 (−1·0 to 0·2)	−1·0 (−4·2 to 2·2)	−0·2 (−0·9 to 0·6)	−0·6 (−5·5 to 3·8)	−0·7 (−1·9 to 0·5)
Panama	High-middle SDI	2·4 (1·9 to 3·0)	4·6 (3·8 to 5·5)	−2·1 (−3·5 to −0·6)	−4·1 (−5·3 to −2·9)	−1·1 (−3·7 to 1·5)	−2·6 (−4·5 to −0·6)	−3·7 (−7·1 to −0·1)	−6·3 (−8·9 to −3·6)
Papua New Guinea	Low-middle SDI	15·0 (12·6 to 17·7)	37·6 (33·8 to 41·5)	−0·2 (−1·2 to 0·9)	−0·5 (−1·0 to 0·1)	1·2 (−0·2 to 2·6)	0·1 (−0·7 to 0·9)	−2·2 (−4·2 to −0·2)	−1·2 (−2·4 to −0·1)
Paraguay	Middle SDI	7·7 (5·8 to 10·2)	12·5 (10·1 to 15·5)	−0·4 (−2·3 to 1·3)	−2·1 (−3·3 to −1·0)	−1·3 (−3·9 to 1·6)	−1·6 (−3·2 to 0·2)	0·9 (−2·7 to 4·4)	−2·9 (−5·6 to −0·5)
Peru	High-middle SDI	4·2 (3·5 to 5·0)	11·9 (9·5 to 14·6)	−0·5 (−1·7 to 0·7)	−1·3 (−2·4 to −0·1)	−0·2 (−1·8 to 1·6)	−0·9 (−2·9 to 1·0)	−1·0 (−3·2 to 1·4)	−1·8 (−4·4 to 0·5)
Philippines	Middle SDI	7·4 (5·6 to 9·7)	34·5 (31·1 to 38·0)	−0·8 (−2·5 to 0·9)	−0·4 (−1·0 to 0·2)	−1·0 (−3·4 to 1·5)	−0·2 (−0·9 to 0·6)	−0·6 (−3·7 to 2·6)	−0·8 (−2·0 to 0·3)
Poland	High SDI	19·3 (16·7 to 22·1)	26·7 (24·6 to 28·8)	−0·9 (−1·6 to −0·3)	−1·7 (−2·1 to −1·3)	−0·9 (−1·7 to 0·0)	−1·8 (−2·3 to −1·3)	−0·9 (−2·7 to 0·9)	−1·5 (−2·5 to −0·4)
Portugal	High-middle SDI	12·7 (11·0 to 14·8)	24·9 (22·7 to 27·2)	1·3 (0·4 to 2·1)	−1·0 (−1·4 to −0·6)	2·1 (0·9 to 3·3)	−1·2 (−1·8 to −0·6)	0·0 (−1·8 to 1·8)	−0·7 (−1·7 to 0·3)
Puerto Rico	High SDI	5·7 (4·4 to 7·4)	12·1 (10·1 to 14·5)	−0·3 (−1·8 to 1·2)	−0·4 (−1·5 to 0·8)	−0·1 (−2·6 to 2·7)	−0·2 (−2·0 to 1·6)	−0·7 (−4·3 to 2·9)	−0·5 (−3·1 to 2·0)
Qatar	High-middle SDI	2·3 (1·7 to 3·1)	12·2 (10·4 to 14·0)	−3·2 (−5·2 to −1·4)	−0·1 (−1·1 to 0·8)	−6·0 (−9·8 to −2·6)	−1·6 (−3·1 to 0·1)	1·0 (−3·7 to 5·8)	2·0 (−0·2 to 4·2)
Romania	High-middle SDI	15·7 (13·3 to 18·4)	29·3 (26·9 to 31·9)	0·0 (−0·9 to 1·1)	−0·1 (−0·5 to 0·5)	0·8 (−0·4 to 2·1)	0·9 (0·3 to 1·7)	−1·1 (−3·1 to 1·1)	−1·6 (−2·6 to −0·6)
Russia	High SDI	12·3 (10·6 to 14·2)	38·2 (36·0 to 40·3)	1·8 (0·9 to 2·7)	−0·5 (−0·8 to −0·2)	3·2 (2·0 to 4·4)	0·2 (−0·2 to 0·6)	−0·3 (−2·0 to 1·5)	−1·5 (−2·1 to −0·9)
Rwanda	Low SDI	3·8 (3·2 to 4·6)	12·4 (10·6 to 14·3)	0·1 (−1·0 to 1·3)	−1·2 (−2·2 to −0·3)	−0·3 (−2·2 to 1·5)	−1·3 (−2·8 to 0·2)	0·6 (−1·8 to 3·0)	−1·1 (−3·1 to 0·9)
Saint Lucia	High-middle SDI	1·8 (1·3 to 2·4)	14·3 (11·5 to 17·7)	0·0 (−2·0 to 1·8)	0·6 (−0·6 to 1·9)	0·4 (−2·6 to 3·2)	1·5 (−0·5 to 3·5)	−0·7 (−4·8 to 3·2)	−0·7 (−3·4 to 1·8)
Saint Vincent and the Grenadines	High-middle SDI	1·8 (1·3 to 2·5)	10·8 (8·6 to 13·3)	−0·8 (−2·5 to 0·9)	−1·6 (−2·8 to −0·5)	−0·7 (−3·6 to 2·2)	−2·1 (−4·2 to −0·2)	−1·0 (−5·0 to 3·0)	−0·9 (−3·6 to 1·8)
Samoa	Middle SDI	11·9 (9·7 to 14·4)	34·8 (30·8 to 38·9)	−0·6 (−1·7 to 0·5)	−1·0 (−1·6 to −0·4)	−0·5 (−2·1 to 1·2)	−1·1 (−1·8 to −0·3)	−0·8 (−3·1 to 1·4)	−0·9 (−2·2 to 0·3)
São Tomé and Príncipe	Low-middle SDI	1·0 (0·7 to 1·5)	6·2 (5·0 to 7·3)	−1·0 (−3·2 to 1·3)	−0·2 (−1·3 to 0·9)	−1·4 (−5·0 to 2·1)	−0·5 (−2·2 to 1·1)	−0·2 (−5·0 to 4·6)	0·2 (−2·1 to 2·5)
Saudi Arabia	High-middle SDI	1·7 (1·4 to 2·0)	19·5 (18·5 to 20·6)	−2·9 (−4·0 to −1·9)	2·4 (2·1 to 2·8)	−4·9 (−6·5 to −3·2)	3·6 (2·9 to 4·2)	0·0 (−2·2 to 2·2)	0·7 (0·0 to 1·6)
Senegal	Low SDI	1·5 (1·1 to 1·9)	8·3 (7·2 to 9·6)	1·1 (−0·8 to 3·0)	−3·0 (−3·9 to −2·1)	−0·9 (−4·3 to 2·7)	−2·4 (−3·8 to −0·9)	4·1 (−0·1 to 8·3)	−3·9 (−5·8 to −2·0)
Serbia	High-middle SDI	18·9 (15·6 to 22·4)	28·7 (25·9 to 31·6)	0·2 (−0·8 to 1·2)	0·1 (−0·5 to 0·7)	1·3 (−0·1 to 2·8)	1·1 (0·4 to 1·8)	−1·5 (−3·5 to 0·3)	−1·5 (−2·6 to −0·5)
Seychelles	High-middle SDI	4·2 (2·8 to 5·9)	23·7 (20·7 to 26·7)	0·6 (−1·7 to 2·7)	−0·2 (−1·0 to 0·5)	0·0 (−3·3 to 3·3)	−0·3 (−1·4 to 0·9)	1·4 (−3·2 to 5·6)	−0·2 (−1·6 to 1·2)
Sierra Leone	Low SDI	3·8 (2·7 to 5·2)	21·7 (19·4 to 24·3)	−0·9 (−3·0 to 1·2)	−0·5 (−1·3 to 0·2)	−0·4 (−3·5 to 2·7)	0·0 (−1·1 to 1·2)	−1·7 (−5·9 to 2·8)	−1·3 (−2·8 to 0·3)
Singapore	High SDI	6·3 (5·3 to 7·4)	17·9 (16·2 to 19·4)	0·3 (−0·6 to 1·2)	−0·9 (−1·4 to −0·4)	−0·4 (−1·7 to 0·9)	−1·6 (−2·3 to −0·9)	1·3 (−0·5 to 3·0)	0·2 (−0·8 to 1·1)
Slovakia	High SDI	15·1 (12·5 to 18·0)	25·6 (23·1 to 28·1)	−0·2 (−1·3 to 1·0)	−1·4 (−2·0 to −0·9)	−0·5 (−2·2 to 1·4)	−2·0 (−2·8 to −1·1)	0·3 (−2·1 to 2·5)	−0·5 (−1·7 to 0·6)
Slovenia	High SDI	18·5 (15·8 to 21·6)	23·1 (20·8 to 25·5)	−0·4 (−1·4 to 0·6)	−1·7 (−2·3 to −1·2)	−0·7 (−2·2 to 0·8)	−2·7 (−3·4 to −2·0)	0·0 (−1·8 to 1·9)	−0·3 (−1·6 to 0·9)
Solomon Islands	Low-middle SDI	9·7 (7·9 to 11·8)	28·5 (24·8 to 32·3)	−0·5 (−1·7 to 0·6)	−0·4 (−1·1 to 0·4)	−0·2 (−1·9 to 1·6)	−0·2 (−1·3 to 0·9)	−1·0 (−3·3 to 1·4)	−0·6 (−2·0 to 0·8)
Somalia	Low SDI	1·6 (1·3 to 2·0)	13·1 (10·7 to 16·0)	−2·1 (−3·3 to −0·9)	−1·1 (−2·2 to 0·0)	−2·6 (−4·5 to −0·6)	−1·2 (−2·9 to 0·5)	−1·3 (−3·9 to 1·2)	−0·9 (−3·2 to 1·6)
South Africa	Middle SDI	7·5 (7·0 to 8·1)	21·9 (21·2 to 22·7)	−2·9 (−3·4 to −2·4)	−1·9 (−2·1 to −1·7)	−4·1 (−4·9 to −3·3)	−2·8 (−3·1 to −2·5)	−1·0 (−2·1 to 0·1)	−0·6 (−1·0 to −0·1)
South Korea	High SDI	8·8 (7·6 to 10·1)	33·5 (31·6 to 35·5)	2·4 (1·5 to 3·2)	−2·3 (−2·6 to −2·1)	3·6 (2·3 to 4·8)	−2·8 (−3·1 to −2·4)	0·6 (−1·3 to 2·3)	−1·6 (−2·4 to −1·0)
South Sudan	Low SDI	1·7 (1·3 to 2·0)	13·3 (10·9 to 16·0)	−2·1 (−3·2 to −0·9)	−1·0 (−2·1 to 0·0)	−2·5 (−4·4 to −0·7)	−1·1 (−2·7 to 0·6)	−1·4 (−3·6 to 1·0)	−1·0 (−3·4 to 1·4)
Spain	High-middle SDI	18·6 (16·4 to 20·7)	25·6 (23·3 to 27·8)	−1·1 (−1·6 to −0·6)	−1·7 (−2·1 to −1·3)	−0·6 (−1·2 to 0·2)	−1·5 (−1·9 to −1·0)	−1·9 (−3·3 to −0·6)	−2·0 (−3·1 to −1·0)
Sri Lanka	High-middle SDI	1·2 (0·8 to 1·7)	19·2 (16·8 to 21·5)	−3·6 (−5·8 to −1·5)	−0·6 (−1·4 to 0·1)	−5·9 (−9·1 to −2·7)	−1·0 (−1·9 to 0·1)	−0·2 (−4·3 to 4·1)	−0·1 (−1·6 to 1·2)
Sudan	Low-middle SDI	0·4 (0·2 to 0·6)	1·3 (1·0 to 1·7)	1·0 (−2·0 to 4·4)	−0·5 (−2·1 to 1·0)	0·4 (−4·7 to 5·7)	−1·1 (−3·4 to 1·3)	1·9 (−4·8 to 8·8)	0·3 (−2·7 to 3·6)
Suriname	High-middle SDI	7·5 (5·5 to 9·9)	27·3 (24·5 to 30·2)	0·4 (−1·2 to 2·1)	1·2 (0·3 to 2·2)	0·7 (−1·9 to 3·0)	0·8 (−0·7 to 2·5)	−0·1 (−3·7 to 3·6)	1·8 (−0·1 to 3·8)
Swaziland	Middle SDI	1·3 (1·0 to 1·7)	10·2 (9·0 to 11·4)	−3·1 (−4·6 to −1·6)	−2·2 (−2·9 to −1·5)	−2·7 (−5·2 to −0·6)	−3·3 (−4·4 to −2·4)	−3·7 (−7·0 to −0·5)	−0·6 (−1·9 to 0·9)
Sweden	High SDI	11·4 (10·6 to 12·1)	10·3 (9·7 to 11·0)	−2·7 (−3·2 to −2·2)	−3·2 (−3·6 to −2·8)	−2·5 (−3·1 to −1·8)	−4·4 (−5·0 to −3·8)	−3·1 (−3·9 to −2·3)	−1·4 (−2·1 to −0·6)
Switzerland	High SDI	16·5 (14·6 to 18·7)	21·9 (19·6 to 24·1)	−1·4 (−2·0 to −0·9)	−1·3 (−1·8 to −0·9)	−1·2 (−1·9 to −0·5)	−1·0 (−1·7 to −0·5)	−1·8 (−3·3 to −0·4)	−1·6 (−2·9 to −0·4)
Syria	Middle SDI	8·5 (5·9 to 11·6)	21·0 (18·0 to 24·2)	−1·2 (−3·1 to 0·6)	−0·8 (−1·6 to 0·1)	−1·1 (−4·0 to 1·9)	−0·6 (−1·9 to 0·7)	−1·4 (−5·5 to 2·5)	−1·0 (−2·6 to 0·6)
Taiwan	High SDI	3·4 (2·2 to 5·2)	19·0 (16·8 to 21·0)	−1·1 (−3·5 to 1·3)	−3·0 (−3·5 to −2·4)	−1·4 (−5·1 to 2·5)	−2·0 (−2·8 to −1·3)	−0·7 (−5·6 to 3·8)	−4·3 (−5·7 to −3·2)
Tajikistan	Middle SDI	0·4 (0·3 to 0·6)	19·6 (16·8 to 22·7)	−2·9 (−4·7 to −1·0)	−2·8 (−3·6 to −2·2)	−4·1 (−7·0 to −1·3)	−4·2 (−5·2 to −3·1)	−1·0 (−4·9 to 2·9)	−0·9 (−2·6 to 0·9)
Tanzania	Low-middle SDI	1·4 (1·2 to 1·8)	16·0 (13·9 to 18·4)	−0·7 (−1·9 to 0·5)	−0·5 (−1·4 to 0·4)	−1·0 (−3·0 to 1·0)	−0·3 (−1·6 to 1·3)	−0·4 (−3·0 to 2·4)	−0·9 (−2·7 to 0·9)
Thailand	High-middle SDI	3·3 (2·3 to 4·6)	30·9 (28·0 to 34·1)	−1·6 (−3·6 to 0·4)	−1·3 (−1·7 to −0·9)	−2·7 (−5·6 to 0·3)	−1·9 (−2·4 to −1·3)	−0·1 (−4·3 to 3·9)	−0·5 (−1·6 to 0·6)
The Bahamas	High SDI	3·7 (2·7 to 5·1)	8·0 (6·3 to 10·1)	3·0 (1·2 to 4·8)	2·0 (0·5 to 3·3)	2·7 (−0·1 to 5·6)	1·4 (−1·0 to 3·7)	3·3 (−0·6 to 7·1)	2·8 (−0·1 to 5·6)
The Gambia	Low SDI	0·8 (0·5 to 1·2)	19·3 (16·7 to 21·9)	−1·6 (−3·8 to 0·6)	−0·8 (−1·5 to 0·1)	−2·0 (−5·6 to 1·5)	−0·7 (−2·0 to 0·6)	−1·0 (−5·8 to 3·9)	−0·8 (−2·7 to 1·1)
Timor-Leste	Low-middle SDI	12·4 (9·8 to 15·1)	39·8 (37·2 to 42·5)	4·5 (2·8 to 6·3)	−0·1 (−0·5 to 0·4)	3·7 (0·3 to 7·0)	0·2 (−0·5 to 0·9)	5·8 (2·3 to 9·5)	−0·4 (−1·2 to 0·6)
Togo	Low SDI	1·1 (0·7 to 1·6)	9·2 (7·9 to 10·7)	−2·3 (−4·5 to 0·0)	−0·2 (−1·1 to 0·7)	−2·3 (−5·8 to 1·0)	0·0 (−1·6 to 1·5)	−2·4 (−7·2 to 2·9)	−0·5 (−2·6 to 1·7)
Tonga	Middle SDI	11·0 (9·2 to 12·9)	38·3 (35·2 to 41·8)	−1·2 (−2·2 to −0·3)	0·1 (−0·4 to 0·6)	−2·1 (−3·5 to −0·9)	0·2 (−0·4 to 0·9)	0·1 (−1·7 to 2·0)	−0·2 (−1·2 to 0·8)
Trinidad and Tobago	High SDI	5·1 (3·8 to 6·5)	22·3 (19·0 to 25·8)	1·3 (−0·4 to 3·1)	−0·5 (−1·4 to 0·4)	1·4 (−1·7 to 4·5)	−0·8 (−2·2 to 0·7)	1·2 (−2·7 to 4·9)	−0·1 (−2·1 to 1·8)
Tunisia	Middle SDI	3·0 (2·0 to 4·2)	36·1 (32·0 to 40·4)	−2·4 (−4·5 to −0·4)	−0·5 (−1·1 to 0·1)	−3·9 (−6·7 to −1·0)	−0·2 (−1·1 to 0·6)	−0·1 (−4·1 to 3·8)	−0·9 (−2·1 to 0·4)
Turkey	High-middle SDI	13·7 (11·0 to 16·7)	31·2 (28·6 to 33·9)	0·1 (−1·0 to 1·5)	−1·9 (−2·3 to −1·4)	0·3 (−1·4 to 2·3)	−1·4 (−2·0 to −0·8)	−0·3 (−2·6 to 2·1)	−2·6 (−3·6 to −1·6)
Turkmenistan	High-middle SDI	0·9 (0·7 to 1·3)	13·3 (11·4 to 15·5)	−0·6 (−2·4 to 1·3)	−1·2 (−2·1 to −0·3)	−0·8 (−3·8 to 2·0)	−1·5 (−3·0 to −0·1)	−0·2 (−4·0 to 3·9)	−0·8 (−2·8 to 1·0)
Uganda	Low SDI	2·6 (2·2 to 3·1)	9·3 (8·0 to 10·8)	−0·1 (−1·2 to 1·1)	−2·2 (−3·2 to −1·1)	−0·9 (−2·8 to 0·9)	−1·5 (−3·2 to 0·2)	1·2 (−1·3 to 3·7)	−3·2 (−5·5 to −1·1)
Ukraine	High-middle SDI	11·5 (9·4 to 13·7)	40·6 (37·8 to 43·4)	0·2 (−1·0 to 1·3)	−0·8 (−1·2 to −0·5)	0·7 (−1·0 to 2·4)	−0·2 (−0·6 to 0·3)	−0·7 (−2·8 to 1·4)	−1·8 (−2·5 to −1·1)
United Arab Emirates	High SDI	1·8 (1·2 to 2·6)	11·3 (9·3 to 13·4)	−2·3 (−4·5 to −0·2)	−0·8 (−1·8 to 0·2)	−4·0 (−7·3 to −0·6)	−0·3 (−1·8 to 1·1)	0·1 (−4·5 to 4·8)	−1·6 (−3·7 to 0·5)
UK	High SDI	18·1 (16·4 to 20·0)	19·9 (18·1 to 21·7)	−1·4 (−2·0 to −0·8)	−1·4 (−1·9 to −1·0)	−1·6 (−2·3 to −0·8)	−1·8 (−2·4 to −1·1)	−1·2 (−2·3 to −0·1)	−0·9 (−2·0 to 0·1)
USA	High SDI	11·7 (11·5 to 12·0)	14·4 (14·0 to 14·7)	−2·2 (−2·3 to −2·1)	−2·0 (−2·1 to −1·8)	−2·4 (−2·5 to −2·3)	−1·7 (−1·8 to −1·6)	−2·0 (−2·2 to −1·8)	−2·4 (−2·6 to −2·1)
Uruguay	High-middle SDI	17·0 (14·8 to 19·3)	21·3 (18·8 to 24·0)	−1·0 (−1·7 to −0·3)	−2·0 (−2·6 to −1·4)	−0·4 (−1·4 to 0·6)	−1·9 (−2·6 to −1·1)	−1·8 (−3·4 to −0·3)	−2·3 (−3·6 to −1·0)
Uzbekistan	High-middle SDI	3·3 (2·5 to 4·2)	14·1 (12·4 to 16·0)	2·4 (0·9 to 4·1)	−0·6 (−1·4 to 0·3)	3·6 (1·0 to 6·2)	0·3 (−1·0 to 1·6)	0·6 (−2·5 to 3·8)	−1·9 (−3·6 to −0·2)
Vanuatu	Low-middle SDI	2·8 (2·2 to 3·4)	28·5 (25·5 to 31·7)	−1·8 (−3·0 to −0·5)	−0·7 (−1·3 to −0·1)	−2·9 (−4·8 to −1·0)	−2·1 (−3·2 to −1·1)	−0·2 (−3·0 to 2·5)	1·4 (0·0 to 2·9)
Venezuela	High-middle SDI	9·9 (7·5 to 12·7)	16·7 (13·8 to 20·0)	−0·6 (−2·1 to 0·9)	−0·7 (−1·7 to 0·4)	−0·2 (−2·7 to 2·2)	−0·5 (−2·2 to 1·2)	−1·1 (−4·5 to 2·1)	−0·9 (−3·2 to 1·5)
Vietnam	Middle SDI	1·4 (1·0 to 1·9)	35·4 (32·1 to 38·7)	−4·4 (−6·1 to −2·7)	−1·5 (−1·9 to −1·0)	−7·5 (−9·6 to −5·2)	−1·8 (−2·3 to −1·2)	0·3 (−3·7 to 4·2)	−1·0 (−2·1 to 0·0)
Virgin Islands, USA	High SDI	2·8 (2·1 to 3·8)	4·4 (3·4 to 5·6)	−1·2 (−2·9 to 0·6)	−1·2 (−2·7 to 0·2)	−1·0 (−3·9 to 1·8)	−1·0 (−3·2 to 1·2)	−1·4 (−5·0 to 2·1)	−1·5 (−4·7 to 1·5)
Yemen	Low-middle SDI	6·3 (4·3 to 8·8)	18·8 (16·1 to 21·8)	−0·9 (−3·1 to 1·1)	−0·7 (−1·6 to 0·2)	−1·0 (−3·7 to 2·0)	−0·3 (−1·4 to 0·9)	−0·8 (−4·8 to 3·2)	−1·4 (−3·0 to 0·2)
Zambia	Low-middle SDI	3·1 (2·5 to 3·8)	15·2 (12·3 to 18·1)	−0·5 (−1·7 to 0·7)	−0·7 (−1·8 to 0·2)	−0·4 (−2·1 to 1·5)	−0·7 (−2·1 to 0·7)	−0·8 (−3·3 to 1·8)	−0·8 (−2·8 to 1·1)
Zimbabwe	Low-middle SDI	1·4 (1·0 to 1·9)	20·8 (18·9 to 22·7)	−2·8 (−4·6 to −1·1)	−0·3 (−0·8 to 0·3)	−1·5 (−4·1 to 1·0)	−0·5 (−1·4 to 0·3)	−4·9 (−8·3 to −1·6)	0·1 (−0·9 to 1·2)

Data in parentheses are 95% uncertainty intervals. SDI=Socio-demographic Index.

Between 1990 and 2015, the global age-standardised prevalence of daily smoking fell significantly for both sexes, decreasing by 28·4% (95% UI 25·8–31·1) for men and 34·4% (29·4–38·6) for women (table 2). 13 countries (Australia, Brazil, China, Denmark, Dominican Republic, Iceland, Kenya, the Netherlands, New Zealand, Norway, Sweden, Switzerland, and the USA) recorded significant annualised rates of decline both between 1990 and 2005 and 2005 and 2015, suggesting sustained progress in tobacco control (table 1). 18 countries showed a faster annualised rate of reduction in daily smoking in the most recent decade compared with between 1990 and 2005. Focusing on the most recent decade, since 2005, 53 (27%) of 195 countries and territories recorded significant decreases in age-standardised prevalence of male daily smoking, whereas only 32 (16%) recorded significant reductions for women.

**Table 2:** Size of the smoking population, prevalence, and 1990–2015 percent change in prevalence, by sex, for the ten countries with the largest smoking populations and worldwide

	**Overall rank**	**Ages 15–19 rank**	**2015 female smoking population (millions)**	**2015 male smoking population (millions)**	**2015 female age-standardised prevalence**	**2015 male age-standardised prevalence**	**2015 female prevalence ages 15–19**	**2015 male prevalence ages 15–19**	**Female age-standardised percent change 1990–2015**	**Male age-standardised percent change 1990–2015**	**Female ages 15–19 percent change 1990–2015**	**Male ages 15–19 percent change 1990–2015**
China	1	1	14·4 (10·5 to 19·5)	253·9 (241·2 to 266·6)	2·2 (2·1 to 2·4)	37·5 (36·9 to 38·0)	1·0 (0·7 to 1·4)	17·9 (16·4 to 19·6)	−48·4 (−55·1 to −41·2)	−22·4 (−24·0 to −20·7)	−9·7 (−47·0 to 49·6)	−3·6 (−15·4 to 10·1)
India	2	2	13·5 (9·4 to 19·7)	90·8 (78·9 to 104·3)	2·9 (2·6 to 3·2)	17·5 (16·8 to 18·2)	1·4 (1·0 to 2·0)	6·0 (5·1 to 7·0)	−7·1 (−22·3 to 9·1)	−40·6 (−44·3 to −36·6)	56·1 (−9·8 to 175·7)	−17·1 (−35·2 to 8·0)
Indonesia	3	3	3·9 (1·1 to 9·8)	49·8 (38·9 to 60·5)	3·8 (2·7 to 5·1)	46·7 (43·9 to 49·5)	2·3 (0·6 to 6·1)	27·7 (18·4 to 38·3)	57·7 (0·8 to 154·2)	6·3 (−3·4 to 17·0)	139·6 (−51·4 to 1468·0)	9·1 (−36·1 to 85·0)
USA	4	4	17·2 (16·0 to 18·4)	20·4 (18·9 to 22·0)	11·7 (11·5 to 12·0)	14·4 (14·0 to 14·7)	7·0 (6·4 to 7·6)	7·7 (7·1 to 8·4)	−43·0 (−44·3 to −41·6)	−38·6 (−40·3 to −37·0)	−62·7 (−66·1 to −58·6)	−59·7 (−63·8 to −55·1)
Russia	5	9	8·3 (5·0 to 12·6)	24·9 (20·3 to 29·6)	12·3 (10·6 to 14·2)	38·2 (36·0 to 40·3)	11·8 (6·6 to 19·5)	22·4 (16·4 to 30·0)	56·2 (25·1 to 96·3)	−11·2 (−17·3 to −4·8)	38·0 (−40·3 to 224·9)	−30·6 (−51·7 to −3·6)
Bangladesh	6	6	1·0 (0·2 to 3·0)	24·1 (15·3 to 33·6)	1·8 (1·1 to 2·6)	38·0 (34·1 to 42·6)	0·9 (0·1 to 2·7)	14·9 (7·1 to 26·2)	−51·4 (−72·6 to −10·6)	6·8 (−9·9 to 27·4)	−37·9 (−91·1 to 390·2)	−7·6 (−63·6 to 148·3)
Japan	7	16	4·9 (4·4 to 5·6)	15·3 (14·4 to 16·3)	9·3 (8·9 to 9·6)	26·7 (26·1 to 27·1)	6·7 (5·9 to 7·5)	11·5 (10·7 to 12·4)	−16·0 (−20·2 to −11·6)	−44·8 (−46·0 to −43·5)	−15·1 (−27·8 to 1·3)	−62·0 (−65·4 to −58·0)
Brazil	8	8	7·7 (5·7 to 10·2)	11·1 (8·9 to 13·9)	8·2 (7·5 to 9·0)	12·6 (11·8 to 13·5)	4·8 (3·3 to 7·0)	7·4 (5·6 to 9·6)	−55·8 (−61·9 to −48·7)	−56·5 (−61·1 to −51·9)	−67·3 (−81·4 to −42·8)	−60·8 (−74·0 to −40·4)
Germany	9	13	7·1 (4·8 to 9·9)	9·2 (6·6 to 12·1)	19·4 (17·3 to 21·7)	25·2 (22·8 to 27·4)	15·9 (10·1 to 23·4)	18·8 (12·6 to 26·6)	−7·8 (−19·2 to 6·2)	−20·6 (−28·9 to −12·2)	−22·6 (−54·6 to 27·6)	−23·7 (−51·5 to 16·7)
Philippines	10	10	2·6 (0·9 to 5·8)	13·2 (9·1 to 17·7)	7·4 (5·6 to 9·7)	34·5 (31·1 to 38·0)	2·5 (0·7 to 6·4)	16·0 (9·1 to 25·5)	−18·2 (−46·6 to 24·1)	−10·1 (−22·0 to 5·0)	−11·3 (−83·9 to 387·4)	−3·2 (−55·0 to 112·4)
Global			165·0 (141·2 to 202·1)	768·1 (690·1 to 852·2)	5·4 (5·2 to 5·7)	25·0 (24·2 to 25·7)	3·0 (2·6 to 3·7)	10·6 (9·3 to 12·1)	−34·4 (−38·6 to −29·4)	−28·4 (−31·1 to −25·8)	−36·8 (−49·7 to −20·7)	−34·3 (−45·3 to −21·1)

Age-standardised estimates and estimates for the 15–19 age group are reported. 95% uncertainty intervals are reported in parentheses. Overall rank is calculated based on the size of the smoking population, both sexes and all ages (10 years and older) combined. Ages 15–19 years rank is calculated based on the size of the smoking population aged 15–19 years, both sexes combined.

### Countries with large smoking populations

In 2015, there were 933·1 million (95% UI 831·3–1054·3) daily smokers in the world, 82·3% of whom were men (768·1 million [690·1–852·2]). The ten countries with the largest number of smokers together accounted for 63·6% of the world's daily smokers. China, India, and Indonesia, the three leading countries in total number of male smokers, accounted for 51·4% of the world's male smokers in 2015. On the other hand, the USA, China, and India, which were the leading three countries in total number of female smokers, accounted for only 27·3% of the world's female smokers. Together, these results suggest that the tobacco epidemic is less geographically concentrated for women than for men.

Among the ten countries with the largest number of total smokers in 2015, seven recorded significant decreases in male smoking prevalence and five had significant decreases in female smoking prevalence since 1990 (table 2). Of these countries, Brazil recorded the largest overall reduction in prevalence for both male and female daily smoking, which dropped by 56·5% (51·9–61·1) and 55·8% (48·7–61·9), respectively, between 1990 and 2015. Indonesia, Bangladesh, and the Philippines did not have significant reductions in male prevalence of daily smoking since 1990, and the Philippines, Germany, and India had no significant decreases in smoking among women. All of the three countries with female age-standardised smoking prevalence less than 3·0% (China, India, and Bangladesh) succeeded in keeping smoking prevalence low in women. Notably, female prevalence of daily smoking significantly increased in Russia and Indonesia since 1990 (table 2).

### Adolescents

Delving into the smoking patterns of adolescents can shed light on trends in smoking initiation.^47^ Between 1990 and 2015, the global prevalence of daily smoking for this age group significantly decreased for each sex, falling from 16·1% (95% UI 14·4–18·0) to 10·6% (9·3–12·1) for men and from 4·8% (4·3–5·6) to 3·0% (2·6–3·7) for women (table 2). Despite global decreases, several countries still had a high prevalence of smoking among individuals aged between 15 and 19 years. In 2015, there were 22 countries with female smoking prevalence in this age group higher than 15·0%, 18 of which were located in western or central Europe. Countries with high male smoking prevalence were much more dispersed. Of the 24 countries with male smoking prevalence higher than 20·0%, six were in eastern Europe, and the remainder were spread across ten other regions (appendix pp 13, 14). The rank of countries with the largest smoking populations for the 15–19 years age group was mostly consistent with the rank for all-age smoking populations (table 2).

Although no country had a significant increase for men or women in this age group since 2005, only three countries saw smoking prevalence in 15 to 19 year-olds significantly drop for both men and women since 2005 (New Zealand, Iceland, and the USA). Iceland had the largest significant decrease among men, decreasing from 14·8% (95% UI 11·7–18·5) in 2005 to 9·0% (5·6–13·3) in 2015. New Zealand had the largest significant decline among women, decreasing from 20·8% (18·1–23·8) in 2005 to 12·5% (10·1–15·5) in 2015 (available to view through GHDx).

### Shifts in patterns of smoking across cohorts

Parsing out daily smoking prevalence by age group and birth cohort allows for a more fine-grained examination of smoking prevalence, age patterns, and temporal trends by level of development (figure 2). Male age patterns of smoking were fairly consistent across levels of SDI, with prevalence generally peaking between the ages of 25 and 35 years. For women, however, age patterns varied more by SDI; female smoking prevalence typically peaked around age 25 years for high and high-middle SDI countries, while prevalence generally increased until age 60 years in low to middle SDI countries. Across birth cohorts, smoking prevalence decreased by age group, sex, and SDI level. The most notable decreases were recorded in high and high-middle SDI countries for men, where sizeable reductions in smoking prevalence in 15 to 24 year-olds occurred across birth cohorts. Middle SDI countries, which have the highest levels of daily smoking among men, had minimal changes in prevalence across birth cohorts, suggesting far less progress in curbing smoking initiation or promoting cessation. For women, prevalence is consistently lower than in men; nevertheless, reductions in smoking prevalence across birth cohorts generally were smaller than those recorded for men.

### Deaths and disease burden attributable to smoking

**Figure 3:**
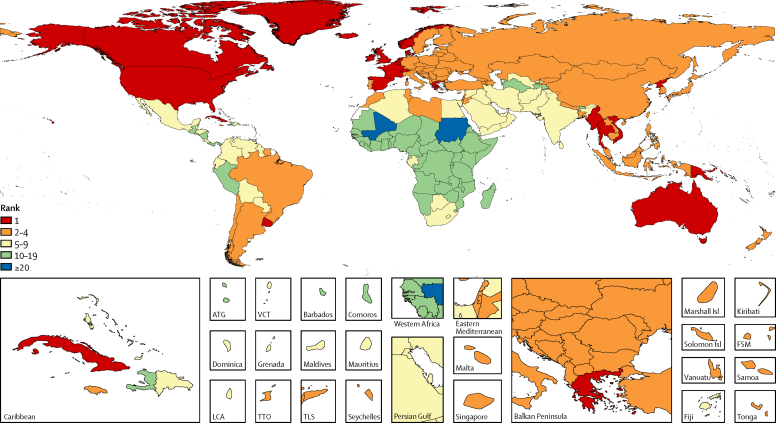
Rankings of smoking as a risk factor for all-cause, all-age attributable DALYs for both sexes combined in 2015 DALYs=disability-adjusted life-years. ATG=Antigua and Barbuda. VCT=Saint Vincent and the Grenadines. LCA=Saint Lucia. TTO=Trinidad and Tobago. TLS=Timor-Leste. FSM=Federated States of Micronesia.

In 2015, 6·4 million deaths (95% UI 5·7–7·0) were attributable to smoking worldwide, representing a 4·7% (1·2–8·5) increase in smoking-attributable deaths since 2005. More than 75% of these deaths were in men, and 52·2% took place in four countries (China, India, the USA, and Russia). Smoking was the second-leading risk factor for attributable mortality among both sexes in both 2005 and 2015, following high-systolic blood pressure.^1^ The relative ranking of smoking-attributable disease burden, as measured in DALYs, increased from third to second between 2005 and 2015. In 2015, there were 148·6 million (95% UI 134·2–163·1) smoking-attributable DALYs worldwide, and smoking was the leading risk factor for attributable disease burden in 24 countries, an increase from 16 countries in 1990 (figure 3). Further, smoking was ranked among the leading five risk factors for 109 countries in 2015. Between 2005 and 2015, only Egypt recorded a significant increase in the age-standardised smoking-attributable mortality rate among both sexes, increasing by 11·4% (95% UI 0·3–24·7) over that time period. On the other hand, 82 countries had significant decreases in their age-standardised smoking-attributable mortality rates since 2005.

Overall, in 2015, cardiovascular diseases (41·2%), cancers (27·6%), and chronic respiratory diseases (20·5%) were the three leading causes of smoking-attributable age-standardised DALYs for both sexes. Of all risk factors, smoking was the leading risk factor for cancers and chronic respiratory diseases, but only the ninth leading risk factor for cardiovascular diseases.^1^ The appendix shows the 30 leading causes of DALYs attributable to smoking, including changes over time (pp 19, 20). For women, the leading cause of smoking-attributable DALYs was COPD, whereas the leading cause for men was ischaemic heart disease.

### Decomposing changes in attributable burden due to smoking

**Figure 4:**
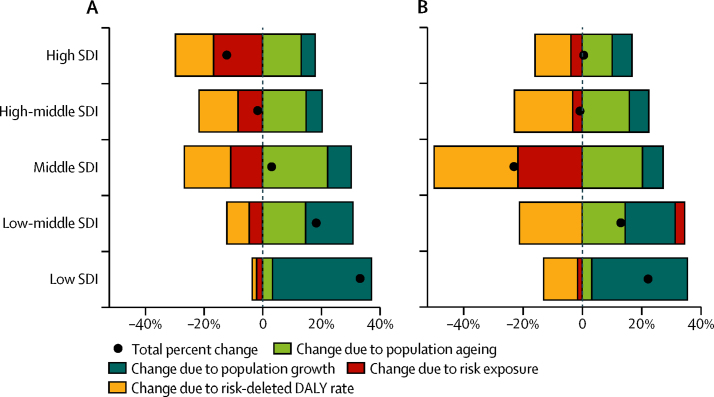
Decomposition of changes in all-cause DALYs attributable to smoking from 2005 to 2015, by SDI, for men (A) and women (B) Changes due to population growth, population ageing, risk exposure (smoking prevalence), and the risk-deleted DALY rate are shown. Locations are reported in order of the number of attributable DALYs for both sexes in 2015. DALYs=disability-adjusted life-years. SDI=Socio-demographic Index.

Relative to changes in smoking exposure, the main drivers of overall changes in attributable burden due to smoking varied by both sex and SDI level (figure 4). Since 2005, all-cause DALYs attributable to smoking for men decreased by 11·8% (95% UI 10·0–13·9) in high-SDI countries, the only SDI level with a significant decrease in attributable burden for men. For women, only middle-SDI countries had a significant reduction in all-cause DALYs attributable to smoking (a 22·6% decrease [9·0–32·8]) between 2005 and 2015. In both instances, a combination of reduced smoking exposure and reduced risk-deleted DALY rates contributed to overall reductions. Conversely, all-cause burden due to smoking significantly increased in low SDI and low-middle SDI countries since 2005 for men. This rise in attributable DALYs was driven mainly by a combination of population growth and population ageing for both sexes. In women, while rising exposure to smoking has resulted in increased DALYs due to smoking for low-middle SDI countries, this increase was not significant. Generally, population growth was the leading factor for increasing attributable burden due to smoking among the low SDI countries between 2005 and 2015. For countries of middle to high SDI, more pronounced sex differences emerged. For instance, decreases in male smoking prevalence propelled an overall reduction in attributable burden for high SDI countries, whereas changes in smoking exposure had minimal effects on overall burden for women at similarly high levels of SDI.

A complete dataset of all results by geography, year, sex, and age group can be downloaded through GHDx, and an interactive data visualisation of smoking prevalence results can be found online.

## Discussion

Despite more than half a century of unequivocal evidence of the harmful effects of tobacco on health,^48^, ^49^ in 2015, one in every four men in the world was a daily smoker. Prevalence has been, and remains, significantly lower in women—roughly one in every 20 women smoked daily in 2015. Nonetheless, much progress has been accomplished in the past 25 years. Specifically, the age-standardised global prevalence of daily smoking fell to 15·3% (95% UI 14·8–15·9), a 29·4% (27·1–31·8) reduction from 1990, with smoking rates decreasing from 34·9% (34·1–35·7) to 25·0% (24·2–25·7) in men and from 8·2% (7·9–8·6) to 5·4% (5·1–5·7) in women. These reductions were especially pronounced in high SDI countries and Latin America, probably reflecting concerted efforts to implement strong tobacco control policies and programmes in Brazil and Panama, among others.

Yet amid these gains, many countries with persistently high levels of daily smoking recorded marginal progress since 2005, and smoking remained among the leading risk factors for early death and disability in more than 100 countries in 2015, accounting for 11·5% of global deaths (95% UI 10·3–12·6) and 6·0% (5·3–6·8) of global DALYs. Smoking patterns diverged by geography, level of development, sex, and birth cohort, emphasising the need for tailored approaches to change smoking behaviours. Although male smoking prevalence still far exceeded that of female smokers in 2015, the most pronounced reductions in smoking prevalence since 1990 were generally found for men—and more places saw minimal changes or increases in smoking among women. These trends highlight how the tobacco epidemic, and corresponding industry forces, has expanded beyond a male-centred health challenge.

Low to middle SDI countries saw increased disease burden attributable to smoking since 2005, a trend that that occurred despite variable decreases in smoking prevalence and risk-deleted DALY rates. Population growth or ageing, or a combination of both, ultimately contributed to increased disease burden attributable to smoking in these countries. In higher SDI countries, population growth and ageing offset the potential for larger gains in places where notable declines in smoking prevalence and risk-deleted DALY rates occurred. This finding points to a crucial challenge ahead for tobacco control: unless progress in reducing current smoking and preventing initiation can be substantially accelerated, demographic forces, which are far less amenable to immediate intervention, are poised to heighten the disease burden associated with smoking's global toll.

Since 2005, the year when the FCTC entered into force, it has redefined global, regional, and national approaches to tobacco control and policy.^50^, ^51^ Case studies point to the successful uptake and enforcement of FCTC components in many countries with especially prominent reductions in smoking prevalence. Pakistan, Panama, and India stand out as three countries that have implemented a large number of tobacco control policies over the past decade and have had marked declines in the prevalence of daily smoking since 2005, compared with decreases recorded between 1990 and 2005.^52^, ^53^, ^54^ At the same time, many countries, including Australia, Brazil, Canada, South Korea, and the USA, among others, achieved sizeable declines in smoking prevalence well before FCTC adoption.^15^, ^56^, ^57^, ^58^ Altogether, 18 countries recorded a faster annualised rate of decline from 1990 to 2005 than from 2005 to 2015.

Brazil, which has achieved the third largest significant decline in age-standardised smoking prevalence since 1990, is a noteworthy success story. Brazil accomplished this reduction through a combination of tobacco control policies that began with advertising restrictions and smoking bans in some public places starting in 1996 and culminated with Brazil achieving the highest level of achievement in all MPOWER measures except for monitoring by 2011. Policies were comprehensive and were supplemented with fiscal interventions that included raising taxes and establishing minimum prices for tobacco products. Finally, Brazil has achieved high levels of compliance through enforcement.^20^, ^59^, ^60^, ^61^, ^62^

Critics of the FCTC argue that the treaty's effectiveness may be limited in various settings, especially since compliance has lagged in many countries.^63^, ^64^, ^65^ The FCTC, while necessary and vital for creating the policy environment for more effective tobacco control worldwide, is not sufficient to fully address each country's tobacco control needs. Rather, countries will need to both implement FCTC-stipulated measures and supplement such policies and programmes with strong enforcement and high rates of compliance. For example, India, where 11·2% of the world's smokers live, supplemented the Cigarettes and Other Tobacco Products Act (COTPA) with the creation of a National Tobacco Control Programme (NTCP) in 2007. NTCP was created to strengthen implementation and enforcement of the various provisions of COTPA at the state and district level. It has been rolled out in phases and currently covers about 40% of all districts in India.^66^

Despite concerted efforts to control tobacco around the world, there remain a number of countries where current levels and recent trends raise concern. For example, Indonesia, a country with very high levels of smoking, particularly among men, has not yet ratified the FCTC and scores very poorly on the MPOWER indicators.^18^ Also, in Russia, prevalence among women has been increasing, and, until recently, there were very few laws related to tobacco control.^67^ Russia passed a comprehensive tobacco control policy in 2014 and has the potential to achieve progress on tobacco control.^68^ As a region, eastern Europe has seen a statistically significant increase in smoking prevalence among women since 1990. Increases among women, along with a sustained high prevalence of male smokers, can be linked to tobacco industry targeting during the 1990s.^6^ The tobacco industry is now turning its focus toward emerging markets in sub-Saharan Africa, seeking to exploit the continent's patchwork tobacco control regulations and limited resources to combat industry marketing advances.^69^, ^70^ Given the large effects of population growth and ageing on smoking-attributable disease burden—and Africa's rapidly changing demographic profile—a renewed dedication to strong, proactive tobacco policies and monitoring will be vital for the continent.^71^

The 2030 agenda features tobacco control as a key component to sustainable development, with SDG Target 3.a calling for stronger FCTC implementation.^24^ Nonetheless, the utility and potential impact of the SDGs on tobacco control may be hindered by the vagueness of Target 3.a (“Strengthen the implementation of the WHO FCTC in all countries, as appropriate”) and absence of defined targets for reducing smoking prevalence by 2030. Ultimately, to move all countries toward stronger tobacco control by 2030, improvements in policy formulation, enforcement and compliance, and the routine monitoring of smoking behaviour are urgently needed. Without valid and reliable data, these efforts risk being more aspirational than grounded in evidence-informed action. Multi-country survey series have substantially improved data availability on smoking prevalence, yet the disadvantages associated with such surveys—high cost, time lags, inconsistent questions across survey series, sample restrictions for young populations, and a reliance on self-reported smoking behaviour—necessitate the development of robust, locally focused, timely, objective, and low-cost methods of tracking smoking trends. Supplementing surveys with biomarker collection is essential because self-reported smoking prevalence is believed to be severely underestimating true smoking prevalence,^72^, ^73^, ^74^, ^75^ especially in population subgroups or places where tobacco use may not be culturally acceptable.

Our findings should be interpreted taking into consideration the study's limitations. First, our exposure estimation focused on smoked tobacco and did not include smokeless tobacco products and e-cigarettes. Second, our definition of smoking exposure pertained to current daily smokers, and did not include occasional or former smokers, which might underestimate the attributable disease burden to smoking, especially in populations who tend to be less likely to smoke every day, such as women, children and young adults, and individuals with less disposable income. Third, we did not account for the intensity or duration of smoking. Fourth, the study relied on self-reported data, and it is possible that reporting biases varied across countries and over time. Fifth, for long-term effects of smoking on cancers and chronic respiratory diseases, we used the smoking impact ratio method, which estimates the lifetime cumulative effect of cigarette smoking using the proxy of recorded lung cancer mortality rates. This method provides robust estimates of the burden of cancers and chronic respiratory diseases related to tobacco but is not fully consistent with the GBD approach of estimating exposure independently of the outcomes affected by exposure. Also, the smoking impact ratio method is based on the cumulative effect of cigarette smoking rather than all types of tobacco smoking, and might be less robust for geographies in which non-smoker lung cancer might be significantly affected by air pollution or other factors. Sixth, our estimates of DALYs are probably underestimates because relative risk values used for estimating population attributable fractions might not fully represent all possible risk-outcome pairs experienced by sex, age group, and over time.^76^ Also, burden estimates did not account for the effect of both indoor and outdoor air pollution potentiating risks. Finally, minimal risk-outcome data were available for populations younger than 30 years, and therefore burden attribution was limited to age groups 30 years and older.

## Discussion

Despite more than 50 years of anti-tobacco efforts, smoking remains a leading global risk factor. Its toll will remain substantial without more concerted policy initiatives, policy compliance and enforcement, and sustained political will to offset commercial interests. Despite progress in some settings, the war against tobacco is far from won, especially in countries with the highest numbers of smokers. The staggering toll of smoking on health echoes well beyond the individual, especially as tobacco threatens to exact long-term financial and operational burdens on already resource-constrained health systems. To significantly and permanently bend the global tobacco epidemic's trajectory, a renewed and sustained focus is needed on comprehensive tobacco control policies around the world. Success is possible, but requires effective and aggressively enforced policies and laws. Intensified efforts are also greatly needed to keep smoking prevalence rates low in populations which have not experienced a devastating epidemic yet, and to prevent children, adolescents, and young adults from starting to smoke.

Correspondence to: Dr Emmanuela Gakidou, Institute for Health Metrics and Evaluation, University of Washington, Seattle, WA 98121 **gakidou@uw.edu**
