# Supplementary material for: Smoking prevalence and attributable disease burden in 195 countries and territories, 1990–2015: a systematic analysis from the Global Burden of Disease Study 2015
Source: Lancet. 2017 May 13;389(10082):1885–906. doi: 10.1016/S0140-6736(17)30819-X (PMC5439023; doi:10.1016/S0140-6736(17)30819-X)
Supplement: Supplementary appendix [file mmc1.pdf]

THE LANCET

Supplementary appendix

This appendix formed part of the original submission and has been peer reviewed. We post it as supplied by the authors.

Supplement to: GBD 2015 Tobacco Collaborators. Smoking prevalence and attributable disease burden in 195 countries and territories, 1990–2015: a systematic analysis from the Global Burden of Disease Study 2015. *Lancet* 2017; published online April 5. http://dx.doi.org/10.1016/S0140-6736(17)30819-X.

Supplementary Material

Estimating smoking prevalence and attributable disease burden in 195 countries and territories, 1990-2015: a systematic analysis from the Global Burden of Disease Study 2015.

GBD 2015 Tobacco Collaborators

Contents

Supplemental Figures and Tables 3
Geographies estimated and Socio-demographic Index 5
Age-standardisation 5
Data and modeling flowchart 5
Inclusion criteria 5
Data sources 5
    Consumption 5
    Prevalence 6
    Smoking Impact Ratio 6
    Burden 6
    Relative risk 6
Consumption data preparation 6
Consumption modeling 7
Smoking prevalence data preparation 7
    Data extraction 7
    Crosswalking 7
Smoking prevalence modeling 8
Smoking Impact Ratio calculation 9
Estimating attributable burden 9
    Assessment of risk-outcome pairs 9
    Relative risk 9
    Population Attributable Fraction (PAF) calculation 9
    Attributable burden calculation 9
Decomposition 10
Supplemental results 10
References 11

Supplemental Figures and Tables

Figure S1. Estimation flowchart including data inputs, database inputs, modeling processes, and results.

Figure S2. Prevalence of daily smoking for men (A) and women (B), aged 15-19, in 2015. ATG=Antigua and Barbuda; VCT= Saint Vincent and the Grenadines; BRB=Barbados; COM=Comoros; DMA=Dominica; GRD=Grenada; MDV=Maldives; MUS=Mauritius; LCA=Saint Lucia; TTO=Trinidad and Tobago; TLS=Timor-Leste; SYC=Seychelles; W Africa=Western Africa; E Med.=Eastern Mediterranean; MLT=Malta; SGP=Singapore; MHL=Marshall Islands; KIR=Kiribati; SLB=Solomon Islands; FSM=Federated States of Micronesia; VUT=Vanuatu; WSM=Samoa; FJI=Fiji; TON=Tonga.

Figure S3. Percent change in age-standardized smoking prevalence from 1990 to 2015, for men (A) and women (B). Countries without a statistically significant change from 1990 to 2015 are indicated in grey.

Figure S4. Prevalence of daily smoking across birth cohorts over time, at the global level and by super region, for men (A) and women (B). Birth cohorts are colour-coded by five-year intervals, with the most recent birth cohort in red (2005) to the least recent birth cohort in dark blue (1910). Each dot represents the prevalence of daily smoking for a given birth cohort and age group.

Figure S5. Leading 30 level 3 global causes for DALYs attributable to smoking for males (A) and females (B), 1990, 2005, and 2015 with percentage change in the number of DALYs, and all-age, and age-standardised rates. Causes are connected by arrows between time periods. For the time period 1990 to 2005 and for 2005 to 2015, three measures of change are shown: percent change in the number of DALYs, percent change in the all-age DALY rate and percent change in the age-standardised DALY rate. Changes that are statistically significant are shown in bold. DALYs=disability-adjusted life-years.

Figure S6. Rankings of smoking as a risk factor for all-cause, all-age attributable DALYs for both sexes combined in 1990 (A) and 2005 (B). DALYs = disability-adjusted life-years. ATG=Antigua and Barbuda; VCT= Saint Vincent and the Grenadines; BRB=Barbados; COM=Comoros; DMA=Dominica; GRD=Grenada; MDV=Maldives; MUS=Mauritius; LCA=Saint Lucia; TTO=Trinidad and Tobago; TLS=Timor-Leste; SYC=Seychelles; W Africa=Western Africa; E Med.=Eastern Mediterranean; MLT=Malta; SGP=Singapore; MHL=Marshall Islands; KIR=Kiribati; SLB=Solomon Islands; FSM=Federated States of Micronesia; VUT=Vanuatu; WSM=Samoa; FJI=Fiji; TON=Tonga.

Table S1. Geographies estimated, SDI value, and SDI level. SDI = Socio-demographic Index. SDI values and levels reflect 2015 estimates for the component factors: total fertility rate, mean educational attainment, and lag-distributed income per capita.

Table S2. Citations for sources used in relative risk estimation.

Table S3. Number of data points extracted for each frequency-type indicator. Data points are location-, year-, age-, sex-specific. Columns are different frequencies of tobacco use captured. Rows are different types of tobacco captured.

Table S4. Crosswalking coefficients, model fit, and number of data points using each crosswalk. Data points are location-, year-, age-, sex-specific. *Used to crosswalk data from sources that did not have

data on any of the five other frequency-type combinations with crosswalk models with R-squared values > 0.8.

Table S5. Strength of evidence supporting a causal relationship between smoking and 38 included outcomes. Prospective observational studies with a significant association in the opposite direction is missing if there were no prospective observational studies or the strength of evidence was assess through a pooled analysis. Biologic plausibility indicates whether any biological or mechanistic pathway exists that could potentially explain the relationship between smoking and the outcome. Analogy indicates whether smoking is associated with another outcome from the same category and whether any evidence exists that smoking can cause the current outcome through the same biological or mechanistic pathway.

Table S6. Citations for evidence used in assessing causal relationships between smoking and 38 included outcomes.

Table S7. Relative-risk estimates, by age and sex, for 38 included outcomes.

Table S8. All-cause all-age (30+) deaths and DALYs and all-cause age-standardized mortality and DALY rates (per 100) in 2015 for men and women. 95% uncertainty intervals are reported in parentheses: (lower, upper).

Geographies estimated and Socio-demographic Index

Consistent with the protocol of the Global Burden of Disease Study 2015, we estimated smoking prevalence from 1980-2015 for 195 countries and territories (Table S1), both sexes, and 15 five-year age groups (age group 10-14 through age group 80+) . We estimated smoking attributable burden for the same 195 countries and territories from 1990-2015, both sexes, and 11 five-year age groups (age group 30-34 through age group 80+). Additionally, we calculated aggregate estimates by geography and level of development. Geographic aggregates (regions, super regions, and global) are consistent with the previously published GBD location hierarchy.[1] Aggregation by level of development used the Socio-demographic Index (SDI) level method used throughout GBD estimation to create five levels of development- high, high-middle, middle, low-middle, and low- to which there is a one-to-one map for all 195 countries and territories presented in this work.

Full details of calculation of SDI level were previously published.[1] Briefly, SDI is a composite indicator including total fertility rate, mean educational attainment, measured as number years of schooling completed among individuals ages 15+, and lag-distributed income per capita. Calculation of the composite indicator followed the methodology used in construction of the Human Development Index.[2] SDI values are included in Table S1, along with the geography's SDI level. SDI levels reflect 2015 values for the component indicators.

Age-standardisation

For age-standardised rates we used the GBD population standard.[1]

Data and modeling flowchart

Figure S1 shows the full estimation process, from data inputs to results.

Inclusion criteria

We included nationally representative survey data sources that captured information on primary tobacco use among individuals over age 10. We included only self-reported smoking data and excluded data from questions asking about others' smoking behaviors. We included data that was collected between 1 January 1980 and 31 December 2015 in any of the 195 geographies included in this study.

For consumption estimation, we included nationally representative supply-side data on tobacco availability from sources covering multiple countries. Data were included if they were collected between 1 January 1960 and 31 December 2015 and covered one of the 195 geographies included in this study.

Data sources

A complete list of sources is available from the GBD 2015 Data Input Sources Tool (http://ghdx.healthdata.org/gbd-2015/data-input-sources).

Consumption

We estimated country-wide consumption of tobacco as grams per capita using four supply-side data sources: 1) the United Nations Food and Agriculture Organization (FAO),[3] which covered 171 countries and territories from 1961-2013 and reported data as domestic supply in tonnes of tobacco; 2) the US Department of Agriculture (USDA),[4] which covered 153 countries from 1960 to 2005 and reported data as domestic consumption of manufactured cigarettes; 3) Euromonitor,[5] which covered 80 countries from 1998-2015 and reported data as sales of cigarettes including manufactured, roll-your-own, and

illicit sales; and 4) sales data (personal correspondence from Christopher Tan, MBA, September 2013), which covered 18 countries from 1997 to 2012.

Prevalence

We searched the Global Health Data Exchange (GHDx) database for primary data sources with the keyword "Tobacco Use" on 1 May 2015 and again on 16 February 2016 to ensure all available data sources were captured. Of the 3,318 sources identified in the GHDx, 2,224 sources met inclusion criteria and were included.

In addition to the primary data sources identified through the GHDx, we supplemented with secondary database estimates from the WHO InfoBase Database and International Smoking Statistics Database for sources for which primary data are unavailable. We included 281 sources from the WHO InfoBase and 313 sources from the International Smoking Statistics Database.

Smoking Impact Ratio

The Smoking Impact Ratio (SIR) is computed using four estimates: 1) lung cancer mortality rates in a reference population of smokers, 2) lung cancer mortality rates in a reference population of never-smokers, 3) lung cancer mortality rates among never smokers in a population of interest, and 4) observed lung cancer mortality rates in a population of interest. We used available prospective cohort studies to estimate values 1, 2, and 3. A list of included prospective cohorts is available in the GBD 2015 Data Input Sources Tool (http://ghdx.healthdata.org/gbd-2015/data-input-sources). We used lung cancer mortality rate estimates from GBD 2015 for value 4.[6]

Burden

Inputs to calculate attributable burden included GBD 2015 deaths and DALYs for the 38 outcomes included in this analysis. Estimates are publically available through the GBD Results Tool: http://ghdx.healthdata.org/gbd-results-tool.

Relative risk

Relative risk estimates were derived from prospective cohort studies. Sources used in relative risk estimation are reported in Table S2.

Consumption data preparation

To prepare the supply-side tobacco consumption data for modeling, first we converted sources reporting data in units of cigarettes to units of grams of tobacco by equating one cigarette to one gram of tobacco. Next, we transformed all data to per capita estimates using GBD population estimates. Data reported for aggregated former geographies, such as the Soviet Union and the Socialist Federal Republic of Yugoslavia, were split using the ratios derived from four years after dissolution. Since FAO data were reported as availability of tobacco, not as sales or consumption, we assumed that there was noise resulting from compensation for overstock or understock. Since our metric of interest was consumption, we smoothed FAO data by taking a four-year historical average. To enforce consistency between prevalence and consumption, we assumed that grams per daily smoker per day would not exceed 30 and would not be less than five. Data outside these thresholds were considered to be outliers and were dropped. We controlled for differences between sources by crosswalking data from each source to the average of data from other sources paired by country-year. Uncertainty of data was estimated by calculating the standard deviation of data over a 10-year rolling window by source.

Consumption modeling

We used spatiotemporal Gaussian process regression (ST-GPR) to model supply-side tobacco consumption. We updated the mean function used previously by Ng. et al to the following:

$$m_{g,a,s}(t) = X\beta + h(r_{g,t})$$

where $h(r_{g,t})$ is a smoothing function for residuals from the mixed effects model that draws strength across geography and time, and $X\beta$ is the estimate produced by the following mixed effects model, fit using restricted maximum likelihood in R:

$$\log(\text{consumption})_{g,t} = \beta_0 + \beta_1 \text{education}_{g,t} + \alpha_s + \alpha_r + \alpha_g + \epsilon_{g,t}$$

where $\text{education}_{g,t}$ is the mean years of education completed among individuals over age 15 in geography g at time t, and α_s, α_r, and α_g are super region, region, and geography random intercepts, respectively. The linear model component ($X\beta$) was fit using random effects, but predictions were made based only on the estimated beta coefficients. Differences from the previously published model include a different covariate and a different model specification. We used educational attainment, as opposed to the previously used GDP, based on out-of-sample cross validation analyses. We used spatiotemporal smoothing ($h(r_{g,t})$), as opposed to the previously used region and geography random slopes to better capture the effect of time and incorporate more data in estimating the mean function in data-sparse regions. The ST-GPR method has been described in detail elsewhere.[1]

Smoking prevalence data preparation

Data extraction

We extracted primary data from individual-level microdata and survey report tabulations. We extracted data on current tobacco use reported as any combination of frequency of use (daily, occasional, and current, which includes both daily and occasional smokers) and of type of tobacco used (all tobacco including smoked and smokeless, all tobacco excluding cigarettes, smoked tobacco, cigarettes only, manufactured cigarettes, hand-rolled cigarettes, smoked tobacco excluding cigarettes, and smokeless tobacco), resulting in 24 possible combinations for frequency and type.

For microdata, we extracted relevant demographic information, including age, sex, location, and year, as well as survey metadata, including survey weights, primary sampling units, and strata. This information allowed us to tabulate individual-level data in the standard GBD five-year age-sex groups and produce accurate estimates of uncertainty. For survey report tabulations, we extracted data at the most granular age-sex group provided.

Crosswalking

Our gold-standard smoking prevalence indicator is daily use of smoked tobacco products. All other data points were adjusted to be consistent with this definition. Table S3 shows the number of data points extracted for each indicator. Some sources contained information on more than one frequency-type combination indicator and these sources were used to develop the adjustment coefficient to transform that alternative frequency-type combinations to the gold-standard frequency-type combination of daily use of smoked tobacco. The adjustment coefficient was the beta value derived from the following model:

$$p_{daily-smoked,k} = \beta p_{i,k} + \epsilon_k$$

where $p_{daily-smoked,k}$ is the prevalence of daily smoking reported in survey k and $p_{i,k}$ is the prevalence of an alternative frequency-type combination i also reported in survey k. Models with adjusted R-squared values > 0.8 were used in order of their R-squared value. Adjusting for frequency and type simultaneously improved upon the previously published method[7] by taking into account correlation between frequency and type. Five alternative frequency-type combinations had adjusted R-squared values > 0.8. For other alternative frequencies and types we used the previously published step-wise crosswalk, adjusting for frequency and type independently. Sources only reporting data on occasional use (n=202 data points, 0.2%) were dropped because there was no sufficiently accurate crosswalk available. The estimated regression coefficients used for adjustment and the number of data points using that adjustment are reported in Table S4. Crosswalking models were fit using Stata 13.

We propagated uncertainty at the survey (k) level from the crosswalk using the following equation:

$$PE_k = \sigma_\epsilon^2 + X_k^2 var(\hat{\beta})$$

where PE_k is the crosswalk prediction error that is added to the sampling variance of the data point, σ_ϵ^2 is the variance of the error, X_k^2 is the squared value of the data being adjusted, and $var(\hat{\beta})$ is the variance of the adjustment coefficient.

Smoking prevalence modeling

We used ST-GPR to model smoking prevalence given the abundance of age and sex-specific data. Full details on the ST-GPR method have been previously published.[1] Briefly, the mean function input to GPR is a complete time series of estimates generated from a mixed effects hierarchical linear model plus weighted residuals smoothed across time, space, and age. The linear model formula, fit separately by sex using restricted maximum likelihood in R, is:

$$logit(p_{g,a,t}) = \beta_0 + \beta_1 CPC_{g,t} + \sum_{k=2}^{16} \beta_k I_{A[a]} + \alpha_s + \alpha_r + \alpha_g + \epsilon_{g,a,t}$$

where $CPC_{c,t}$ is the tobacco consumption covariate, by geography g and time t, described above, $I_{A[a]}$ is a dummy variable indicating specific age group A that the prevalence point $p_{g,a,t}$ captures, and α_s, α_r, and α_g are super region, region, and geography random intercepts, respectively. Random effects were used in model fitting but were not used in prediction.

We used out-of-sample cross validation for hyperparameter selection for the space (zeta), age (omega), and time (lambda) weights used in spatiotemporal smoothing along with the scale used in Gaussian process regression (details on the effects of different parameters have been previously published). We used a space weight of 0.95 in data-dense countries (at least five years covered in a geography-age-sex group) and space weight of 0.7 in data-sparse countries. The other parameters were consistent across data-density levels: age weight = 1, time weight = 1, and scale = 10.

We minimized out-of-sample root mean squared error (RMSE) in parameter selection, and also assessed the full model fit using RMSE. The in-sample RMSE of the final model was 0.05 and the out-of-sample RMSE was 0.08. The in-sample coverage of the final model was 99.4% and the out-of-sample coverage was 99.8%.

Smoking Impact Ratio calculation

We calculated SIR for each geography, year, age group, and sex included in attributable burden analysis using the following formula:

$$SIR = \frac{C_{LC} - N_{LC}}{S_{LC}^{*} - N_{LC}^{*}} \times \frac{N_{LC}^{*}}{N_{LC}}$$

where C_{LC} is the lung cancer mortality rate specific to the age-sex-geography-year of interest, N_{LC} is the age- sex- geography- year-specific lung cancer mortality rate of never-smokers in the population of interest, S_{LC}^{*} is the lung cancer mortality rate in a reference population of smokers, N_{LC}^{*} is the lung cancer mortality rate in a reference population of never-smokers. Additional details on SIR calculation can be found elsewhere.[8]

Estimating attributable burden

Assessment of risk-outcome pairs

We included outcomes based on the strength of available evidence supporting a causal relationship. Table S6 reports the strength of evidence for included outcomes and the exposure metric (5-year lagged prevalence or SIR) used for each outcome. Table S7 reports the sources used in evaluating strength of included outcomes.

Relative risk

Table S8 reports relative risk estimates and uncertainty for the 38 outcomes included in analysis, by age and sex where applicable. Sources used in generating relative risk estimates are cited in the GBD 2015 Data Input Sources Tool (http://ghdx.healthdata.org/gbd-2015/data-input-sources).

Population Attributable Fraction (PAF) calculation

We calculated population attributable fractions (PAFs) based on exposure estimates, relative risk estimates, and the theoretical minimum risk exposure level (TMREL), using the following formula for a dichotomous exposure:

$$PAF_{oasgt} = \frac{\sum_{x=0}^{1} RR_{oasg}(x)P_{asgt}(x) - RR_{oasg}(TMREL_{as})}{\sum_{x=0}^{1} RR_{oasg}(x)P_{asgt}(x)}$$

where PAF_{oasgt} is the population attributable fraction for outcome o, age group a, sex s, geography g, and year t. $RR_{oasg}(x)$ is the relative risk as a function of exposure level x for outcome o, age group a, sex s, and geography g; $P_{asgt}(x)$ is the proportion of the population with exposure level x for age group a, sex s, geography g, and year t; $TMREL_{as}$ is the TMREL for age group a, and sex s. The TMREL for smoking is zero, meaning nobody in the population smokes. Additional details on PAF calculation are published elsewhere.[1]

Attributable burden calculation

We calculated smoking attributable burden using the following formula for DALYs:

$$AB_{asgt} = \sum_{o=1}^{38} DALY_{oasgt} * PAF_{oasgt}$$

and deaths:

$$AB_{asgt} = \sum_{o=1}^{38} deaths_{oasgt} * PAF_{oasgt}$$

where AB_{asgt} is the total smoking attributable burden (as either DALYs or deaths) for a given age group a, sex s, geography g, time t, calculated from the sum of $deaths * PAF$ for outcomes o one through 38. Further details on calculation of attributable burden are published elsewhere.[1]

Decomposition

For the decomposition analysis, we used the methods developed by Das Gupta to decompose changes in total percent change in DALYs from 2005-2015 by SDI level due to changes in population structure (population ageing and population growth), risk-deleted DALY rate, which is the background DALY rate observed if smoking levels were at their TMREL, and risk exposure. Details on the decomposition methodology and implementation have been reported elsewhere.[1]

Supplemental results

Figure S2 shows age-specific smoking prevalence for men and women ages 20-24. Figure S4 shows cohort plots by super region.

References

1 Forouzanfar MH, Afshin A, Alexander LT, *et al.* Global, regional, and national comparative risk assessment of 79 behavioural, environmental and occupational, and metabolic risks or clusters of risks, 1990–2015: a systematic analysis for the Global Burden of Disease Study 2015. *The Lancet* 2016; **388**: 1659–724.

2 UNDP, editor. Work for human development. New York, NY: United Nations Development Programme, 2015.

3 FAOSTAT. http://faostat.fao.org/beta/en/#data (accessed Nov 8, 2016).

4 USDA Global Tobacco Database 1960-2005 | GHDx. http://ghdx.healthdata.org/record/usda-global-tobacco-database-1960-2005 (accessed Nov 8, 2016).

5 Cigarette industry market research, statistics, and market share. http://www.euromonitor.com/cigarettes?CountryCode=96650&IndustryCode=null&ContentType=null&ReportType=null&SortBy=1&PageNumber=0&PageSize=50&PageType=2 (accessed Nov 8, 2016).

6 Wang H, Naghavi M, Allen C, *et al.* Global, regional, and national life expectancy, all-cause mortality, and cause-specific mortality for 249 causes of death, 1980–2015: a systematic analysis for the Global Burden of Disease Study 2015. *The Lancet* 2016; **388**: 1459–544.

7 Ng M, Freeman MK, Fleming TD, *et al.* Smoking Prevalence and Cigarette Consumption in 187 Countries, 1980-2012. *JAMA* 2014; **311**: 183–92.

8 Ezzati M, Lopez AD. Smoking and oral tobacco use. *Comparative Quantification of Health Risks, WHO Publications* 2004. http://citeseerx.ist.psu.edu/viewdoc/download?doi=10.1.1.378.3697&rep=rep1&type=pdf#page=907 (accessed Nov 8, 2016).

Figure S1

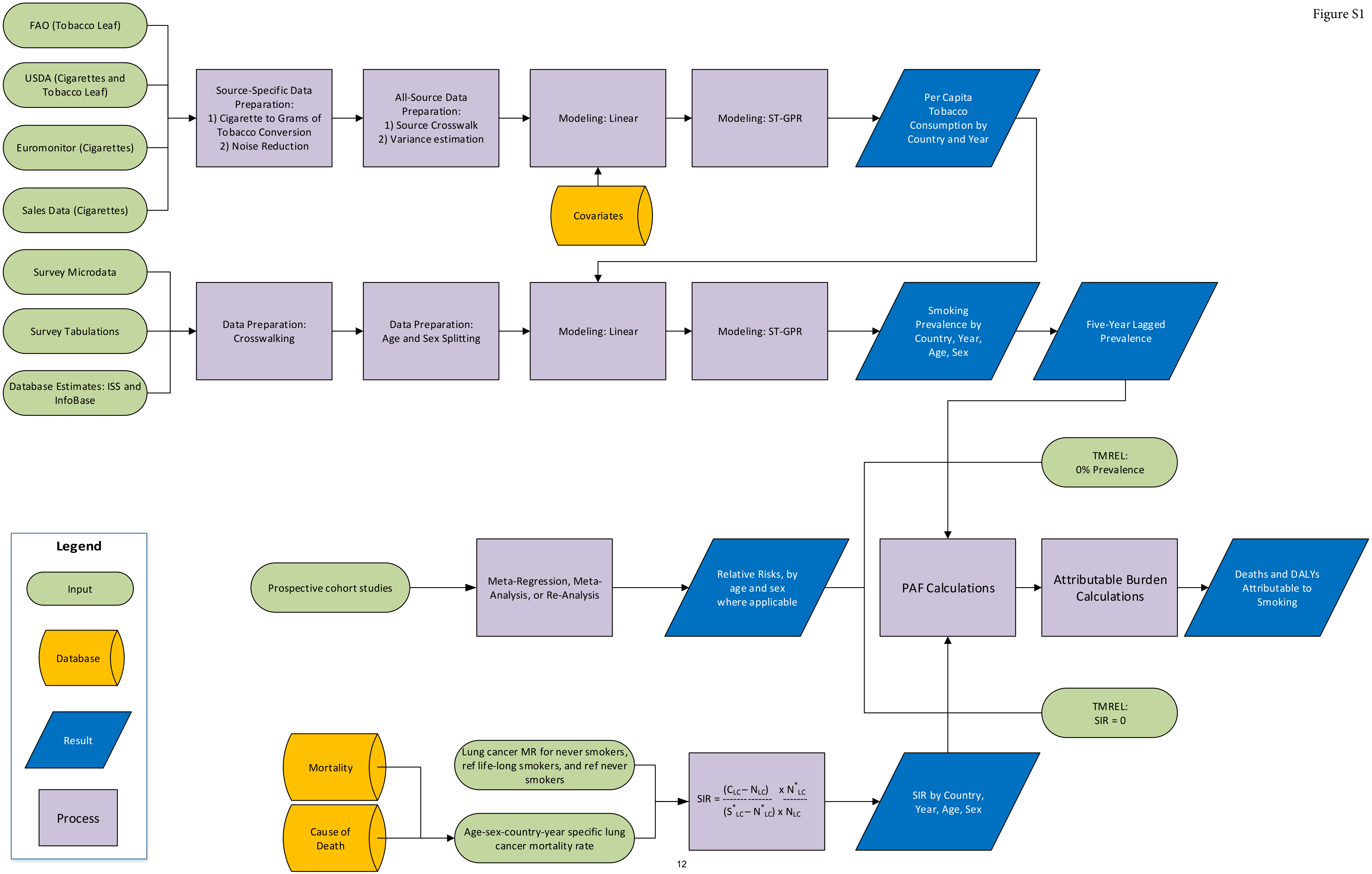
FAO (Tobacco Leaf)
USDA (Cigarettes and Tobacco Leaf)
Euromonitor (Cigarettes)
Sales Data (Cigarettes)
Source-Specific Data Preparation: 1) Cigarette to Grams of Tobacco Conversion 2) Noise Reduction
All-Source Data Preparation: 1) Source Crosswalk 2) Variance estimation
Modeling: Linear
Modeling: ST-GPR
Per Capita Tobacco Consumption by Country and Year
Covariates
Survey Microdata
Survey Tabulations
Database Estimates: ISS and InfoBase
Data Preparation: Crosswalking
Data Preparation: Age and Sex Splitting
Modeling: Linear
Modeling: ST-GPR
Smoking Prevalence by Country, Year, Age, Sex
Five-Year Lagged Prevalence
TMREL: 0% Prevalence
Legend
Input
Database
Result
Process
Prospective cohort studies
Meta-Regression, Meta-Analysis, or Re-Analysis
Relative Risks, by age and sex where applicable
PAF Calculations
Attributable Burden Calculations
Deaths and DALYs Attributable to Smoking
TMREL: SIR = 0
Mortality
Cause of Death
Lung cancer MR for never smokers, ref life-long smokers, and ref never smokers
Age-sex-country-year specific lung cancer mortality rate
$SIR = \frac{(C_{LC} - N_{LC})}{(S^*_{LC} - N^*_{LC})} \times \frac{N^*_{LC}}{N_{LC}}$
SIR by Country, Year, Age, Sex

Figure S2a

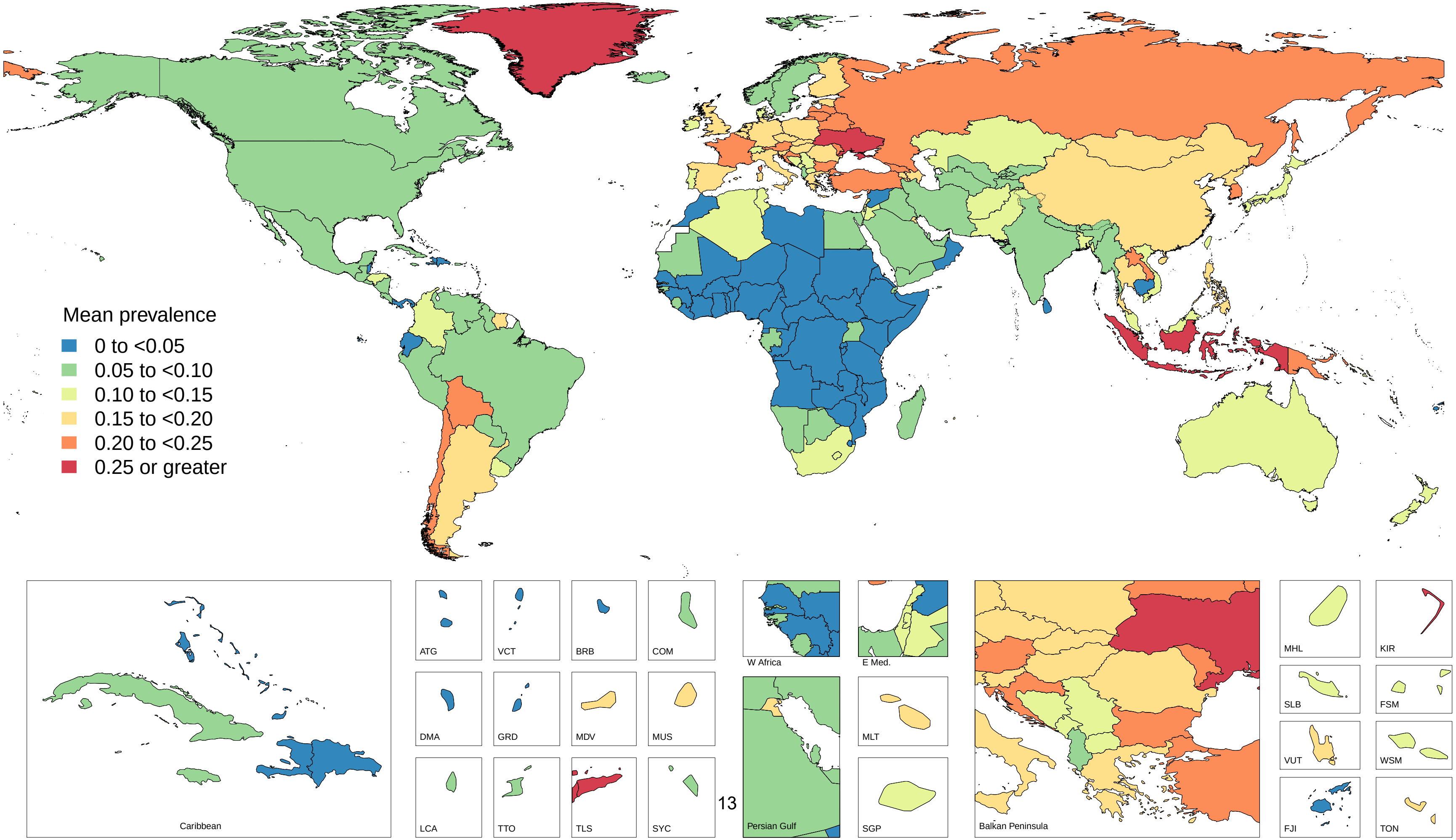
Mean prevalence
0 to <0.05
0.05 to <0.10
0.10 to <0.15
0.15 to <0.20
0.20 to <0.25
0.25 or greater
Caribbean
ATG
VCT
BRB
COM
DMA
GRD
MDV
MUS
LCA
TTO
TLS
SYC
W Africa
E Med.
Persian Gulf
MLT
SGP
Balkan Peninsula
MHL
KIR
SLB
FSM
VUT
WSM
FJI
TON

Figure S2b

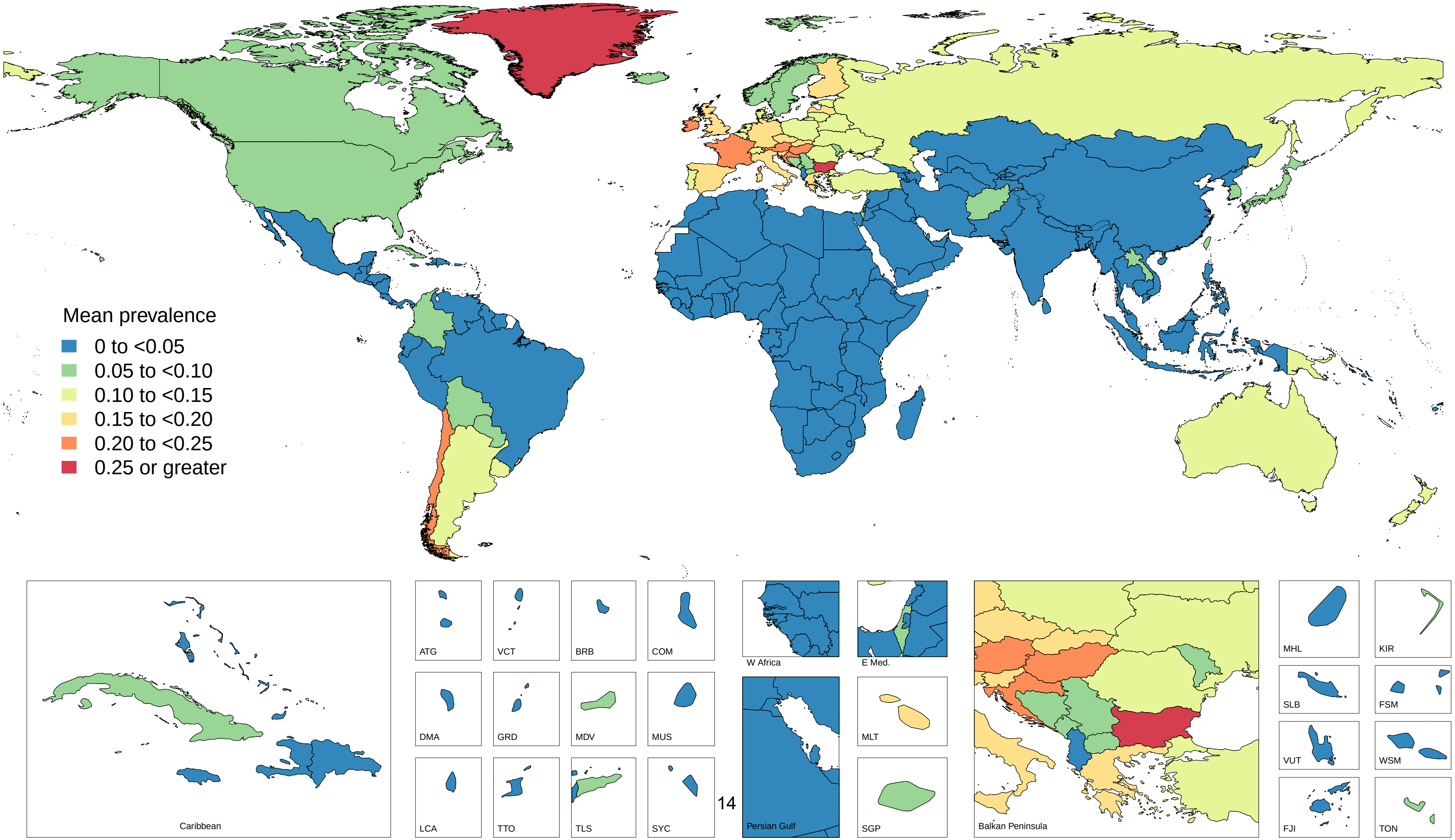
Mean prevalence
0 to <0.05
0.05 to <0.10
0.10 to <0.15
0.15 to <0.20
0.20 to <0.25
0.25 or greater
Caribbean
ATG
VCT
BRB
COM
DMA
GRD
MDV
MUS
LCA
TTO
TLS
SYC
W Africa
E Med.
Persian Gulf
MLT
SGP
Balkan Peninsula
MHL
KIR
SLB
FSM
VUT
WSM
FJI
TON

Figure S3a

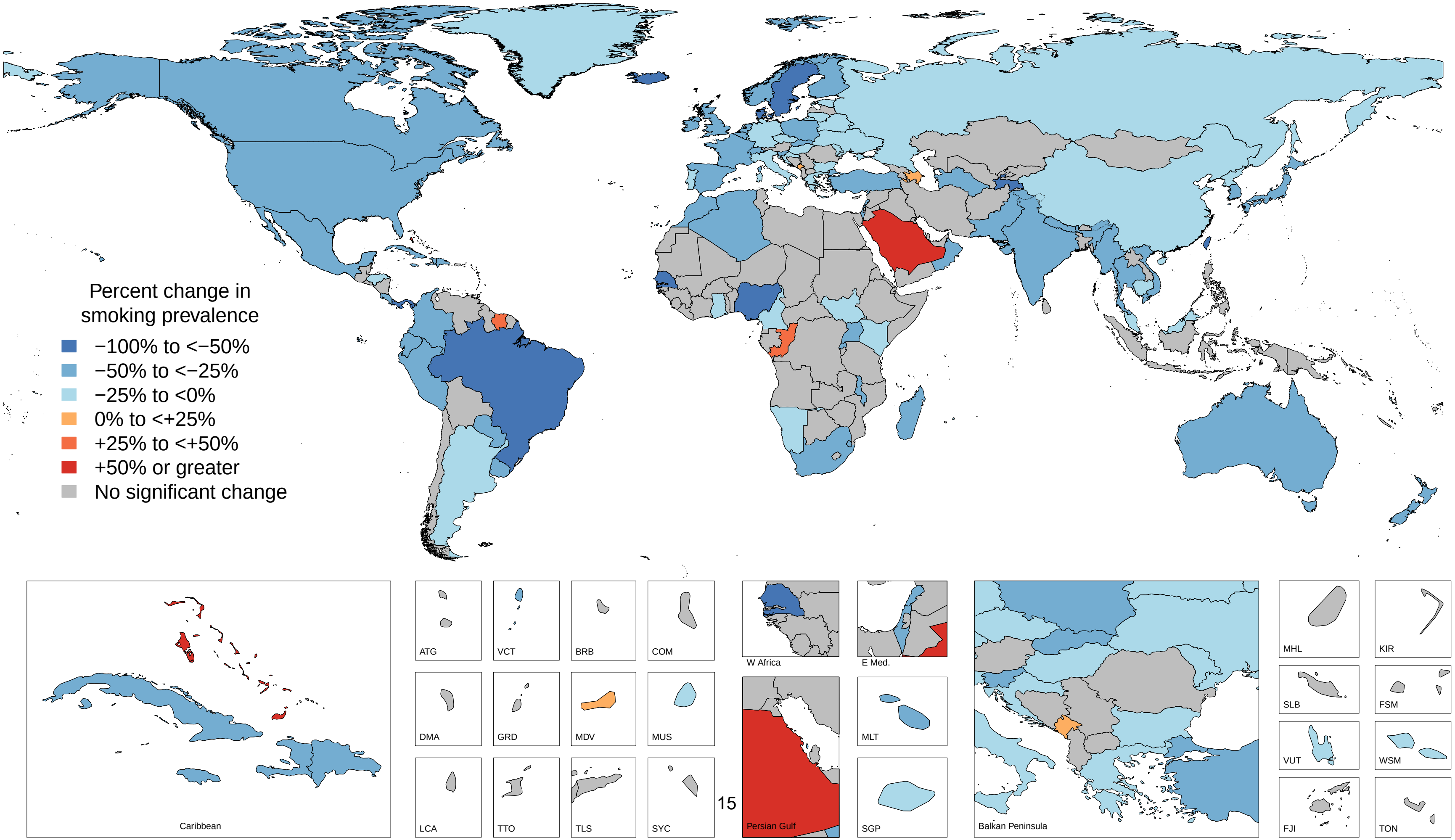

Percent change in smoking prevalence
−100% to <−50%
−50% to <−25%
−25% to <0%
0% to <+25%
+25% to <+50%
+50% or greater
No significant change
Caribbean
ATG
VCT
BRB
COM
DMA
GRD
MDV
MUS
LCA
TTO
TLS
SYC
W Africa
E Med.
Persian Gulf
MLT
SGP
Balkan Peninsula
MHL
KIR
SLB
FSM
VUT
WSM
FJI
TON

Figure S3b

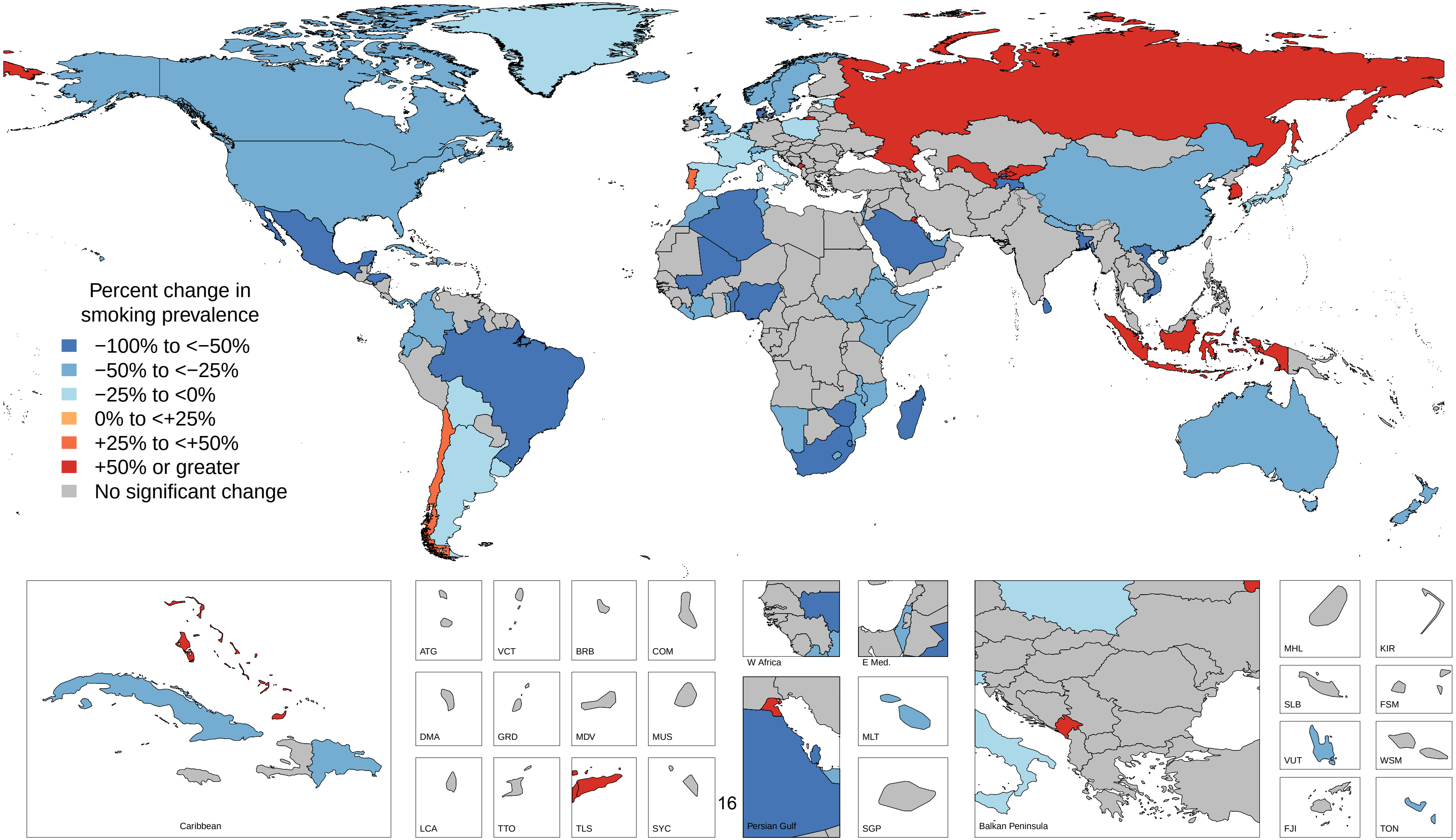

Percent change in smoking prevalence
−100% to <−50%
−50% to <−25%
−25% to <0%
0% to <+25%
+25% to <+50%
+50% or greater
No significant change
Caribbean
ATG
VCT
BRB
COM
DMA
GRD
MDV
MUS
LCA
TTO
TLS
SYC
W Africa
E Med.
Persian Gulf
MLT
SGP
Balkan Peninsula
MHL
KIR
SLB
FSM
VUT
WSM
FJI
TON

Figure S4a

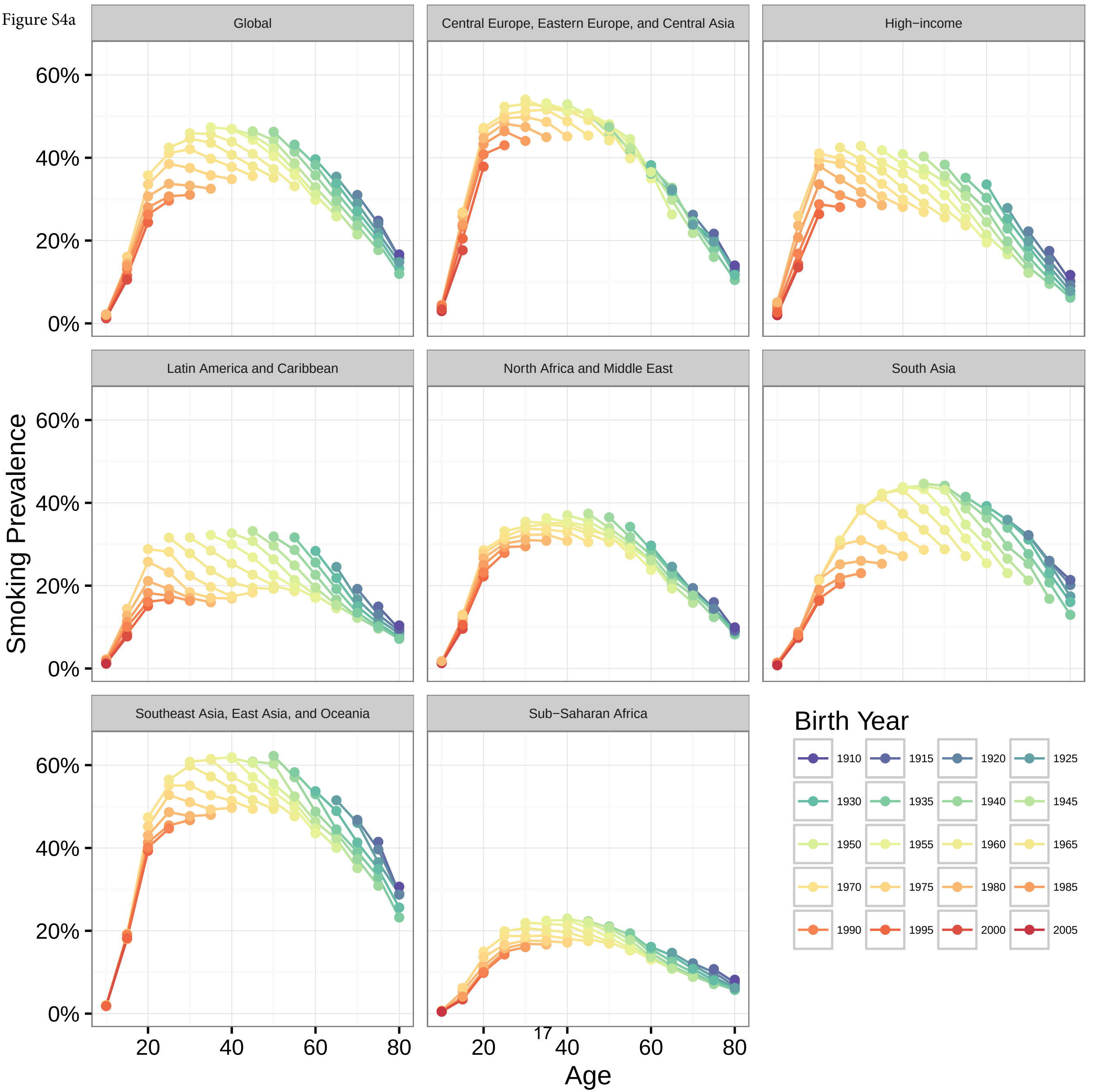
Global
Central Europe, Eastern Europe, and Central Asia
High−income
Latin America and Caribbean
North Africa and Middle East
South Asia
Southeast Asia, East Asia, and Oceania
Sub−Saharan Africa
Smoking Prevalence
0%
20%
40%
60%
Age
20
40
60
80
Birth Year
1910
1915
1920
1925
1930
1935
1940
1945
1950
1955
1960
1965
1970
1975
1980
1985
1990
1995
2000
2005

Figure S4b

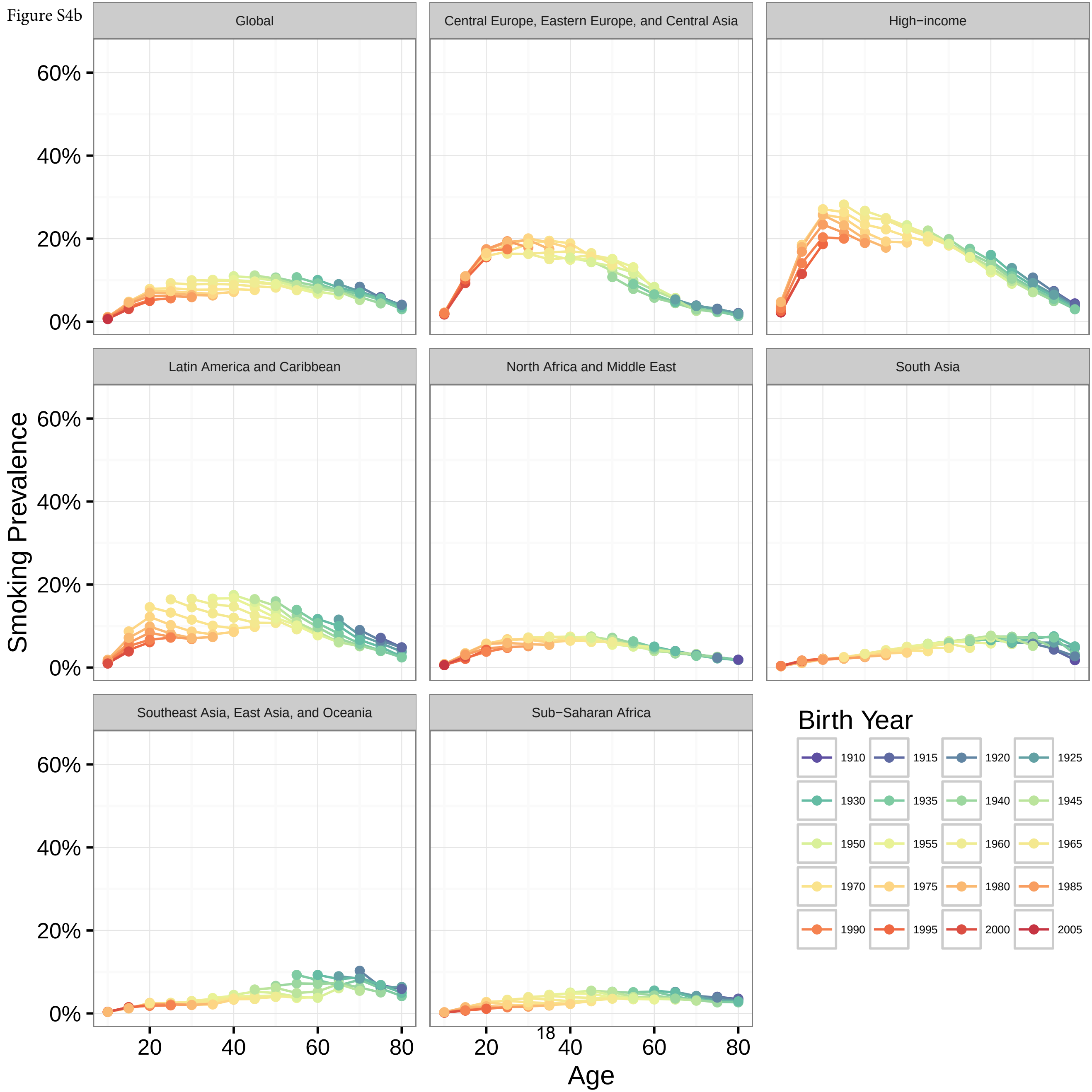
Global
Central Europe, Eastern Europe, and Central Asia
High−income
Latin America and Caribbean
North Africa and Middle East
South Asia
Southeast Asia, East Asia, and Oceania
Sub−Saharan Africa
Smoking Prevalence
0%
20%
40%
60%
20
40
60
80
Age
Birth Year
1910
1915
1920
1925
1930
1935
1940
1945
1950
1955
1960
1965
1970
1975
1980
1985
1990
1995
2000
2005

Figure S5a

Leading causes 1990	Leading causes 2005	% change number of DALYs 1990-2005	% change all-age DALY rate 1990-2005	% change age-standardised DALY rate 1990-2005	Leading causes 2015	% change number of DALYs 2005-2015	% change all-age DALY rate 2005-2015	% change age-standardised DALY rate 2005-2015
1 Ischemic heart disease	1 Ischemic heart disease	**23.1%**	-0.1%	**-15.7%**	1 Ischemic heart disease	3.2%	**-8.8%**	**-19.4%**
2 Cerebrovascular disease	2 Cerebrovascular disease	**25.9%**	2.3%	**-13.4%**	2 Cerebrovascular disease	-2.8%	**-14.1%**	**-24.6%**
3 COPD	3 Lung cancer	**22.4%**	-0.6%	**-13.8%**	3 Lung cancer	**10.2%**	-2.6%	**-15.5%**
4 Lung cancer	4 COPD	**15.8%**	-6.0%	**-19.5%**	4 COPD	6.3%	-6.0%	**-19.6%**
5 Lower respiratory infect	5 Lower respiratory infect	**24.2%**	0.8%	**-14.8%**	5 Lower respiratory infect	0.8%	**-10.9%**	**-20.9%**
6 Tuberculosis	6 Tuberculosis	**-15.6%**	**-31.4%**	**-41.7%**	6 Diabetes	**19.2%**	**5.3%**	**-6.9%**
7 Esophageal cancer	7 Esophageal cancer	**31.7%**	6.9%	-6.9%	7 Esophageal cancer	-4.6%	**-15.7%**	**-26.6%**
8 Hypertensive heart disease	8 Diabetes	**54.6%**	**25.5%**	**7.0%**	8 Tuberculosis	**-22.9%**	**-31.9%**	**-38.0%**
9 Asthma	9 Liver cancer	**46.8%**	**19.2%**	2.5%	9 Liver cancer	-0.2%	-11.8%	**-20.4%**
10 Stomach cancer	10 Hypertensive heart disease	-3.2%	**-21.4%**	**-33.9%**	10 Hypertensive heart disease	**12.9%**	-0.2%	**-11.4%**
11 Liver cancer	11 Asthma	-15.4%	**-31.3%**	**-41.9%**	11 Other cardiovascular	**9.9%**	-2.9%	**-13.5%**
12 Diabetes	12 Stomach cancer	-6.6%	**-24.2%**	**-34.2%**	12 Asthma	-8.4%	**-19.0%**	**-28.3%**
13 Other cardiovascular	13 Other cardiovascular	0.0%	**-18.8%**	**-32.1%**	13 Stomach cancer	**-15.2%**	**-25.1%**	**-33.9%**
14 Peptic ulcer disease	14 Peptic ulcer disease	**-19.0%**	**-34.2%**	**-42.8%**	14 Larynx cancer	4.9%	**-7.3%**	**-19.5%**
15 Larynx cancer	15 Larynx cancer	3.5%	**-15.9%**	**-27.0%**	15 Lip oral cavity cancer	**19.6%**	5.7%	**-7.3%**
16 Lip oral cavity cancer	16 Lip oral cavity cancer	**30.1%**	5.6%	**-9.9%**	16 Pancreatic cancer	**8.5%**	-4.1%	**-16.5%**
17 Pancreatic cancer	17 Pancreatic cancer	**22.7%**	-0.4%	**-14.5%**	17 Peptic ulcer disease	**-19.2%**	**-28.5%**	**-35.7%**
18 Bladder cancer	18 Bladder cancer	**13.3%**	**-8.0%**	**-21.3%**	18 Bladder cancer	**8.2%**	-4.3%	**-18.0%**
19 Colorectal cancer	19 Colorectal cancer	**11.6%**	**-9.4%**	**-22.3%**	19 Colorectal cancer	1.6%	**-10.2%**	**-21.7%**
20 Leukemia	20 Leukemia	**18.0%**	-4.2%	**-17.6%**	20 Leukemia	4.9%	-7.2%	**-17.2%**
21 Nasopharynx cancer	21 Nasopharynx cancer	**32.4%**	7.5%	-8.6%	21 Nasopharynx cancer	4.3%	-7.8%	**-16.9%**
22 Aortic aneurysm	22 Aortic aneurysm	**26.3%**	2.5%	**-14.9%**	22 Aortic aneurysm	5.8%	**-6.5%**	**-17.9%**
23 Kidney cancer	23 Atrial fibrillation	**25.9%**	**2.2%**	**-14.7%**	23 Atrial fibrillation	**15.8%**	**2.4%**	**-12.2%**
24 Atrial fibrillation	24 Kidney cancer	**24.2%**	0.8%	**-13.3%**	24 Kidney cancer	**10.1%**	-2.6%	**-15.2%**
25 Falls	25 Road injuries	**35.9%**	**10.4%**	**-6.5%**	25 Road injuries	-0.4%	**-12.0%**	**-20.5%**
26 Road injuries	26 Falls	**8.4%**	**-12.0%**	**-22.3%**	26 Falls	2.7%	**-9.3%**	**-19.2%**
27 Sense organ diseases	27 Sense organ diseases	**32.0%**	**7.2%**	**-10.1%**	27 Sense organ diseases	**13.9%**	0.7%	**-14.1%**
28 Other chronic respiratory	28 Interstitial lung disease	**55.0%**	25.8%	9.8%	28 Interstitial lung disease	**23.8%**	9.4%	-5.2%
29 Rheumatoid arthritis	29 Rheumatoid arthritis	**20.8%**	-1.9%	**-14.3%**	29 Rheumatoid arthritis	**11.3%**	-1.6%	**-11.5%**
30 Interstitial lung disease	30 Other chronic respiratory	-9.5%	**-26.6%**	**-37.5%**	30 Other chronic respiratory	10.9%	-2.0%	**-12.0%**

Legend:

Communicable, maternal, neonatal and nutritional

Non-communicable

Injuries

Figure S5b

Leading causes 1990	Leading causes 2005	% change number of DALYs 1990-2005	% change all-age DALY rate 1990-2005	% change age-standardised DALY rate 1990-2005	Leading causes 2015	% change number of DALYs 2005-2015	% change all-age DALY rate 2005-2015	% change age-standardised DALY rate 2005-2015
1 COPD	1 COPD	14.8%	-6.5%	-17.9%	1 COPD	-5.5%	-16.3%	**-27.0%**
2 Ischemic heart disease	2 Ischemic heart disease	-3.2%	**-21.2%**	**-31.6%**	2 Lung cancer	6.8%	-5.4%	**-16.9%**
3 Cerebrovascular disease	3 Lung cancer	**47.9%**	**20.5%**	5.9%	3 Ischemic heart disease	**-7.8%**	**-18.4%**	**-28.3%**
4 Lung cancer	4 Cerebrovascular disease	-3.5%	**-21.4%**	**-31.6%**	4 Cerebrovascular disease	**-18.5%**	**-27.8%**	**-36.3%**
5 Lower respiratory infect	5 Lower respiratory infect	**6.9%**	**-12.9%**	**-24.1%**	5 Lower respiratory infect	**-9.7%**	**-20.0%**	**-29.2%**
6 Asthma	6 Esophageal cancer	**32.1%**	7.6%	-5.4%	6 Esophageal cancer	-25.7%	**-34.2%**	**-42.4%**
7 Esophageal cancer	7 Other cardiovascular	-2.7%	**-20.7%**	**-31.4%**	7 Other cardiovascular	-1.2%	**-12.5%**	**-22.0%**
8 Other cardiovascular	8 Asthma	-12.8%	**-29.0%**	**-38.3%**	8 Hypertensive heart disease	-5.4%	**-16.3%**	**-26.2%**
9 Hypertensive heart disease	9 Hypertensive heart disease	-10.0%	**-26.7%**	**-36.5%**	9 Asthma	**-11.5%**	**-21.7%**	**-29.4%**
10 Tuberculosis	10 Cervical cancer	**23.9%**	0.9%	**-13.4%**	10 Pancreatic cancer	**15.6%**	2.3%	**-10.6%**
11 Cervical cancer	11 Pancreatic cancer	**44.6%**	**17.8%**	3.6%	11 Colorectal cancer	2.3%	**-9.4%**	**-20.4%**
12 Stomach cancer	12 Colorectal cancer	**25.6%**	2.3%	**-10.5%**	12 Cervical cancer	-7.1%	**-17.8%**	**-25.3%**
13 Colorectal cancer	13 Liver cancer	**36.0%**	10.8%	-1.9%	13 Larynx cancer	7.2%	-5.1%	**-16.6%**
14 Peptic ulcer disease	14 Stomach cancer	2.0%	**-16.9%**	**-27.0%**	14 Lip oral cavity cancer	**23.3%**	9.1%	-4.0%
15 Larynx cancer	15 Tuberculosis	**-23.9%**	**-38.0%**	**-46.1%**	15 Liver cancer	-12.4%	-22.4%	**-30.5%**
16 Pancreatic cancer	16 Larynx cancer	9.1%	**-11.1%**	**-22.3%**	16 Stomach cancer	**-20.4%**	**-29.5%**	**-37.0%**
17 Liver cancer	17 Lip oral cavity cancer	**43.3%**	**16.8%**	1.4%	17 Tuberculosis	**-33.4%**	**-41.0%**	**-46.9%**
18 Lip oral cavity cancer	18 Peptic ulcer disease	**-22.5%**	**-36.9%**	**-44.4%**	18 Diabetes	**6.6%**	**-5.6%**	**-17.1%**
19 Diabetes	19 Diabetes	**21.3%**	-1.2%	**-13.5%**	19 Peptic ulcer disease	**-17.3%**	**-26.8%**	**-35.0%**
20 Rheumatoid arthritis	20 Nasopharynx cancer	9.5%	-10.8%	**-22.2%**	20 Atrial fibrillation	**11.0%**	-1.7%	**-14.7%**
21 Nasopharynx cancer	21 Rheumatoid arthritis	**5.3%**	**-14.2%**	**-24.1%**	21 Bladder cancer	**11.4%**	-1.3%	**-14.1%**
22 Falls	22 Atrial fibrillation	**12.1%**	**-8.7%**	**-20.9%**	22 Rheumatoid arthritis	**4.7%**	**-7.3%**	**-16.6%**
23 Atrial fibrillation	23 Bladder cancer	**36.2%**	**11.0%**	-2.8%	23 Nasopharynx cancer	-17.2%	**-26.7%**	**-33.2%**
24 Bladder cancer	24 Falls	-1.5%	**-19.7%**	**-29.0%**	24 Interstitial lung disease	**24.8%**	**10.5%**	-2.8%
25 Aortic aneurysm	25 Interstitial lung disease	**96.6%**	**60.2%**	**41.5%**	25 Falls	**-5.6%**	**-16.4%**	**-26.0%**
26 Sense organ diseases	26 Sense organ diseases	**26.8%**	3.3%	**-10.7%**	26 Sense organ diseases	4.7%	**-7.3%**	**-19.7%**
27 Other chronic respiratory	27 Aortic aneurysm	**12.0%**	**-8.7%**	**-21.2%**	27 Aortic aneurysm	3.9%	**-8.0%**	**-18.5%**
28 Kidney cancer	28 Kidney cancer	**29.7%**	**5.7%**	**-6.9%**	28 Kidney cancer	**12.0%**	-0.8%	**-13.0%**
29 Leukemia	29 Leukemia	**31.1%**	6.8%	-6.2%	29 Leukemia	-1.6%	**-12.9%**	**-21.2%**
30 Road injuries	30 Other chronic respiratory	-4.9%	**-22.5%**	**-32.0%**	30 Other chronic respiratory	7.3%	-5.0%	-15.0%
31 Interstitial lung disease	31 Road injuries				32 Road injuries			

Legend:

Communicable, maternal, neonatal and nutritional

Non-communicable

Injuries

Figure S6a

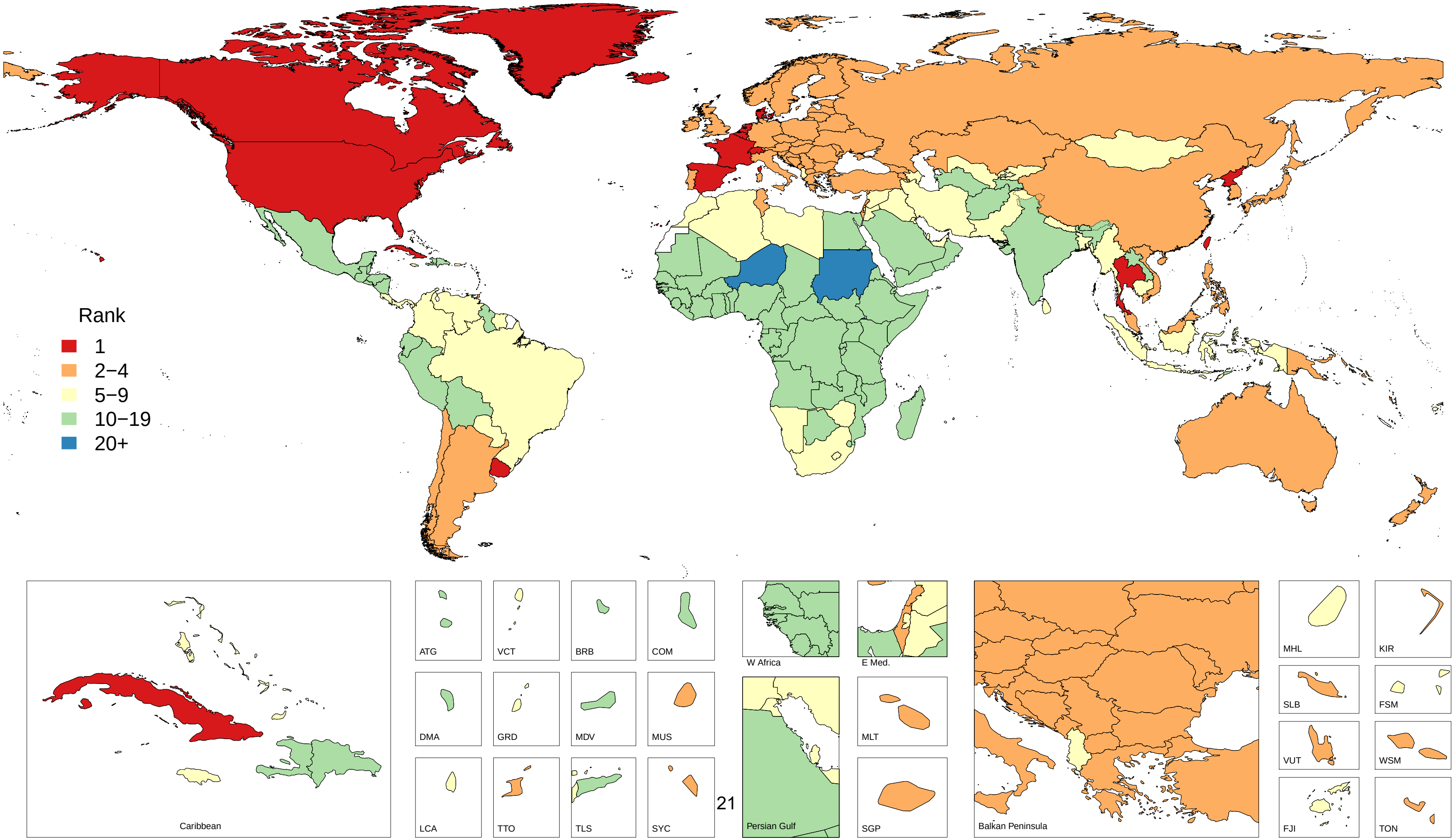
Rank
1
2–4
5–9
10–19
20+
Caribbean
ATG
VCT
BRB
COM
DMA
GRD
MDV
MUS
LCA
TTO
TLS
SYC
W Africa
E Med.
Persian Gulf
MLT
SGP
Balkan Peninsula
MHL
KIR
SLB
FSM
VUT
WSM
FJI
TON

Figure S6b

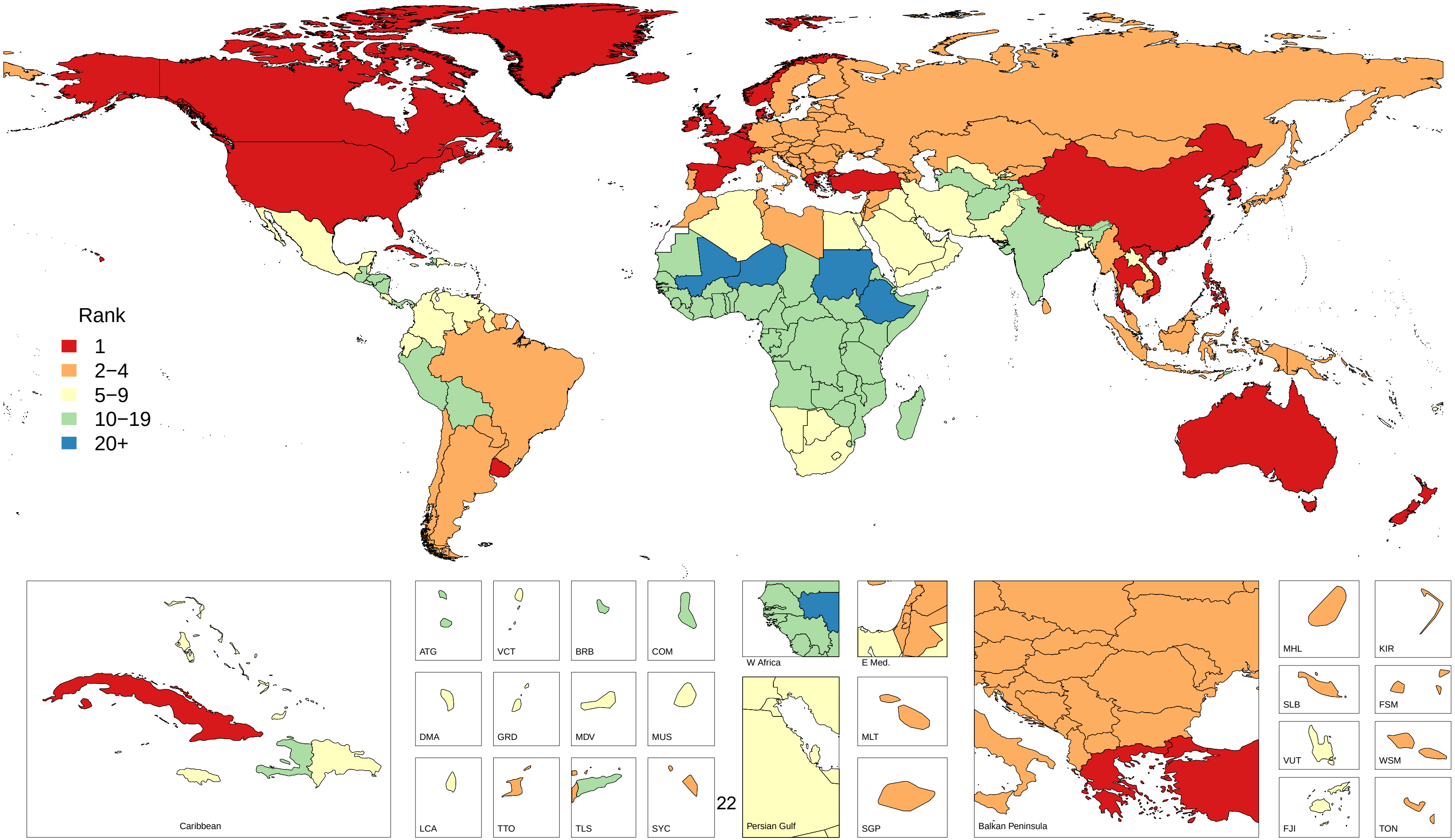
Rank
1
2–4
5–9
10–19
20+
Caribbean
ATG
VCT
BRB
COM
W Africa
E Med.
Balkan Peninsula
MHL
KIR
DMA
GRD
MDV
MUS
Persian Gulf
MLT
SLB
FSM
VUT
WSM
LCA
TTO
TLS
SYC
SGP
FJI
TON

Table S1

Location	SDI Group	SDI Value
Afghanistan	Low SDI	0.2888
Albania	High-middle SDI	0.7364
Algeria	Middle SDI	0.59
American Samoa	High-middle SDI	0.7136
Andorra	High SDI	0.9186
Angola	Low-middle SDI	0.4191
Antigua and Barbuda	High SDI	0.841
Argentina	High-middle SDI	0.772
Armenia	High-middle SDI	0.7552
Australia	High SDI	0.9151
Austria	High SDI	0.8883
Azerbaijan	High-middle SDI	0.7883
Bahrain	High-middle SDI	0.7764
Bangladesh	Low-middle SDI	0.4716
Barbados	High-middle SDI	0.7824
Belarus	High SDI	0.8468
Belgium	High SDI	0.8815
Belize	Middle SDI	0.6652
Benin	Low SDI	0.3446
Bermuda	High SDI	0.9164
Bhutan	Low-middle SDI	0.5321
Bolivia	Middle SDI	0.612
Bosnia and Herzegovina	High-middle SDI	0.7387
Botswana	Middle SDI	0.6411
Brazil	Middle SDI	0.6616
Brunei	High SDI	0.9232
Bulgaria	High-middle SDI	0.8079
Burkina Faso	Low SDI	0.2374
Burundi	Low SDI	0.2395
Cambodia	Low-middle SDI	0.4861
Cameroon	Low-middle SDI	0.4641
Canada	High SDI	0.9375
Cape Verde	Low-middle SDI	0.5485
Central African Republic	Low SDI	0.2817
Chad	Low SDI	0.2871
Chile	High-middle SDI	0.8048
China	Middle SDI	0.678
Colombia	High-middle SDI	0.6998
Comoros	Low SDI	0.365
Congo	Low-middle SDI	0.5265
Costa Rica	High-middle SDI	0.7232
Cote d'Ivoire	Low SDI	0.3807
Croatia	High-middle SDI	0.7842
Cuba	High-middle SDI	0.7662
Cyprus	High SDI	0.8805
Czech Republic	High SDI	0.8918

Democratic Republic of the Congo	Low SDI	0.2388
Denmark	High SDI	0.9096
Djibouti	Low-middle SDI	0.4615
Dominica	High-middle SDI	0.7526
Dominican Republic	High-middle SDI	0.6837
Ecuador	High-middle SDI	0.6852
Egypt	Middle SDI	0.6191
El Salvador	Middle SDI	0.6187
Equatorial Guinea	Middle SDI	0.6086
Eritrea	Low SDI	0.3238
Estonia	High SDI	0.8608
Ethiopia	Low SDI	0.3022
Federated States of Micronesia	Middle SDI	0.6242
Fiji	High-middle SDI	0.6934
Finland	High SDI	0.8929
France	High SDI	0.8343
Gabon	Middle SDI	0.6435
Georgia	High-middle SDI	0.7611
Germany	High SDI	0.9026
Ghana	Low-middle SDI	0.511
Greece	High-middle SDI	0.8246
Greenland	High-middle SDI	0.7575
Grenada	High-middle SDI	0.7532
Guam	High SDI	0.8841
Guatemala	Low-middle SDI	0.5427
Guinea	Low SDI	0.2777
Guinea-Bissau	Low SDI	0.2943
Guyana	Middle SDI	0.6546
Haiti	Low-middle SDI	0.4118
Honduras	Middle SDI	0.5684
Hungary	High SDI	0.8491
Iceland	High SDI	0.8933
India	Low-middle SDI	0.5564
Indonesia	Middle SDI	0.6523
Iran	High-middle SDI	0.7154
Iraq	Middle SDI	0.5756
Ireland	High SDI	0.8852
Israel	High SDI	0.8424
Italy	High SDI	0.8559
Jamaica	High-middle SDI	0.7189
Japan	High SDI	0.8955
Jordan	High-middle SDI	0.6949
Kazakhstan	High-middle SDI	0.8067
Kenya	Low SDI	0.4716
Kiribati	Low-middle SDI	0.4778
Kuwait	High SDI	0.8624
Kyrgyzstan	Middle SDI	0.6307

Laos	Low-middle SDI	0.5077
Latvia	High SDI	0.8614
Lebanon	High-middle SDI	0.7547
Lesotho	Low-middle SDI	0.5217
Liberia	Low SDI	0.2827
Libya	Middle SDI	0.643
Lithuania	High SDI	0.8369
Luxembourg	High SDI	0.9111
Macedonia	High-middle SDI	0.7622
Madagascar	Low SDI	0.3698
Malawi	Low SDI	0.3086
Malaysia	High-middle SDI	0.7669
Maldives	Middle SDI	0.6229
Mali	Low SDI	0.2309
Malta	High-middle SDI	0.8062
Marshall Islands	Middle SDI	0.5916
Mauritania	Low SDI	0.4014
Mauritius	High-middle SDI	0.7353
Mexico	Middle SDI	0.7176
Moldova	High-middle SDI	0.7033
Mongolia	High-middle SDI	0.7047
Montenegro	High-middle SDI	0.7989
Morocco	Low-middle SDI	0.4959
Mozambique	Low SDI	0.278
Myanmar	Low-middle SDI	0.52
Namibia	Middle SDI	0.617
Nepal	Low-middle SDI	0.4227
Netherlands	High SDI	0.8936
New Zealand	High SDI	0.8842
Nicaragua	Middle SDI	0.5631
Niger	Low SDI	0.1465
Nigeria	Low-middle SDI	0.474
North Korea	Middle SDI	0.5652
Northern Mariana Islands	High SDI	0.8406
Norway	High SDI	0.9365
Oman	High-middle SDI	0.7301
Pakistan	Low-middle SDI	0.4676
Palestine	Middle SDI	0.567
Panama	High-middle SDI	0.7467
Papua New Guinea	Low-middle SDI	0.4481
Paraguay	Middle SDI	0.6436
Peru	High-middle SDI	0.705
Philippines	Middle SDI	0.6454
Poland	High SDI	0.8675
Portugal	High-middle SDI	0.7522
Puerto Rico	High SDI	0.882
Qatar	High-middle SDI	0.8045

Romania	High-middle SDI	0.7991
Russia	High SDI	0.8564
Rwanda	Low SDI	0.3713
Saint Lucia	High-middle SDI	0.7408
Saint Vincent and the Grenadines	High-middle SDI	0.7473
Samoa	Middle SDI	0.6367
Sao Tome and Principe	Low-middle SDI	0.4481
Saudi Arabia	High-middle SDI	0.7593
Senegal	Low SDI	0.3341
Serbia	High-middle SDI	0.7718
Seychelles	High-middle SDI	0.7585
Sierra Leone	Low SDI	0.323
Singapore	High SDI	0.8811
Slovakia	High SDI	0.8615
Slovenia	High SDI	0.8561
Solomon Islands	Low-middle SDI	0.4614
Somalia	Low SDI	0.1506
South Africa	Middle SDI	0.7164
South Korea	High SDI	0.8713
South Sudan	Low SDI	0.2616
Spain	High-middle SDI	0.8192
Sri Lanka	High-middle SDI	0.7054
Sudan	Low-middle SDI	0.4282
Suriname	High-middle SDI	0.7037
Swaziland	Middle SDI	0.6232
Sweden	High SDI	0.8921
Switzerland	High SDI	0.9282
Syria	Middle SDI	0.579
Taiwan	High SDI	0.8648
Tajikistan	Middle SDI	0.5741
Tanzania	Low-middle SDI	0.4114
Thailand	High-middle SDI	0.705
The Bahamas	High SDI	0.8348
The Gambia	Low SDI	0.3266
Timor-Leste	Low-middle SDI	0.4499
Togo	Low SDI	0.3617
Tonga	Middle SDI	0.6224
Trinidad and Tobago	High SDI	0.8327
Tunisia	Middle SDI	0.6515
Turkey	High-middle SDI	0.69
Turkmenistan	High-middle SDI	0.7805
Uganda	Low SDI	0.3768
Ukraine	High-middle SDI	0.8111
United Arab Emirates	High SDI	0.8747
United Kingdom	High SDI	0.893
United States	High SDI	0.9311
Uruguay	High-middle SDI	0.745

Uzbekistan	High-middle SDI	0.6992
Vanuatu	Low-middle SDI	0.536
Venezuela	High-middle SDI	0.7283
Vietnam	Middle SDI	0.6283
Virgin Islands, U.S.	High SDI	0.8861
Yemen	Low-middle SDI	0.408
Zambia	Low-middle SDI	0.467
Zimbabwe	Low-middle SDI	0.5384

Table S2

Relative Risk Citations
Anda RF, Williamson DF, Escobedo LG, Remington PL. Smoking and the risk of peptic ulcer disease among women in the United States. Arch Intern Med. 1990; 150(7): 1437–41.
Buch H, Vinding T, la Cour M, Jensen GB, Prause JU, Nielsen NV. Risk factors for age-related maculopathy in a 14-year follow-up study: the Copenhagen City Eye Study. Acta Ophthalmol Scand. 2005; 83(4): 409-18 as it appears in Chakravarthy U, Wong TY, Fletcher A, Piault E, Evans C, Zlateva G, Buggage R, Pleil A, Mitchell P. Clinical risk
Christen WG, Glynn RJ, Ajani UA, Schaumberg DA, Buring JE, Hennekens CH, Manson JE. Smoking cessation and risk of age-related cataract in men. JAMA. 2000; 284(6): 713-6 as it appears in Ye J, He J, Wang C, Wu H, Shi X, Zhang H, Xie J, Lee SY. Smoking and risk of age-related cataract: a meta-analysis. Invest Ophthalmol Vis Sci. 2012;
Christen WG, Glynn RJ, Manson JE, Ajani UA, Buring JE. A prospective study of cigarette smoking and risk of age-related macular degeneration in men. JAMA. 1996; 276(14): 1147-51 as it appears in Chakravarthy U, Wong TY, Fletcher A, Piault E, Evans C, Zlateva G, Buggage R, Pleil A, Mitchell P. Clinical risk factors for age-related macular
Costenbader KH, Feskanich D, Mandl LA, Karlson EW. Smoking Intensity, Duration, and Cessation, and the Risk of Rheumatoid Arthritis in Women. Am J Med. 2006; 119(6): 503e1-9 as it appears in Sugiyama D, Nishimura K, Tamaki K, Tsuji G, Nakazawa T, Morinobu A, Kumagai S. Impact of smoking as a risk factor for developing
Criswell LA, Merlino LA, Cerhan JR, Mikuls TR, Mudano AS, Burma M, Folsom AR, Saag KG. Cigarette Smoking and the Risk of Rheumatoid Arthritis among Postmenopausal Women: Results from the Iowa Women's Health Study. Am J Med. 2002; 112(6): 465-71 as it appears in Sugiyama D, Nishimura K, Tamaki K, Tsuji G, Nakazawa T, Morinobu A, Kumagai S. Impact of smoking as a risk factor for developing rheumatoid arthritis: a meta-analysis of
Delcourt C, Carrière I, Delage M, Descomps B, Cristol JP, Papoz L. Associations of cataract with antioxidant enzymes and other risk factors: the French Age-Related Eye Diseases (POLA) Prospective Study. Ophthalmology. 2003; 110(12): 2318-26 as it appears in Ye J, He J, Wang C, Wu H, Shi X, Zhang H, Xie J, Lee SY. Smoking and risk of
European Prospective Osteoporosis Study (EPOS) Group, Felsenberg D, Silman AJ, Lunt M, Armbrecht G, Ismail AA, Finn JD, Cockerill WC, Banzer D, Benevolenskaya LI, Bhalla A, Bruges Armas J, Cannata JB, Cooper C, Dequeker J, Eastell R, Felsch B, Gowin W, Havelka S, Hoszowski K, Jajic I, Janott J, Johnell O, Kanis JA, Kragl G, Lopes Vaz A, Lorenc R, Lyritis G, Masaryk P, Matthis C, Miazgowski T, Parisi G, Pols H a. P, Poor G, Raspe HH, Reid DM, Reisinger W, Schedit-Nave C, Stepan JJ, Todd CJ, Weber K, Woolf AD, Yershova OB, Reeve J, O'Neill TW. Incidence of vertebral fracture in europe: results from the European Prospective Osteoporosis Study (EPOS). J Bone Miner Res.
European Prospective Osteoporosis Study (EPOS) Group, Felsenberg D, Silman AJ, Lunt M, Armbrecht G, Ismail AA, Finn JD, Cockerill WC, Banzer D, Benevolenskaya LI, Bhalla A, Bruges Armas J, Cannata JB, Cooper C, Dequeker J, Eastell R, Felsch B, Gowin W, Havelka S, Hoszowski K, Jajic I, Janott J, Johnell O, Kanis JA, Kragl G, Lopes Vaz A, Lorenc R, Lyritis G, Masaryk P, Matthis C, Miazgowski T, Parisi G, Pols H a. P, Poor G, Raspe HH, Reid DM, Reisinger W, Schedit-Nave C, Stepan JJ, Todd CJ, Weber K, Woolf AD, Yershova OB, Reeve J, O'Neill TW. Incidence of vertebral fracture in europe: results from the European Prospective Osteoporosis Study (EPOS). J Bone Miner Res.
Fujiwara S, Kasagi F, Yamada M, Kodama K. Risk factors for hip fracture in a Japanese cohort. J Bone Miner Res. 1997; 12(7): 998-1004 as it appears in Vestergaard P, Mosekilde L. Fracture risk associated with smoking: a meta-
Heliövaara M, Aho K, Aromaa A, Knekt P, Reunanen A. Smoking and risk of rheumatoid arthritis. J Rheumatol. 1993; 20(11): 1830-5 as it appears in Sugiyama D, Nishimura K, Tamaki K, Tsuji G, Nakazawa T, Morinobu A, Kumagai S. Impact of smoking as a risk factor for developing rheumatoid arthritis: a meta-analysis of
Hiller R, Sperduto RD, Podgor MJ, Wilson PW, Ferris FL 3rd, Colton T, D'Agostino RB, Roseman MJ, Stockman ME, Milton RC. Cigarette smoking and the risk of development of lens opacities. The Framingham studies. Arch Ophthalmol. 1997; 115(9): 1113-8 as it appears in Ye J, He J, Wang C, Wu H, Shi X, Zhang H, Xie J, Lee SY. Smoking
Hofman A, Grobbee DE, de Jong PT, van den Ouweland FA. Determinants of disease and disability in the elderly: The Rotterdam Elderly Study. Eur J Epidemiol. 1991; 7(4): 403-22 as it appears in Vestergaard P, Mosekilde L. Fracture risk associated with smoking: a meta-analysis. J Intern Med. 2003; 254(6): 572-83.

Relative Risk Citations
Hofman A, Grobbee DE, de Jong PT, van den Ouweland FA. Determinants of disease and disability in the elderly: The Rotterdam Elderly Study. Eur J Epidemiol. 1991; 7(4): 403-22 as it appears in Vestergaard P, Mosekilde L. Fracture risk associated with smoking: a meta-analysis. J Intern Med. 2003; 254(6): 572-83.
Honkanen R, Kröger H, Tuppurainen M, Alhava E, Saarikoski S. Fractures and low axial bone density in perimenopausal women. J Clin Epidemiol. 1995; 48(7): 881-8 as it appears in Vestergaard P, Mosekilde L. Fracture
Hozawa A, Houston T, Steffes MW, Widome R, Williams OD, Iribarren C, Pletcher MJ, Daviglus ML, Carr JJ, Jacobs DR. The association of cigarette smoking with self-reported disease before middle age: the Coronary Artery Risk
Johansson H, Oden A, Johnell O, Jonsson B, De Laet C, Oglesby A, McCloskey EV, Kayan J, Jalava T, Kanis JA. Optimization of BMD measurements to identify high risk groups for treatment--a test analysis. J Bone Miner Res. 2004; 19(6): 906–13 as it appears in Vestergaard P, Mosekilde L. Fracture risk associated with smoking: a meta-
Johansson H, Oden A, Johnell O, Jonsson B, De Laet C, Oglesby A, McCloskey EV, Kayan J, Jalava T, Kanis JA. Optimization of BMD measurements to identify high risk groups for treatment--a test analysis. J Bone Miner Res. 2004; 19(6): 906–13 as it appears in Vestergaard P, Mosekilde L. Fracture risk associated with smoking: a meta-
Jones G, Nguyen TV, Sambrook PN, Kelly PJ, Gilbert C, Eisman JA. Symptomatic fracture incidence in elderly men and women: the Dubbo Osteoporosis Epidemiology Study (DOES). Osteoporos Int. 1994; 4(5): 277–82 as it appears in Vestergaard P, Mosekilde L. Fracture risk associated with smoking: a meta-analysis. J Intern Med. 2003;
Karlson EW, Lee IM, Cook NR, Manson JE, Buring JE, Hennekens CH. A retrospective cohort study of cigarette smoking and risk of rheumatoid arthritis in female health professionals. Arthritis Rheum. 1999; 45(5): 910-7 as it appears in Sugiyama D, Nishimura K, Tamaki K, Tsuji G, Nakazawa T, Morinobu A, Kumagai S. Impact of smoking as a risk factor for developing rheumatoid arthritis: a meta-analysis of observational studies. Ann Rheum Dis.
Klein BE, Klein R, Lee KE, Meuer SM. Socioeconomic and Lifestyle Factors and the 10-year Incidence of Age-related Cataracts. Am J Ophthalmol. 2003; 136(3): 506-12 as it appears in Ye J, He J, Wang C, Wu H, Shi X, Zhang H, Xie J, Lee SY. Smoking and risk of age-related cataract: a meta-analysis. Invest Ophthalmol Vis Sci. 2012; 53(7): 3885-95.
Klein R, Klein BE, Linton KL. Prevalence of age-related maculopathy. The Beaver Dam Eye Study. Ophthalmology. 1992; 99(6): 933-43 as it appears in Chakravarthy U, Wong TY, Fletcher A, Piault E, Evans C, Zlateva G, Buggage R, Pleil A, Mitchell P. Clinical risk factors for age-related macular degeneration: a systematic review and meta-
Kreiger N, Tenenhouse A, Joseph L, Mackenzie T, Poliquin S, Brown JP, Prior JC, Rittmaster RS. Research Notes: The Canadian Multicentre Osteoporosis Study (CaMos): Background, Rationale, Methods. Can J Aging. 1999; 18(3): 376–87 as it appears in Vestergaard P, Mosekilde L. Fracture risk associated with smoking: a meta-analysis.
Kreiger N, Tenenhouse A, Joseph L, Mackenzie T, Poliquin S, Brown JP, Prior JC, Rittmaster RS. Research Notes: The Canadian Multicentre Osteoporosis Study (CaMos): Background, Rationale, Methods. Can J Aging. 1999; 18(3): 376–87 as it appears in Vestergaard P, Mosekilde L. Fracture risk associated with smoking: a meta-analysis.
Kurata JH, Nogawa AN, Abbey DE, Petersen F. A prospective study of risk for peptic ulcer disease in Seventh-Day Adventists. Gastroenterology. 1992; 102(3): 902–9.
Leske MC, Chylack LT Jr, He Q, Wu SY, Schoenfeld E, Friend J, Wolfe J. Risk factors for nuclear opalescence in a longitudinal study. Am J Epidemiol. 1998; 147(1): 36-41 as it appears in Ye J, He J, Wang C, Wu H, Shi X, Zhang H, Xie J, Lee SY. Smoking and risk of age-related cataract: a meta-analysis. Invest Ophthalmol Vis Sci. 2012; 53(7):
Leske MC, Wu SY, Hennis A, Nemesure B, Yang L, Hyman L, Schachat AP; Barbados Eye Studies Group. Nine-year incidence of age-related macular degeneration in the Barbados Eye Studies. Ophthalmology. 2006; 113(1): 29-35 as it appears in Chakravarthy U, Wong TY, Fletcher A, Piault E, Evans C, Zlateva G, Buggage R, Pleil A, Mitchell P. Clinical risk factors for age-related macular degeneration: a systematic review and meta-analysis. BMC
Lindblad BE, Håkansson N, Svensson H, Philipson B, Wolk A. Intensity of Smoking and Smoking Cessation in Relation to Risk of Cataract Extraction: A Prospective Study of Women. Am J Epidemiol. 2005; 162(1): 73-9 as it appears in Ye J, He J, Wang C, Wu H, Shi X, Zhang H, Xie J, Lee SY. Smoking and risk of age-related cataract: a meta-

Relative Risk Citations
Melton LJ, Crowson CS, O'Fallon WM, Wahner HW, Riggs BL. Relative contributions of bone density, bone turnover, and clinical risk factors to long-term fracture prediction. J Bone Miner Res. 2003; 18(2): 312–8 as it appears in Vestergaard P, Mosekilde L. Fracture risk associated with smoking: a meta-analysis. J Intern Med. 2003;
Melton LJ, Crowson CS, O'Fallon WM, Wahner HW, Riggs BL. Relative contributions of bone density, bone turnover, and clinical risk factors to long-term fracture prediction. J Bone Miner Res. 2003; 18(2): 312–8 as it appears in Vestergaard P, Mosekilde L. Fracture risk associated with smoking: a meta-analysis. J Intern Med. 2003;
Mukesh BN, Le A, Dimitrov PN, Ahmed S, Taylor HR, McCarty CA. Development of cataract and associated risk factors: the Visual Impairment Project. Arch Ophthalmol. 2006; 124(1): 79-85 as it appears in Ye J, He J, Wang C, Wu H, Shi X, Zhang H, Xie J, Lee SY. Smoking and risk of age-related cataract: a meta-analysis. Invest Ophthalmol
Oza S, Thun MJ, Henley SJ, Lopez AD, Ezzati M. How many deaths are attributable to smoking in the United States? Comparison of methods for estimating smoking-attributable mortality when smoking prevalence changes.
Paffenbarger RS, Wing AL, Hyde RT. Chronic disease in former college students; 13. Early precursors of peptic
Schoon IM, Mellstrom D, Oden A, Ytterberg BO. Peptic ulcer disease in older age groups in Gothenburg in 1985: the association with smoking. Age Ageing. 1991; 20(5): 371–6.
Stenstrom M, Olsson JO, Mellstrom D. Thyroid hormone replacement is not related to increased risk of osteoporosis. Osteoporos Int. 2000; 11(Suppl. 2): S144 as it appears in Vestergaard P, Mosekilde L. Fracture risk
Stenstrom M, Olsson JO, Mellstrom D. Thyroid hormone replacement is not related to increased risk of osteoporosis. Osteoporos Int. 2000; 11(Suppl. 2): S144 as it appears in Vestergaard P, Mosekilde L. Fracture risk
Svanborg A. Seventy-year-old people in Gothenburg a population study in an industrialized Swedish city. II. General presentation of social and medical conditions. Acta Med Scand. 1977; 611(Suppl 5): 5-37 as it appears in Vestergaard P, Mosekilde L. Fracture risk associated with smoking: a meta-analysis. J Intern Med. 2003; 254(6):
Tan JS, Wang JJ, Younan C, Cumming RG, Rochtchina E, Mitchell P. Smoking and the long-term incidence of cataract: the Blue Mountains Eye Study. Ophthalmic Epidemiol. 2008; 15(3): 155-61 as it appears in Ye J, He J, Wang C, Wu H, Shi X, Zhang H, Xie J, Lee SY. Smoking and risk of age-related cataract: a meta-analysis. Invest
Tomany SC, Wang JJ, Van Leeuwen R, Klein R, Mitchell P, Vingerling JR, Klein BE, Smith W, De Jong PT. Risk factors for incident age-related macular degeneration: pooled findings from 3 continents. Ophthalmology. 2004; 111(7): 1280-7 as it appears in Chakravarthy U, Wong TY, Fletcher A, Piault E, Evans C, Zlateva G, Buggage R, Pleil A, Mitchell P. Clinical risk factors for age-related macular degeneration: a systematic review and meta-analysis. BMC
Vessey MP, Villard-Mackintosh L, Yeates D. Oral Contraceptives, cigarette smoking and other factors in relation to arthritis. Contraception. 1987; 35(5): 457-64 as it appears in Sugiyama D, Nishimura K, Tamaki K, Tsuji G, Nakazawa T, Morinobu A, Kumagai S. Impact of smoking as a risk factor for developing rheumatoid arthritis: a
Weintraub JM, Willett WC, Rosner B, Colditz GA, Seddon JM, Hankinson SE. Smoking Cessation and Risk of Cataract Extraction among US Women and Men. Am J Epidemiol. 2002; 155(1): 72-9 as it appears in Ye J, He J, Wang C, Wu H, Shi X, Zhang H, Xie J, Lee SY. Smoking and risk of age-related cataract: a meta-analysis. Invest
West S, Muñoz B, Schein OD, Vitale S, Maguire M, Taylor HR, Bressler NM. Cigarette smoking and risk for progression of nuclear opacities. Arch Ophthalmol. 1995; 113(11): 1377-80 as it appears in Ye J, He J, Wang C, Wu H, Shi X, Zhang H, Xie J, Lee SY. Smoking and risk of age-related cataract: a meta-analysis. Invest Ophthalmol Vis
West S, Muñoz B, Schein OD, Vitale S, Maguire M, Taylor HR, Bressler NM. Cigarette smoking and risk for progression of nuclear opacities. Arch Ophthalmol. 1995; 113(11): 1377-80 as it appears in Ye J, He J, Wang C, Wu H, Shi X, Zhang H, Xie J, Lee SY. Smoking and risk of age-related cataract: a meta-analysis. Invest Ophthalmol Vis

Table S3	Daily	Current	Occasional	Number of Data Points
**Smoked Tobacco**	59250	19692	6717	85659
**Cigarettes**	13636	20378	9342	43356
**Manufactured Cigarettes**	5036	7375	4286	16697
**Hand-Rolled Cigarettes**	4865	8045	4620	17530
**All Tobacco**	2793	7775	1225	11793
**Smoked Tobacco Excluding Cigarettes**	2231	11696	2146	16073
**Smokeless Tobacco**	1612	5566	1167	8345
**All Tobacco Excluding Cigarettes**	221	623	188	1032
**Number of Data Points**	89644	81150	29691	200485

Table S4

Frequency-Type Combination	Adjustment (95% CI)	Adjusted R-Squared	Number of Data Points
**Daily - Smoked Tobacco**	-	-	59,250
**Current - Smoked Tobacco**	0.831 (0.826, 0.836)	0.971	13,658
**Daily - Cigarettes**	1.077 (1.052, 1.102)	0.918	7,097
**Current - All Tobacco**	0.659 (0.642, 0.675)	0.855	2,096
**Daily - All Tobacco**	0.742 (0.723, 0.760)	0.853	562
**Daily - Manufactured Cigarettes**	1.132 (1.089, 1.175)	0.849	11
**Current***	0.852 (0.849, 0.855)	0.972	5,439
**Cigarettes***	1.107 (1.090, 1.124)	0.854	5,493

Table S5

Outcome	Exposure	Prospective observational studies (n)	Prospective observational studies with significant association in the opposite direction (%)	Lower limit of RR > 1.5	Biologic plausibility	Analogy
Tuberculosis	5-year lagged prevalence	4	0	🔴	🟢	🟢
Lower respiratory infections[1]	5-year lagged prevalence	0	..	🔴	🟢	🟢
Lip and oral cavity cancer	Smoking impact ratio	3	0	🟢	🟢	🟢
Nasopharynx cancer[2]	Smoking impact ratio	3	0	🟢	🟢	🟢
Oesophageal cancer	Smoking impact ratio	5	0	🟢	🟢	🟢
Larynx cancer	Smoking impact ratio	4	0	🟢	🟢	🟢
Stomach cancer	Smoking impact ratio	9	0	🔴	🟢	🟢
Colon and rectum cancer	Smoking impact ratio	19	0	🔴	🟢	🟢
Liver cancer[3]	Smoking impact ratio	54	0	🟢	🟢	🟢
Pancreatic cancer	Smoking impact ratio	13	0	🟢	🟢	🟢
Tracheal, bronchus, and lung cancer	Smoking impact ratio	38	0	🟢	🟢	🟢
Cervical cancer	Smoking impact ratio	15	0	🔴	🟢	🟢
Kidney cancer	Smoking impact ratio	8	0	🟢	🟢	🟢
Bladder cancer[4]	Smoking impact ratio	17	0	🟢	🟢	🟢
Leukaemia	Smoking impact ratio	14	0	🔴	🟢	🟢
Ischaemic heart disease	5-year lagged prevalence	86	..	🔴	🟢	🟢
Ischemic stroke	5-year lagged prevalence	60	..	🔴	🟢	🟢
Hemorrhagic stroke	5-year lagged prevalence	60	..	🔴	🟢	🟢
Hypertensive heart disease	5-year lagged prevalence	5	..	🔴	🟢	🟢
Atrial fibrillation and flutter	5-year lagged prevalence	16	0	🔴	🟢	🟢
Aortic aneurysm	5-year lagged prevalence	10	0	🔴	🟢	🟢
Peripheral vascular disease	5-year lagged prevalence	10	0	🔴	🟢	🟢
Other cardiovascular and circulatory diseases	5-year lagged prevalence	1	0	🔴	🟢	🟢
Chronic obstructive pulmonary disease	Smoking impact ratio	42	0	🟢	🟢	🟢
Silicosis	Smoking impact ratio	0	..	🔴	🟢	🟢
Asbestosis[5]	Smoking impact ratio	0	..	🔴	🟢	🟢
Coal workers pneumoconiosis	Smoking impact ratio	0	..	🟢	🟢	🟢
Other pneumoconiosis	Smoking impact ratio	0	..	🟢	🟢	🟢
Asthma	5-year lagged prevalence	6	0	🔴	🟢	🟢
Interstitial lung disease and pulmonary sarcoidosis	Smoking impact ratio	0	..	🟢	🟢	🟢
Other chronic respiratory diseases	Smoking impact ratio	1	0	🟢	🟢	🟢
Peptic ulcer disease	5-year lagged prevalence	7	0	🔴	🟢	🔴
Diabetes mellitus	5-year lagged prevalence	51	0	🔴	🟢	🔴

Outcome	Exposure	Prospective observational studies (n)	Prospective observational studies with significant association in the opposite direction (%)	Lower limit of RR > 1.5	Biologic plausibility	Analogy
Cataract	5-year lagged prevalence	10	0	No	Yes	No
Macular degeneration	5-year lagged prevalence	5	20	No	Yes	No
Rheumatoid arthritis	5-year lagged prevalence	5	0	No	Yes	No
Hip fracture	5-year lagged prevalence	15	20	No	Yes	Yes
Non-hip fracture	5-year lagged prevalence	14	14	No	Yes	Yes

Yes

No

Footnotes

1 Evidence on the relationship between smoking and lower respiratory infections comes 10 case-control or cross-sectional studies

2 The evidence on causal relationship of alcohol and smoking with nasopharynx cancer was from the studies evaluating oral cavity and pharyngeal cancers as outcome

3 Liver cancer included liver cancer due to alcohol use, hepatitis B, hepatitis C, and other causes

4 The evidence on causal relationship of smoking and bladder cancer was based on the studies evaluating the lower urinary tract as outcome

5 Asbestosis, coal workers pneumoconiosis, other pneumoconiosis, silicosis were included as outcomes for smoking as they were included in the other chronic respiratory diseases category

Table S6

Outcome	Citation
**Tuberculosis**	Bates MN, Khalakdina A, Pai M, Chang L, Lessa F, Smith KR. Risk of tuberculosis from exposure to tobacco smoke: a systematic review and meta-analysis. Arch Intern Med 2007; 167: 335–42.
**Tuberculosis**	Lin H-H, Ezzati M, Murray M. Tobacco smoke, indoor air pollution and tuberculosis: a systematic review and meta-analysis. PLoS Med 2007; 4: e20.
**Tuberculosis**	Slama K, Chiang C-Y, Enarson DA, et al. Tobacco and tuberculosis: a qualitative systematic review and meta-analysis. Int J Tuberc Lung Dis 2007; 11: 1049–61.
**Lower respiratory infections**	Surgeon General's Report - The Health Consequences of Smoking. U.S. Department of Health & Human Services, 2004 http://www.cdc.gov/tobacco/data_statistics/sgr/2004/.
**Lip and oral cavity cancer**	Surgeon General's Report - The Health Consequences of Smoking. U.S. Department of Health & Human Services, 2004 http://www.cdc.gov/tobacco/data_statistics/sgr/2004/.
**Nasopharynx cancer**	Surgeon General's Report - The Health Consequences of Smoking. U.S. Department of Health & Human Services, 2004 http://www.cdc.gov/tobacco/data_statistics/sgr/2004/.
**Oesophageal cancer**	Surgeon General's Report - The Health Consequences of Smoking. U.S. Department of Health & Human Services, 2004 http://www.cdc.gov/tobacco/data_statistics/sgr/2004/.
**Stomach cancer**	Surgeon General's Report - The Health Consequences of Smoking. U.S. Department of Health & Human Services, 2004 http://www.cdc.gov/tobacco/data_statistics/sgr/2004/.
**Colon and rectum cancer**	Surgeon General's Report - The Health Consequences of Smoking. U.S. Department of Health & Human Services, 2004 http://www.cdc.gov/tobacco/data_statistics/sgr/2004/.
**Liver cancer**	Surgeon General's Report - The Health Consequences of Smoking. U.S. Department of Health & Human Services, 2004 http://www.cdc.gov/tobacco/data_statistics/sgr/2004/.
**Pancreatic cancer**	Surgeon General's Report - The Health Consequences of Smoking. U.S. Department of Health & Human Services, 2004 http://www.cdc.gov/tobacco/data_statistics/sgr/2004/.
**Larynx cancer**	International Agency for Research on Cancer Working Group on the Evaluation of Carcinogenic Risks to Humans. IARC monographs on the evaluation of carcinogenic risks to humans: Tobacco Smoke and Involuntary Smoking. Lyon: IARC, 2004.
**Tracheal, bronchus and lung cancer**	International Agency for Research on Cancer Working Group on the Evaluation of Carcinogenic Risks to Humans. IARC monographs on the evaluation of carcinogenic risks to humans: Tobacco Smoke and Involuntary Smoking. Lyon: IARC, 2004.
**Cervical cancer**	International Agency for Research on Cancer Working Group on the Evaluation of Carcinogenic Risks to Humans. IARC monographs on the evaluation of carcinogenic risks to humans: Tobacco Smoke and Involuntary Smoking. Lyon: IARC, 2004.

**Kidney cancer**	International Agency for Research on Cancer Working Group on the Evaluation of Carcinogenic Risks to Humans. IARC monographs on the evaluation of carcinogenic risks to humans: Tobacco Smoke and Involuntary Smoking. Lyon: IARC, 2004.
**Bladder cancer**	International Agency for Research on Cancer Working Group on the Evaluation of Carcinogenic Risks to Humans. IARC monographs on the evaluation of carcinogenic risks to humans: Tobacco Smoke and Involuntary Smoking. Lyon: IARC, 2004.
**Leukaemia**	Surgeon General's Report - The Health Consequences of Smoking. U.S. Department of Health & Human Services, 2004 http://www.cdc.gov/tobacco/data_statistics/sgr/2004/.
**Ischaemic heart disease**	Huxley RR, Woodward M. Cigarette smoking as a risk factor for coronary heart disease in women compared with men: a systematic review and meta-analysis of prospective cohort studies. Lancet 2011; 378: 1297–305.
**Cerebrovascular disease**	Peters SAE, Huxley RR, Woodward M. Smoking as a risk factor for stroke in women compared with men: a systematic review and meta-analysis of 81 cohorts, including 3,980,359 individuals and 42,401 strokes. Stroke 2013; 44: 2821–8.
**Hypertensive heart disease**	Carter BD, Abnet CC, Feskanich D, et al. Smoking and mortality--beyond established causes. N Engl J Med 2015; 372: 631–40.
**Atrial fibrillation and flutter**	Zhu W, Yuan P, Shen Y, Wan R, Hong K. Association of smoking with the risk of incident atrial fibrillation: A meta-analysis of prospective studies. Int J Cardiol 2016; 218: 259–66.
**Aortic aneurysm**	Lederle FA, Nelson DB, Joseph AM. Smokers' relative risk for aortic aneurysm compared with other smoking-related diseases: a systematic review. J Vasc Surg 2003; 38: 329–34.
**Peripheral vascular disease**	Lu L, Mackay DF, Pell JP. Meta-analysis of the association between cigarette smoking and peripheral arterial disease. Heart 2014; 100: 414–23.
**Other cardiovascular and circulatory diseases**	Carter BD, Abnet CC, Feskanich D, et al. Smoking and mortality--beyond established causes. N Engl J Med 2015; 372: 631–40.
**Chronic obstructive pulmonary disease**	Forey BA, Thornton AJ, Lee PN. Systematic review with meta-analysis of the epidemiological evidence relating smoking to COPD, chronic bronchitis and emphysema. BMC Pulm Med 2011; 11: 36.
**Asthma**	The Health Consequences of Smoking—50 Years of Progress. U.S. Department of Health & Human Services, 2014 http://www.surgeongeneral.gov/library/reports/50-years-of-progress/full-report.pdf.
**Other chronic respiratory diseases**	Carter BD, Abnet CC, Feskanich D, et al. Smoking and mortality--beyond established causes. N Engl J Med 2015; 372: 631–40.

**Peptic ulcer disease**	Kurata JH, Nogawa AN. Meta-analysis of risk factors for peptic ulcer. Nonsteroidal antiinflammatory drugs, Helicobacter pylori, and smoking. J Clin Gastroenterol 1997; 24: 2–17.
**Diabetes mellitus**	The Health Consequences of Smoking—50 Years of Progress. U.S. Department of Health & Human Services, 2014 http://www.surgeongeneral.gov/library/reports/50-years-of-progress/full-report.pdf.
**Rheumatoid arthritis**	Sugiyama D, Nishimura K, Tamaki K, et al. Impact of smoking as a risk factor for developing rheumatoid arthritis: a meta-analysis of observational studies. Ann Rheum Dis 2010; 69: 70–81.
**Cataract**	Ye J, He J, Wang C, et al. Smoking and risk of age-related cataract: a meta-analysis. Invest Ophthalmol Vis Sci 2012; 53: 3885–95.
**Macular degeneration**	Chakravarthy U, Wong TY, Fletcher A, et al. Clinical risk factors for age-related macular degeneration: a systematic review and meta-analysis. BMC Ophthalmol 2010; 10: 31.
**Hip fracture**	Vestergaard P, Mosekilde L. Fracture risk associated with smoking: a meta-analysis. J Intern Med 2003; 254: 572–83.
**Non-hip fracture**	Vestergaard P, Mosekilde L. Fracture risk associated with smoking: a meta-analysis. J Intern Med 2003; 254: 572–83.

Table S7

	Sex	30-34 years	35-39 years	40-44 years	45-49 years	50-54 years	55-59 years	60-64 years
Tuberculosis	Male	1·588 (1·242 to 2·039)	1·588 (1·242 to 2·039)	1·588 (1·242 to 2·039)	1·588 (1·242 to 2·039)	1·588 (1·242 to 2·039)	1·588 (1·242 to 2·039)	1·588 (1·242 to 2·039)
Tuberculosis	Female	1·599 (1·258 to 2·024)	1·599 (1·258 to 2·024)	1·599 (1·258 to 2·024)	1·599 (1·258 to 2·024)	1·599 (1·258 to 2·024)	1·599 (1·258 to 2·024)	1·599 (1·258 to 2·024)
Ischaemic heart disease	Male	4·316 (3·127 to 5·810)	3·924 (2·905 to 5·186)	3·569 (2·699 to 4·630)	3·246 (2·508 to 4·133)	2·952 (2·330 to 3·689)	2·685 (2·165 to 3·293)	2·443 (2·011 to 2·940)
Ischaemic heart disease	Female	6·145 (5·060 to 7·413)	5·464 (4·557 to 6·515)	4·859 (4·105 to 5·725)	4·321 (3·697 to 5·031)	3·843 (3·330 to 4·421)	3·417 (2·999 to 3·885)	3·039 (2·701 to 3·414)
Ischaemic stroke	Male	4·175 (3·165 to 5·452)	3·805 (2·939 to 4·887)	3·468 (2·728 to 4·381)	3·161 (2·533 to 3·927)	2·882 (2·351 to 3·520)	2·627 (2·183 to 3·155)	2·395 (2·026 to 2·828)
Ischaemic stroke	Female	6·020 (4·248 to 8·410)	5·357 (3·869 to 7·331)	4·767 (3·525 to 6·390)	4·243 (3·211 to 5·569)	3·777 (2·925 to 4·855)	3·363 (2·664 to 4·231)	2·994 (2·427 to 3·688)
Hemorrhagic stroke	Male	4·175 (3·165 to 5·452)	3·805 (2·939 to 4·887)	3·468 (2·728 to 4·381)	3·161 (2·533 to 3·927)	2·882 (2·351 to 3·520)	2·627 (2·183 to 3·155)	2·395 (2·026 to 2·828)
Hemorrhagic stroke	Female	6·020 (4·248 to 8·410)	5·357 (3·869 to 7·331)	4·767 (3·525 to 6·390)	4·243 (3·211 to 5·569)	3·777 (2·925 to 4·855)	3·363 (2·664 to 4·231)	2·994 (2·427 to 3·688)
Hypertensive heart disease	Male	4·153 (2·995 to 5·659)	3·785 (2·790 to 5·061)	3·451 (2·600 to 4·525)	3·146 (2·422 to 4·046)	2·868 (2·257 to 3·618)	2·616 (2·102 to 3·236)	2·386 (1·959 to 2·893)
Hypertensive heart disease	Female	4·110 (2·053 to 7·209)	3·740 (1·960 to 6·346)	3·405 (1·871 to 5·587)	3·102 (1·786 to 4·919)	2·826 (1·705 to 4·330)	2·576 (1·628 to 3·812)	2·350 (1·554 to 3·356)
Atrial fibrillation and flutter	Male	4·153 (2·995 to 5·659)	3·785 (2·790 to 5·061)	3·451 (2·600 to 4·525)	3·146 (2·422 to 4·046)	2·868 (2·257 to 3·618)	2·616 (2·102 to 3·236)	2·386 (1·959 to 2·893)
Atrial fibrillation and flutter	Female	4·110 (2·053 to 7·209)	3·740 (1·960 to 6·346)	3·405 (1·871 to 5·587)	3·102 (1·786 to 4·919)	2·826 (1·705 to 4·330)	2·576 (1·628 to 3·812)	2·350 (1·554 to 3·356)
Aortic aneurysm	Male	4·153 (2·995 to 5·659)	3·785 (2·790 to 5·061)	3·451 (2·600 to 4·525)	3·146 (2·422 to 4·046)	2·868 (2·257 to 3·618)	2·616 (2·102 to 3·236)	2·386 (1·959 to 2·893)
Aortic aneurysm	Female	4·110 (2·053 to 7·209)	3·740 (1·960 to 6·346)	3·405 (1·871 to 5·587)	3·102 (1·786 to 4·919)	2·826 (1·705 to 4·330)	2·576 (1·628 to 3·812)	2·350 (1·554 to 3·356)
Peripheral vascular disease	Male	4·153 (2·995 to 5·659)	3·785 (2·790 to 5·061)	3·451 (2·600 to 4·525)	3·146 (2·422 to 4·046)	2·868 (2·257 to 3·618)	2·616 (2·102 to 3·236)	2·386 (1·959 to 2·893)
Peripheral vascular disease	Female	4·110 (2·053 to 7·209)	3·740 (1·960 to 6·346)	3·405 (1·871 to 5·587)	3·102 (1·786 to 4·919)	2·826 (1·705 to 4·330)	2·576 (1·628 to 3·812)	2·350 (1·554 to 3·356)
Other cardiovascular and circulatory diseases	Male	4·153 (2·995 to 5·659)	3·785 (2·790 to 5·061)	3·451 (2·600 to 4·525)	3·146 (2·422 to 4·046)	2·868 (2·257 to 3·618)	2·616 (2·102 to 3·236)	2·386 (1·959 to 2·893)
Other cardiovascular and circulatory diseases	Female	4·110 (2·053 to 7·209)	3·740 (1·960 to 6·346)	3·405 (1·871 to 5·587)	3·102 (1·786 to 4·919)	2·826 (1·705 to 4·330)	2·576 (1·628 to 3·812)	2·350 (1·554 to 3·356)
Asthma	Male	2·098 (1·761 to 2·460)	2·098 (1·761 to 2·460)	2·098 (1·761 to 2·460)	2·098 (1·761 to 2·460)	2·098 (1·761 to 2·460)	2·098 (1·761 to 2·460)	2·098 (1·761 to 2·460)
Asthma	Female	1·976 (1·788 to 2·181)	1·976 (1·788 to 2·181)	1·976 (1·788 to 2·181)	1·976 (1·788 to 2·181)	1·976 (1·788 to 2·181)	1·976 (1·788 to 2·181)	1·976 (1·788 to 2·181)
Peptic ulcer disease	Both	2·040 (1·684 to 2·483)	2·040 (1·684 to 2·483)	2·040 (1·684 to 2·483)	2·040 (1·684 to 2·483)	2·040 (1·684 to 2·483)	2·040 (1·684 to 2·483)	2·040 (1·684 to 2·483)
Diabetes mellitus	Male	1·426 (1·094 to 1·842)	1·426 (1·094 to 1·842)	1·426 (1·094 to 1·842)	1·426 (1·094 to 1·842)	1·426 (1·094 to 1·842)	1·426 (1·094 to 1·842)	1·426 (1·094 to 1·842)

	Sex	65-69 years	70-74 years	75-79 years	80+ years
Tuberculosis	Male	1·588 (1·242 to 2·039)	1·588 (1·242 to 2·039)	1·588 (1·242 to 2·039)	1·588 (1·242 to 2·039)
Tuberculosis	Female	1·599 (1·258 to 2·024)	1·599 (1·258 to 2·024)	1·599 (1·258 to 2·024)	1·599 (1·258 to 2·024)
Ischaemic heart disease	Male	2·223 (1·869 to 2·624)	2·023 (1·736 to 2·343)	1·841 (1·613 to 2·091)	1·598 (1·445 to 1·764)
Ischaemic heart disease	Female	2·703 (2·433 to 3·000)	2·404 (2·191 to 2·636)	2·139 (1·974 to 2·317)	1·794 (1·687 to 1·908)
Ischaemic stroke	Male	2·184 (1·881 to 2·535)	1·992 (1·746 to 2·272)	1·816 (1·621 to 2·036)	1·582 (1·450 to 1·728)
Ischaemic stroke	Female	2·666 (2·210 to 3·215)	2·375 (2·014 to 2·802)	2·115 (1·834 to 2·442)	1·778 (1·595 to 1·988)
Hemorrhagic stroke	Male	2·184 (1·881 to 2·535)	1·992 (1·746 to 2·272)	1·816 (1·621 to 2·036)	1·582 (1·450 to 1·728)
Hemorrhagic stroke	Female	2·666 (2·210 to 3·215)	2·375 (2·014 to 2·802)	2·115 (1·834 to 2·442)	1·778 (1·595 to 1·988)
Hypertensive heart disease	Male	2·176 (1·825 to 2·587)	1·985 (1·700 to 2·313)	1·811 (1·584 to 2·069)	1·578 (1·425 to 1·749)
Hypertensive heart disease	Female	2·144 (1·484 to 2·954)	1·957 (1·416 to 2·601)	1·787 (1·352 to 2·290)	1·560 (1·261 to 1·891)
Atrial fibrillation and flutter	Male	2·176 (1·825 to 2·587)	1·985 (1·700 to 2·313)	1·811 (1·584 to 2·069)	1·578 (1·425 to 1·749)
Atrial fibrillation and flutter	Female	2·144 (1·484 to 2·954)	1·957 (1·416 to 2·601)	1·787 (1·352 to 2·290)	1·560 (1·261 to 1·891)
Aortic aneurysm	Male	2·176 (1·825 to 2·587)	1·985 (1·700 to 2·313)	1·811 (1·584 to 2·069)	1·578 (1·425 to 1·749)
Aortic aneurysm	Female	2·144 (1·484 to 2·954)	1·957 (1·416 to 2·601)	1·787 (1·352 to 2·290)	1·560 (1·261 to 1·891)
Peripheral vascular disease	Male	2·176 (1·825 to 2·587)	1·985 (1·700 to 2·313)	1·811 (1·584 to 2·069)	1·578 (1·425 to 1·749)
Peripheral vascular disease	Female	2·144 (1·484 to 2·954)	1·957 (1·416 to 2·601)	1·787 (1·352 to 2·290)	1·560 (1·261 to 1·891)
Other cardiovascular and circulatory diseases	Male	2·176 (1·825 to 2·587)	1·985 (1·700 to 2·313)	1·811 (1·584 to 2·069)	1·578 (1·425 to 1·749)
Other cardiovascular and circulatory diseases	Female	2·144 (1·484 to 2·954)	1·957 (1·416 to 2·601)	1·787 (1·352 to 2·290)	1·560 (1·261 to 1·891)
Asthma	Male	2·098 (1·761 to 2·460)	2·098 (1·761 to 2·460)	2·098 (1·761 to 2·460)	2·098 (1·761 to 2·460)
Asthma	Female	1·976 (1·788 to 2·181)	1·976 (1·788 to 2·181)	1·976 (1·788 to 2·181)	1·976 (1·788 to 2·181)
Peptic ulcer disease	Both	2·040 (1·684 to 2·483)	2·040 (1·684 to 2·483)	2·040 (1·684 to 2·483)	2·040 (1·684 to 2·483)
Diabetes mellitus	Male	1·426 (1·094 to 1·842)	1·426 (1·094 to 1·842)	1·426 (1·094 to 1·842)	1·426 (1·094 to 1·842)

	Sex	30-34 years	35-39 years	40-44 years	45-49 years	50-54 years	55-59 years	60-64 years
Diabetes mellitus	Female	1·102 (0·953 to 1·275)	1·102 (0·953 to 1·275)	1·102 (0·953 to 1·275)	1·102 (0·953 to 1·275)	1·102 (0·953 to 1·275)	1·102 (0·953 to 1·275)	1·102 (0·953 to 1·275)
Rheumatoid arthritis	Both	1·375 (1·142 to 1·652)	1·375 (1·142 to 1·652)	1·375 (1·142 to 1·652)	1·375 (1·142 to 1·652)	1·375 (1·142 to 1·652)	1·375 (1·142 to 1·652)	1·375 (1·142 to 1·652)
Cataract	Both	1·671 (1·479 to 1·875)	1·671 (1·479 to 1·875)	1·671 (1·479 to 1·875)	1·671 (1·479 to 1·875)	1·671 (1·479 to 1·875)	1·671 (1·479 to 1·875)	1·671 (1·479 to 1·875)
Macular degeneration	Both	1·911 (1·265 to 2·740)	1·911 (1·265 to 2·740)	1·911 (1·265 to 2·740)	1·911 (1·265 to 2·740)	1·911 (1·265 to 2·740)	1·911 (1·265 to 2·740)	1·911 (1·265 to 2·740)
Lip and oral cavity cancer	Male	8·162 (5·617 to 11·378)	8·162 (5·617 to 11·378)	8·162 (5·617 to 11·378)	8·162 (5·617 to 11·378)	8·162 (5·617 to 11·378)	8·162 (5·617 to 11·378)	8·162 (5·617 to 11·378)
Lip and oral cavity cancer	Female	6·056 (4·232 to 8·541)	6·056 (4·232 to 8·541)	6·056 (4·232 to 8·541)	6·056 (4·232 to 8·541)	6·056 (4·232 to 8·541)	6·056 (4·232 to 8·541)	6·056 (4·232 to 8·541)
Nasopharynx cancer	Male	8·227 (5·677 to 11·505)	8·227 (5·677 to 11·505)	8·227 (5·677 to 11·505)	8·227 (5·677 to 11·505)	8·227 (5·677 to 11·505)	8·227 (5·677 to 11·505)	8·227 (5·677 to 11·505)
Nasopharynx cancer	Female	6·089 (4·288 to 8·470)	6·089 (4·288 to 8·470)	6·089 (4·288 to 8·470)	6·089 (4·288 to 8·470)	6·089 (4·288 to 8·470)	6·089 (4·288 to 8·470)	6·089 (4·288 to 8·470)
Oesophageal cancer	Male	6·676 (4·136 to 10·250)	6·676 (4·136 to 10·250)	6·676 (4·136 to 10·250)	6·676 (4·136 to 10·250)	6·676 (4·136 to 10·250)	6·676 (4·136 to 10·250)	6·676 (4·136 to 10·250)
Oesophageal cancer	Female	6·357 (4·442 to 8·634)	6·357 (4·442 to 8·634)	6·357 (4·442 to 8·634)	6·357 (4·442 to 8·634)	6·357 (4·442 to 8·634)	6·357 (4·442 to 8·634)	6·357 (4·442 to 8·634)
Stomach cancer	Male	1·927 (1·443 to 2·535)	1·927 (1·443 to 2·535)	1·927 (1·443 to 2·535)	1·927 (1·443 to 2·535)	1·927 (1·443 to 2·535)	1·927 (1·443 to 2·535)	1·927 (1·443 to 2·535)
Stomach cancer	Female	1·570 (1·246 to 1·925)	1·570 (1·246 to 1·925)	1·570 (1·246 to 1·925)	1·570 (1·246 to 1·925)	1·570 (1·246 to 1·925)	1·570 (1·246 to 1·925)	1·570 (1·246 to 1·925)
Colon and rectum cancer	Male	1·325 (1·195 to 1·471)	1·325 (1·195 to 1·471)	1·325 (1·195 to 1·471)	1·325 (1·195 to 1·471)	1·325 (1·195 to 1·471)	1·325 (1·195 to 1·471)	1·325 (1·195 to 1·471)
Colon and rectum cancer	Female	1·418 (1·278 to 1·571)	1·418 (1·278 to 1·571)	1·418 (1·278 to 1·571)	1·418 (1·278 to 1·571)	1·418 (1·278 to 1·571)	1·418 (1·278 to 1·571)	1·418 (1·278 to 1·571)
Pancreatic cancer	Male	2·506 (1·962 to 3·111)	2·506 (1·962 to 3·111)	2·506 (1·962 to 3·111)	2·506 (1·962 to 3·111)	2·506 (1·962 to 3·111)	2·506 (1·962 to 3·111)	2·506 (1·962 to 3·111)
Pancreatic cancer	Female	2·098 (1·838 to 2·371)	2·098 (1·838 to 2·371)	2·098 (1·838 to 2·371)	2·098 (1·838 to 2·371)	2·098 (1·838 to 2·371)	2·098 (1·838 to 2·371)	2·098 (1·838 to 2·371)
Larynx cancer	Male	14·602 (8·528 to 23·334)	14·602 (8·528 to 23·334)	14·602 (8·528 to 23·334)	14·602 (8·528 to 23·334)	14·602 (8·528 to 23·334)	14·602 (8·528 to 23·334)	14·602 (8·528 to 23·334)
Larynx cancer	Female	135·959 (23·287 to 465·991)	135·959 (23·287 to 465·991)	135·959 (23·287 to 465·991)	135·959 (23·287 to 465·991)	135·959 (23·287 to 465·991)	135·959 (23·287 to 465·991)	135·959 (23·287 to 465·991)
Tracheal, bronchus, and lung cancer	Male	22·511 (19·062 to 26·715)	22·511 (19·062 to 26·715)	22·511 (19·062 to 26·715)	22·511 (19·062 to 26·715)	22·511 (19·062 to 26·715)	22·511 (19·062 to 26·715)	22·511 (19·062 to 26·715)
Tracheal, bronchus, and lung cancer	Female	14·095 (13·045 to 15·359)	14·095 (13·045 to 15·359)	14·095 (13·045 to 15·359)	14·095 (13·045 to 15·359)	14·095 (13·045 to 15·359)	14·095 (13·045 to 15·359)	14·095 (13·045 to 15·359)
Cervical cancer	Female	1·679 (1·207 to 2·240)	1·679 (1·207 to 2·240)	1·679 (1·207 to 2·240)	1·679 (1·207 to 2·240)	1·679 (1·207 to 2·240)	1·679 (1·207 to 2·240)	1·679 (1·207 to 2·240)
Kidney cancer	Male	2·293 (1·677 to 3·039)	2·293 (1·677 to 3·039)	2·293 (1·677 to 3·039)	2·293 (1·677 to 3·039)	2·293 (1·677 to 3·039)	2·293 (1·677 to 3·039)	2·293 (1·677 to 3·039)

	Sex	65-69 years	70-74 years	75-79 years	80+ years
Diabetes mellitus	Female	1·102 (0·953 to 1·275)	1·102 (0·953 to 1·275)	1·102 (0·953 to 1·275)	1·102 (0·953 to 1·275)
Rheumatoid arthritis	Both	1·375 (1·142 to 1·652)	1·375 (1·142 to 1·652)	1·375 (1·142 to 1·652)	1·375 (1·142 to 1·652)
Cataract	Both	1·671 (1·479 to 1·875)	1·671 (1·479 to 1·875)	1·671 (1·479 to 1·875)	1·671 (1·479 to 1·875)
Macular degeneration	Both	1·911 (1·265 to 2·740)	1·911 (1·265 to 2·740)	1·911 (1·265 to 2·740)	1·911 (1·265 to 2·740)
Lip and oral cavity cancer	Male	8·162 (5·617 to 11·378)	8·162 (5·617 to 11·378)	8·162 (5·617 to 11·378)	8·162 (5·617 to 11·378)
Lip and oral cavity cancer	Female	6·056 (4·232 to 8·541)	6·056 (4·232 to 8·541)	6·056 (4·232 to 8·541)	6·056 (4·232 to 8·541)
Nasopharynx cancer	Male	8·227 (5·677 to 11·505)	8·227 (5·677 to 11·505)	8·227 (5·677 to 11·505)	8·227 (5·677 to 11·505)
Nasopharynx cancer	Female	6·089 (4·288 to 8·470)	6·089 (4·288 to 8·470)	6·089 (4·288 to 8·470)	6·089 (4·288 to 8·470)
Oesophageal cancer	Male	6·676 (4·136 to 10·250)	6·676 (4·136 to 10·250)	6·676 (4·136 to 10·250)	6·676 (4·136 to 10·250)
Oesophageal cancer	Female	6·357 (4·442 to 8·634)	6·357 (4·442 to 8·634)	6·357 (4·442 to 8·634)	6·357 (4·442 to 8·634)
Stomach cancer	Male	1·927 (1·443 to 2·535)	1·927 (1·443 to 2·535)	1·927 (1·443 to 2·535)	1·927 (1·443 to 2·535)
Stomach cancer	Female	1·570 (1·246 to 1·925)	1·570 (1·246 to 1·925)	1·570 (1·246 to 1·925)	1·570 (1·246 to 1·925)
Colon and rectum cancer	Male	1·325 (1·195 to 1·471)	1·325 (1·195 to 1·471)	1·325 (1·195 to 1·471)	1·325 (1·195 to 1·471)
Colon and rectum cancer	Female	1·418 (1·278 to 1·571)	1·418 (1·278 to 1·571)	1·418 (1·278 to 1·571)	1·418 (1·278 to 1·571)
Pancreatic cancer	Male	2·506 (1·962 to 3·111)	2·506 (1·962 to 3·111)	2·506 (1·962 to 3·111)	2·506 (1·962 to 3·111)
Pancreatic cancer	Female	2·098 (1·838 to 2·371)	2·098 (1·838 to 2·371)	2·098 (1·838 to 2·371)	2·098 (1·838 to 2·371)
Larynx cancer	Male	14·602 (8·528 to 23·334)	14·602 (8·528 to 23·334)	14·602 (8·528 to 23·334)	14·602 (8·528 to 23·334)
Larynx cancer	Female	135·959 (23·287 to 465·991)	135·959 (23·287 to 465·991)	135·959 (23·287 to 465·991)	135·959 (23·287 to 465·991)
Tracheal, bronchus, and lung cancer	Male	22·511 (19·062 to 26·715)	22·511 (19·062 to 26·715)	22·511 (19·062 to 26·715)	22·511 (19·062 to 26·715)
Tracheal, bronchus, and lung cancer	Female	14·095 (13·045 to 15·359)	14·095 (13·045 to 15·359)	14·095 (13·045 to 15·359)	14·095 (13·045 to 15·359)
Cervical cancer	Female	1·679 (1·207 to 2·240)	1·679 (1·207 to 2·240)	1·679 (1·207 to 2·240)	1·679 (1·207 to 2·240)
Kidney cancer	Male	2·293 (1·677 to 3·039)	2·293 (1·677 to 3·039)	2·293 (1·677 to 3·039)	2·293 (1·677 to 3·039)

	Sex	30-34 years	35-39 years	40-44 years	45-49 years	50-54 years	55-59 years	60-64 years
Kidney cancer	Female	1·518 (1·204 to 1·874)	1·518 (1·204 to 1·874)	1·518 (1·204 to 1·874)	1·518 (1·204 to 1·874)	1·518 (1·204 to 1·874)	1·518 (1·204 to 1·874)	1·518 (1·204 to 1·874)
Bladder cancer	Male	3·332 (2·364 to 4·558)	3·332 (2·364 to 4·558)	3·332 (2·364 to 4·558)	3·332 (2·364 to 4·558)	3·332 (2·364 to 4·558)	3·332 (2·364 to 4·558)	3·332 (2·364 to 4·558)
Bladder cancer	Female	2·582 (1·923 to 3·420)	2·582 (1·923 to 3·420)	2·582 (1·923 to 3·420)	2·582 (1·923 to 3·420)	2·582 (1·923 to 3·420)	2·582 (1·923 to 3·420)	2·582 (1·923 to 3·420)
Leukaemia	Male	2·013 (1·390 to 2·873)	2·013 (1·390 to 2·873)	2·013 (1·390 to 2·873)	2·013 (1·390 to 2·873)	2·013 (1·390 to 2·873)	2·013 (1·390 to 2·873)	2·013 (1·390 to 2·873)
Leukaemia	Female	1·163 (0·894 to 1·479)	1·163 (0·894 to 1·479)	1·163 (0·894 to 1·479)	1·163 (0·894 to 1·479)	1·163 (0·894 to 1·479)	1·163 (0·894 to 1·479)	1·163 (0·894 to 1·479)
Chronic obstructive pulmonary disease	Male	11·546 (8·894 to 14·932)	11·546 (8·894 to 14·932)	11·546 (8·894 to 14·932)	11·546 (8·894 to 14·932)	11·546 (8·894 to 14·932)	11·546 (8·894 to 14·932)	11·546 (8·894 to 14·932)
Chronic obstructive pulmonary disease	Female	15·257 (13·637 to 17·152)	15·257 (13·637 to 17·152)	15·257 (13·637 to 17·152)	15·257 (13·637 to 17·152)	15·257 (13·637 to 17·152)	15·257 (13·637 to 17·152)	15·257 (13·637 to 17·152)
Interstitial lung disease and pulmonary sarcoidosis	Male	2·086 (1·774 to 2·441)	2·086 (1·774 to 2·441)	2·086 (1·774 to 2·441)	2·086 (1·774 to 2·441)	2·086 (1·774 to 2·441)	2·086 (1·774 to 2·441)	2·086 (1·774 to 2·441)
Interstitial lung disease and pulmonary sarcoidosis	Female	1·967 (1·768 to 2·176)	1·967 (1·768 to 2·176)	1·967 (1·768 to 2·176)	1·967 (1·768 to 2·176)	1·967 (1·768 to 2·176)	1·967 (1·768 to 2·176)	1·967 (1·768 to 2·176)
Other chronic respiratory diseases	Male	2·100 (1·774 to 2·462)	2·100 (1·774 to 2·462)	2·100 (1·774 to 2·462)	2·100 (1·774 to 2·462)	2·100 (1·774 to 2·462)	2·100 (1·774 to 2·462)	2·100 (1·774 to 2·462)
Other chronic respiratory diseases	Female	1·982 (1·800 to 2·172)	1·982 (1·800 to 2·172)	1·982 (1·800 to 2·172)	1·982 (1·800 to 2·172)	1·982 (1·800 to 2·172)	1·982 (1·800 to 2·172)	1·982 (1·800 to 2·172)
Hip Fracture	Both	1·850 (1·518 to 2·250)	1·850 (1·518 to 2·250)	1·850 (1·518 to 2·250)	1·850 (1·518 to 2·250)	1·850 (1·518 to 2·250)	1·850 (1·518 to 2·250)	1·850 (1·518 to 2·250)
Non-Hip Fracture	Both	1·250 (1·153 to 1·359)	1·250 (1·153 to 1·359)	1·250 (1·153 to 1·359)	1·250 (1·153 to 1·359)	1·250 (1·153 to 1·359)	1·250 (1·153 to 1·359)	1·250 (1·153 to 1·359)

	Sex	65-69 years	70-74 years	75-79 years	80+ years
Kidney cancer	Female	1·518 (1·204 to 1·874)	1·518 (1·204 to 1·874)	1·518 (1·204 to 1·874)	1·518 (1·204 to 1·874)
Bladder cancer	Male	3·332 (2·364 to 4·558)	3·332 (2·364 to 4·558)	3·332 (2·364 to 4·558)	3·332 (2·364 to 4·558)
Bladder cancer	Female	2·582 (1·923 to 3·420)	2·582 (1·923 to 3·420)	2·582 (1·923 to 3·420)	2·582 (1·923 to 3·420)
Leukaemia	Male	2·013 (1·390 to 2·873)	2·013 (1·390 to 2·873)	2·013 (1·390 to 2·873)	2·013 (1·390 to 2·873)
Leukaemia	Female	1·163 (0·894 to 1·479)	1·163 (0·894 to 1·479)	1·163 (0·894 to 1·479)	1·163 (0·894 to 1·479)
Chronic obstructive pulmonary disease	Male	11·546 (8·894 to 14·932)	11·546 (8·894 to 14·932)	11·546 (8·894 to 14·932)	11·546 (8·894 to 14·932)
Chronic obstructive pulmonary disease	Female	15·257 (13·637 to 17·152)	15·257 (13·637 to 17·152)	15·257 (13·637 to 17·152)	15·257 (13·637 to 17·152)
Interstitial lung disease and pulmonary sarcoidosis	Male	2·086 (1·774 to 2·441)	2·086 (1·774 to 2·441)	2·086 (1·774 to 2·441)	2·086 (1·774 to 2·441)
Interstitial lung disease and pulmonary sarcoidosis	Female	1·967 (1·768 to 2·176)	1·967 (1·768 to 2·176)	1·967 (1·768 to 2·176)	1·967 (1·768 to 2·176)
Other chronic respiratory diseases	Male	2·100 (1·774 to 2·462)	2·100 (1·774 to 2·462)	2·100 (1·774 to 2·462)	2·100 (1·774 to 2·462)
Other chronic respiratory diseases	Female	1·982 (1·800 to 2·172)	1·982 (1·800 to 2·172)	1·982 (1·800 to 2·172)	1·982 (1·800 to 2·172)
Hip Fracture	Both	1·850 (1·518 to 2·250)	1·850 (1·518 to 2·250)	1·850 (1·518 to 2·250)	1·850 (1·518 to 2·250)
Non-Hip Fracture	Both	1·250 (1·153 to 1·359)	1·250 (1·153 to 1·359)	1·250 (1·153 to 1·359)	1·250 (1·153 to 1·359)

Table S8

Country or Territory	Male Smoking Attributable DALYs 2015 (95% UI)	Male Smoking Attributable Deaths 2015 (95% UI)	Male Smoking Attributable DALY Rate 2015, per 100 (95% UI)	Male Smoking Attributable Death Rate 2015, per 100 (95% UI)	Female Smoking Attributable DALYs 2015 (95% UI)	Female Smoking Attributable Deaths 2015 (95% UI)	Female Smoking Attributable DALY Rate 2015, per 100 (95% UI)	Female Smoking Attributable Death Rate 2015, per 100 (95% UI)
Global	120092504.0 (107836744.0 - 132522080.0)	4959156.0 (4451601.0 - 5465780.5)	36.7 (33 - 40.5)	1.7 (1.5 - 1.9)	28530670 (24894928 - 32916060)	1443038.4 (1239590.9 - 1664258.4)	8 (7 - 9.3)	0.4 (0.4 - 0.5)
Afghanistan	487982.9 (313737.8 - 694353.3)	15301.9 (9700.5 - 21600.1)	68.5 (44.1 - 95.2)	2.6 (1.7 - 3.6)	337174.4 (188783.6 - 534311.1)	10163.6 (5703.2 - 15892.3)	45.1 (25.4 - 70)	1.6 (0.9 - 2.4)
Albania	65824.5 (55668.7 - 76563.3)	2993.6 (2466.4 - 3555.8)	38.3 (32.6 - 44.4)	1.8 (1.5 - 2.1)	9040.6 (7379.6 - 10844)	469.4 (364.2 - 598.2)	4.9 (4 - 5.9)	0.2 (0.2 - 0.3)
Algeria	240717.8 (188012 - 299717)	8455.7 (6424.4 - 10633.1)	15.8 (12.4 - 19.6)	0.6 (0.5 - 0.8)	34093.8 (21164.5 - 50070.7)	1294.7 (786.3 - 2026.9)	2.2 (1.4 - 3.3)	0.1 (0.1 - 0.2)
American Samoa	1085.1 (799.6 - 1409.3)	31.2 (23.1 - 40.3)	44.7 (34 - 56.2)	1.8 (1.4 - 2.2)	494.8 (353.9 - 673)	15.1 (10.8 - 20.9)	17.9 (13.1 - 23.8)	0.7 (0.5 - 1)
Andorra	1125.4 (969.2 - 1278.9)	67 (57.7 - 76.7)	18.2 (15.6 - 20.8)	1 (0.8 - 1.1)	269 (190.9 - 343.5)	19.5 (12.9 - 26.7)	3.7 (2.6 - 4.7)	0.2 (0.1 - 0.2)
Angola	177659.7 (57776 - 596549.1)	5577.6 (1770.1 - 18235.6)	36.3 (12.4 - 114.8)	1.4 (0.5 - 4.2)	68215.8 (5623.2 - 270962.5)	2302.8 (184.5 - 8410.9)	13.2 (1.1 - 47.6)	0.5 (0 - 1.8)
Antigua and Barbuda	337 (243.5 - 424.6)	12.7 (8.7 - 16.1)	8.4 (6 - 10.6)	0.4 (0.2 - 0.5)	89.9 (57.9 - 124.8)	3.8 (2.4 - 5.5)	1.9 (1.2 - 2.6)	0.1 (0.1 - 0.1)
Argentina	678571.6 (624431.9 - 738296.4)	30754 (28263.4 - 33659.5)	34 (31.3 - 37.1)	1.6 (1.5 - 1.8)	283905.3 (255063.9 - 314399.2)	14722.9 (12599.6 - 16974.8)	11.5 (10.3 - 12.7)	0.5 (0.5 - 0.6)
Armenia	102410.5 (91469.7 - 113149.8)	4314.9 (3817.2 - 4809.1)	66.8 (59.7 - 73.9)	2.9 (2.6 - 3.3)	10444.2 (8281.5 - 12528.6)	472.3 (323.5 - 618.1)	5.1 (4 - 6)	0.2 (0.2 - 0.3)
Australia	242428.1 (223159.9 - 262672.4)	13697.3 (12595.3 - 14865.7)	15.7 (14.4 - 17)	0.9 (0.8 - 0.9)	140109.2 (128896 - 151736.5)	8544.6 (7732.6 - 9421)	8.3 (7.6 - 9)	0.4 (0.4 - 0.5)
Austria	141686.6 (130047.4 - 152745.6)	6724.5 (6152.4 - 7268.7)	22.2 (20.3 - 24)	1 (0.9 - 1.1)	64126.5 (58815.6 - 70175.6)	3523.7 (3062.1 - 4027.1)	8.7 (8 - 9.5)	0.4 (0.3 - 0.4)
Azerbaijan	240046.2 (205857.1 - 276125.2)	8132.5 (6877.4 - 9511.8)	54.9 (46.6 - 63.7)	2.2 (1.8 - 2.6)	17088.6 (11893.4 - 22370)	542.6 (377 - 728.2)	3.4 (2.4 - 4.4)	0.1 (0.1 - 0.2)
Bahrain	5978.6 (4101 - 8313.5)	169.6 (115.7 - 234.5)	12.4 (8.7 - 16.7)	0.6 (0.4 - 0.8)	1844.2 (1222.1 - 2634)	56.9 (36.2 - 82.3)	5.7 (3.7 - 8.1)	0.3 (0.2 - 0.4)
Bangladesh	3281736.8 (2657121 - 4073151.3)	132537.2 (109776.3 - 161398.8)	61.3 (50.3 - 75.2)	2.9 (2.4 - 3.5)	521260.5 (340818.2 - 772432.9)	20108.2 (12601.8 - 30675.9)	10.2 (6.5 - 15.4)	0.4 (0.3 - 0.7)
Barbados	1762.8 (1398.8 - 2172.2)	78.4 (59.2 - 103)	10.3 (8.2 - 12.8)	0.5 (0.3 - 0.6)	413.6 (272.9 - 577.5)	18.5 (11 - 28.2)	2 (1.4 - 2.8)	0.1 (0.1 - 0.1)
Belarus	517728.8 (462445.6 - 573529.1)	20478.3 (18286.4 - 22855.9)	93 (83.3 - 103)	3.9 (3.5 - 4.3)	48851.8 (39116.2 - 59507)	1788.8 (1385.2 - 2299.5)	6.6 (5.4 - 7.9)	0.2 (0.2 - 0.3)
Belgium	255211 (234769.7 - 277281.3)	13875.4 (12655 - 15105.9)	30.8 (28.3 - 33.5)	1.6 (1.4 - 1.7)	93450.4 (83232.5 - 103689.6)	5312.1 (4485.7 - 6147.5)	10.1 (9.1 - 11.2)	0.5 (0.4 - 0.5)
Belize	2855.7 (2259.8 - 3468.2)	103.1 (83.1 - 125.2)	27.1 (21.8 - 33.2)	1.2 (1 - 1.4)	648.3 (479.3 - 823.1)	23.5 (17.5 - 30)	6.1 (4.6 - 7.7)	0.3 (0.2 - 0.4)
Benin	69981.4 (22107.5 - 169330.8)	2209.6 (683.3 - 5189.9)	27.7 (9.3 - 63.1)	1.1 (0.4 - 2.3)	13920.6 (2424.8 - 44668.4)	545 (86 - 1689.9)	5.7 (1 - 17.3)	0.3 (0 - 0.8)
Bermuda	806.6 (683 - 941.6)	35.1 (30.3 - 40.4)	25.5 (21.6 - 29.7)	1.2 (1 - 1.4)	185 (135 - 238.1)	11 (8.1 - 14.2)	5.3 (3.9 - 6.8)	0.3 (0.2 - 0.4)
Bhutan	3187.5 (1803.7 - 5141.1)	114.9 (55.1 - 197.9)	10.4 (5.5 - 17.2)	0.5 (0.2 - 0.8)	860.7 (436.2 - 1820)	27.9 (13.4 - 62.5)	3.5 (1.7 - 7.7)	0.1 (0.1 - 0.3)
Bolivia	80367.9 (59954.3 - 109563.7)	3486.4 (2583.9 - 4673.6)	21.8 (16.3 - 29.8)	1 (0.8 - 1.4)	35342 (23350.8 - 48909.3)	1738.4 (1086 - 2466.7)	9 (5.9 - 12.5)	0.4 (0.3 - 0.6)
Bosnia and Herzegovina	105945.9 (97204.8 - 115295.1)	4435.8 (4038.1 - 4832.3)	39.4 (36.2 - 42.8)	1.7 (1.6 - 1.9)	29335.9 (25011 - 33897.1)	1338.7 (1068.4 - 1630.8)	9.9 (8.5 - 11.4)	0.4 (0.3 - 0.5)
Botswana	41420.6 (11998.2 - 136380)	1318 (374.6 - 4201.5)	63.4 (19.6 - 195.4)	2.5 (0.8 - 7.1)	13112 (2541.4 - 61345.9)	471.2 (90.9 - 2085.8)	19.6 (4.1 - 85.5)	0.8 (0.2 - 3.3)
Brazil	2209194 (1979124.1 - 2462033.8)	94203.9 (84767.9 - 104092.2)	25.4 (22.8 - 28.2)	1.3 (1.1 - 1.4)	1223426.3 (1095315.1 - 1344622.6)	55069.5 (49694.4 - 61074.3)	11.6 (10.4 - 12.8)	0.6 (0.5 - 0.6)

Country or Territory	Male Smoking Attributable DALYs 2015 (95% UI)	Male Smoking Attributable Deaths 2015 (95% UI)	Male Smoking Attributable DALY Rate 2015, per 100 (95% UI)	Male Smoking Attributable Death Rate 2015, per 100 (95% UI)	Female Smoking Attributable DALYs 2015 (95% UI)	Female Smoking Attributable Deaths 2015 (95% UI)	Female Smoking Attributable DALY Rate 2015, per 100 (95% UI)	Female Smoking Attributable Death Rate 2015, per 100 (95% UI)
Brunei	3647.9 (3065.2 - 4241.5)	135.7 (115.9 - 155)	24.2 (20.6 - 27.7)	1.2 (1.1 - 1.4)	1370 (1133.3 - 1626.9)	58.3 (49.5 - 67.9)	9.4 (8 - 10.9)	0.5 (0.4 - 0.6)
Bulgaria	312077.5 (283998.7 - 340487.4)	12537.5 (11305.1 - 13753.4)	58.2 (53 - 63.4)	2.3 (2 - 2.5)	83973 (73096 - 95901.1)	3385.8 (2810 - 4138.7)	13.9 (12.2 - 15.7)	0.5 (0.4 - 0.6)
Burkina Faso	65753.4 (28576.6 - 149576.3)	1826.5 (735.2 - 4352.4)	17.4 (7.4 - 40.1)	0.6 (0.3 - 1.4)	31004.9 (11255 - 79484.1)	1023.5 (348.7 - 2651)	7.6 (2.8 - 18.8)	0.3 (0.1 - 0.7)
Burundi	46498.7 (18443.9 - 98777.2)	1433.6 (543.1 - 3067.7)	18.6 (7.5 - 38.9)	0.7 (0.3 - 1.4)	6576.4 (2500.7 - 18176.3)	225.5 (85.6 - 618.6)	2.7 (1.1 - 7.4)	0.1 (0 - 0.3)
Cambodia	266780.3 (218725.8 - 328882.8)	9903.8 (8107.5 - 12088.1)	67.9 (56.3 - 82.1)	3.2 (2.7 - 3.9)	65672.2 (44356 - 90317.7)	2509.7 (1659.7 - 3473)	12.7 (8.5 - 17.2)	0.6 (0.4 - 0.8)
Cameroon	138878.4 (50723.7 - 330925.4)	4587.7 (1683.8 - 10775.9)	26.2 (9.9 - 60.8)	1 (0.4 - 2.3)	33118.3 (6607.4 - 99635.2)	1320.8 (244 - 3838.8)	6.5 (1.3 - 18.9)	0.3 (0.1 - 0.8)
Canada	485976.2 (446893 - 529046.3)	26318.6 (24131.9 - 28762.8)	19.3 (17.8 - 20.9)	1 (1 - 1.1)	331334 (305981.3 - 356321.3)	19338.4 (17528.8 - 21284.9)	11.9 (11 - 12.8)	0.6 (0.6 - 0.7)
Cape Verde	2922.8 (1989.6 - 4218.3)	117.7 (80.6 - 169.5)	21.7 (15 - 30.5)	1 (0.7 - 1.4)	977.5 (679.7 - 1543.6)	44.1 (28.9 - 70.8)	5.7 (4 - 9)	0.3 (0.2 - 0.4)
Central African Republic	69972.8 (14478.7 - 149232.9)	2254.5 (446.6 - 4656.9)	55 (11.7 - 114.7)	2 (0.4 - 4)	16409.4 (1441.4 - 49385.1)	601.8 (50.6 - 1753)	12.3 (1.1 - 36.2)	0.5 (0 - 1.4)
Chad	63216.5 (22183.5 - 157364.6)	2082 (717.3 - 5103.9)	24.1 (8.9 - 58)	1 (0.4 - 2.3)	15864.6 (3199.9 - 51759.5)	610.6 (113.2 - 1969.5)	6.1 (1.3 - 19.3)	0.3 (0.1 - 0.9)
Chile	157874.3 (136470.6 - 177660.7)	7730.7 (6663.9 - 8837.7)	17.2 (14.9 - 19.4)	0.9 (0.8 - 1)	96234.7 (83977.9 - 109756.1)	5622.4 (4789.9 - 6558.7)	8.5 (7.4 - 9.8)	0.5 (0.4 - 0.5)
China	32149490.0 (24106296.0 - 40881364.0)	1448653.4 (1069346.4 - 1825899.1)	42.1 (31.5 - 53.2)	2.2 (1.6 - 2.8)	4761507 (2007050.4 - 8643378)	318708.3 (131386.6 - 533137)	6.6 (2.8 - 11.7)	0.5 (0.2 - 0.8)
Colombia	251820.9 (217019.3 - 289722.4)	11616.8 (10024.1 - 13279.1)	13.7 (11.9 - 15.6)	0.8 (0.7 - 0.9)	131596.5 (112302.4 - 154029.2)	7634.6 (6421.8 - 8983.4)	6.2 (5.3 - 7.2)	0.4 (0.3 - 0.5)
Comoros	3480.8 (1543.3 - 7110)	102 (43 - 217.1)	17.1 (7.6 - 34.7)	0.6 (0.3 - 1.3)	393.1 (158.3 - 1006.1)	12.2 (4.7 - 30.8)	1.9 (0.8 - 4.7)	0.1 (0 - 0.2)
Congo	25432.6 (12304.7 - 57030.7)	895.6 (427.6 - 1989.7)	22.7 (11.1 - 50.1)	1 (0.5 - 2.1)	16001.3 (3120.2 - 39200.8)	582.4 (102.6 - 1369.7)	13.6 (2.7 - 32)	0.5 (0.1 - 1.3)
Costa Rica	18570.7 (15215.4 - 21950.4)	925.9 (761.6 - 1097.3)	8.4 (6.9 - 9.9)	0.5 (0.4 - 0.6)	5755.9 (3850.7 - 7433)	361.3 (186.5 - 508.1)	2.4 (1.6 - 3.1)	0.2 (0.1 - 0.2)
Cote d'Ivoire	169926.8 (63874.8 - 363865.1)	5113 (1787.9 - 11048.4)	26.4 (9.9 - 55.6)	0.9 (0.3 - 1.9)	25381.4 (7081.1 - 63527.9)	1099.4 (309.5 - 2688.5)	6.3 (1.9 - 15.1)	0.3 (0.1 - 0.8)
Croatia	125684 (115378.8 - 136444.7)	5710.5 (5242.9 - 6245.7)	40.9 (37.5 - 44.4)	1.8 (1.7 - 2)	44176.6 (38753.1 - 50674)	2309.3 (1910.1 - 2773.3)	11.7 (10.4 - 13.2)	0.5 (0.4 - 0.6)
Cuba	252682.4 (227339.9 - 278654.2)	12584.3 (11243 - 14158.1)	33.2 (29.8 - 36.5)	1.7 (1.5 - 1.9)	119395.3 (104816.7 - 136218.1)	6733.3 (5668.7 - 8131.9)	14.5 (12.8 - 16.6)	0.8 (0.6 - 0.9)
Cyprus	13735.2 (12379.5 - 15181.6)	636.2 (572.1 - 705.7)	25.2 (22.7 - 27.9)	1.1 (1 - 1.3)	2251.6 (1875.9 - 2641.7)	80.7 (63.1 - 100.7)	3.8 (3.2 - 4.5)	0.1 (0.1 - 0.2)
Czech Republic	268931.3 (248106.3 - 290949.5)	12023.2 (11034.2 - 13079.3)	34.7 (31.9 - 37.6)	1.6 (1.5 - 1.7)	101668.2 (90544.8 - 114801.4)	5663.7 (4740.7 - 6774.3)	10.9 (9.7 - 12.2)	0.5 (0.5 - 0.6)
Democratic Republic of the Congo	455471.3 (195609.1 - 855370.4)	14633.1 (6109.9 - 27910.9)	27 (11.9 - 50.6)	1 (0.4 - 2)	79224.9 (14392.2 - 211771.8)	2943.9 (521 - 8248.1)	4.8 (0.9 - 12.8)	0.2 (0 - 0.6)
Denmark	103308.4 (95273.8 - 113134)	5688 (5227.6 - 6239.5)	23.8 (21.9 - 26)	1.3 (1.2 - 1.4)	84471.5 (78336.8 - 91174.8)	5255.4 (4837.3 - 5730.9)	17.4 (16 - 18.7)	1 (0.9 - 1)
Djibouti	11838.8 (4399.2 - 30455.8)	386.5 (140.3 - 960)	43.1 (16.6 - 105)	1.7 (0.7 - 3.9)	1319.5 (474.6 - 4450.2)	45.6 (15.8 - 154)	4.8 (1.8 - 15.7)	0.2 (0.1 - 0.6)
Dominica	568.1 (448.7 - 701.2)	22.1 (17 - 28.1)	16.8 (13.2 - 20.8)	0.7 (0.5 - 0.9)	96.1 (65.1 - 131.3)	4.5 (2.8 - 6.4)	2.6 (1.8 - 3.6)	0.1 (0.1 - 0.2)
Dominican Republic	54252.3 (44522.5 - 65243.4)	2439.3 (1985.4 - 2936.1)	13.8 (11.4 - 16.6)	0.7 (0.5 - 0.8)	33137 (27530.2 - 39553.3)	1640.9 (1309.7 - 2085.2)	7.9 (6.5 - 9.4)	0.4 (0.3 - 0.5)

Country or Territory	Male Smoking Attributable DALYs 2015 (95% UI)	Male Smoking Attributable Deaths 2015 (95% UI)	Male Smoking Attributable DALY Rate 2015, per 100 (95% UI)	Male Smoking Attributable Death Rate 2015, per 100 (95% UI)	Female Smoking Attributable DALYs 2015 (95% UI)	Female Smoking Attributable Deaths 2015 (95% UI)	Female Smoking Attributable DALY Rate 2015, per 100 (95% UI)	Female Smoking Attributable Death Rate 2015, per 100 (95% UI)
Ecuador	69002.4 (54896.2 - 85304.5)	3604.8 (2848 - 4473.5)	12.1 (9.7 - 14.9)	0.7 (0.6 - 0.9)	21428.7 (15125.9 - 28248.7)	1292.1 (888.8 - 1709.5)	3.3 (2.4 - 4.4)	0.2 (0.1 - 0.3)
Egypt	1342571.8 (1172280.3 - 1524147.1)	45274.1 (39542.3 - 51391.3)	43.5 (37.8 - 49.4)	1.8 (1.5 - 2)	79576.4 (57025.2 - 103011.3)	2483.1 (1787.2 - 3353.8)	2.3 (1.7 - 3)	0.1 (0.1 - 0.1)
El Salvador	20515.9 (15628.1 - 26550.2)	780.9 (569.2 - 1031.4)	9 (6.8 - 11.7)	0.4 (0.3 - 0.5)	10568.5 (7282.8 - 14141.1)	490.1 (304.5 - 694.9)	3.6 (2.5 - 4.8)	0.2 (0.1 - 0.2)
Equatorial Guinea	6049 (2277.3 - 21740.7)	198.8 (72.5 - 704.8)	23.3 (9.4 - 76.8)	1 (0.4 - 3)	2011.8 (171.3 - 8940.7)	68.5 (5.2 - 296.8)	9.1 (0.8 - 38.1)	0.4 (0 - 1.6)
Eritrea	28548.5 (9370 - 70584)	804.4 (241.9 - 1994.2)	23.6 (7.7 - 56.9)	0.8 (0.3 - 2)	4106.2 (719 - 16242)	139.7 (25.9 - 527.1)	3.4 (0.6 - 13.1)	0.1 (0 - 0.5)
Estonia	31575.4 (28441.9 - 34902)	1420.6 (1274 - 1571.4)	38.9 (35 - 43)	1.7 (1.6 - 1.9)	8176 (6875.2 - 9609.4)	398.6 (317.6 - 500.8)	6.6 (5.7 - 7.7)	0.3 (0.2 - 0.3)
Ethiopia	338194.7 (142757.7 - 782924.3)	11393.3 (4505.4 - 26188.2)	14.8 (6.2 - 33.7)	0.6 (0.2 - 1.3)	39917.9 (15487.3 - 111124.9)	1670.3 (674 - 4405.3)	1.8 (0.7 - 4.8)	0.1 (0 - 0.2)
Federated States of Micronesia	1753.6 (1084.5 - 2899)	58.9 (37.7 - 97.1)	56.5 (36.1 - 92.1)	2.2 (1.4 - 3.5)	517.6 (267.8 - 968.5)	18.4 (9.5 - 34.6)	16.1 (8.4 - 29.8)	0.6 (0.3 - 1.2)
Fiji	18054.9 (12943.8 - 24324.2)	545.9 (389.6 - 734.4)	45.7 (32.9 - 61.2)	1.6 (1.1 - 2.1)	3361.8 (2246.6 - 4741.5)	109.4 (72.9 - 155.2)	8.7 (5.8 - 12.3)	0.3 (0.2 - 0.5)
Finland	78775.6 (70823 - 87945.2)	3895.4 (3504.9 - 4329.4)	18.2 (16.3 - 20.3)	0.9 (0.8 - 1)	29393.5 (26223.8 - 32969.3)	1531.4 (1307.7 - 1787.5)	6 (5.3 - 6.7)	0.3 (0.2 - 0.3)
France	1234992.3 (1153562.5 - 1318153.4)	59120.5 (54489.1 - 63808)	27.3 (25.5 - 29.2)	1.2 (1.1 - 1.3)	313740.4 (276506.3 - 350029.6)	17249.9 (13312.8 - 20977.4)	6.3 (5.6 - 6.9)	0.2 (0.2 - 0.3)
Gabon	14023.2 (6979.3 - 29601.2)	513.1 (254.3 - 1074.1)	27.7 (13.9 - 58)	1.2 (0.6 - 2.4)	4407.8 (1158.6 - 10277.7)	174 (41.7 - 414.2)	8.6 (2.3 - 19.4)	0.4 (0.1 - 0.8)
Georgia	155322.4 (135036.6 - 175764.1)	6225.3 (5349.5 - 7130.3)	69.6 (60.5 - 78.8)	2.8 (2.4 - 3.2)	12945.3 (10085.3 - 16382.3)	525.5 (384.7 - 736)	4.5 (3.6 - 5.6)	0.2 (0.1 - 0.2)
Germany	1706861.6 (1586648.8 - 1830848.9)	85343.9 (79061.6 - 91703.6)	24.3 (22.6 - 26)	1.1 (1 - 1.2)	780908.9 (716628.8 - 842630.2)	44786.9 (39512 - 50140.3)	9.9 (9.1 - 10.7)	0.4 (0.4 - 0.5)
Ghana	119490.2 (47975.1 - 276514.2)	4256.5 (1722.2 - 9507.4)	18.5 (7.9 - 40.6)	0.8 (0.4 - 1.7)	23024 (6176.7 - 73118)	1069.5 (293.5 - 3007.4)	3.6 (1 - 10.4)	0.2 (0.1 - 0.5)
Greece	296225.3 (273479.9 - 319753)	15471.3 (14120.9 - 16796)	35.6 (32.9 - 38.5)	1.6 (1.5 - 1.7)	101016.2 (89585.4 - 113091.9)	6368.4 (5319.9 - 7507.5)	10 (9 - 11.2)	0.5 (0.4 - 0.5)
Greenland	1920.1 (1727.9 - 2145.9)	71.3 (64.7 - 78.9)	65.5 (59.5 - 72.2)	3.2 (2.9 - 3.5)	1096.8 (989.1 - 1242.5)	41.4 (37.8 - 46.8)	49.1 (44.8 - 55.2)	2.4 (2.2 - 2.7)
Grenada	1009.8 (818 - 1204.9)	36.7 (29.9 - 43.7)	24.6 (20.2 - 29.1)	1 (0.8 - 1.2)	260.1 (198.9 - 334.5)	11.3 (8.3 - 15.1)	5.7 (4.4 - 7.3)	0.2 (0.2 - 0.3)
Guam	4850.7 (3795.2 - 6128.7)	182.7 (146.1 - 228.4)	58.3 (46.3 - 73.1)	2.5 (2 - 3)	2029.5 (1530.2 - 2603.7)	79 (58.9 - 99.1)	23.5 (17.7 - 30)	0.9 (0.7 - 1.2)
Guatemala	51557.4 (37966.9 - 66873.6)	1703 (1222 - 2236.6)	11.3 (8.1 - 14.6)	0.4 (0.3 - 0.6)	15729.1 (8856.1 - 24464.4)	727.1 (352.4 - 1171.7)	3.1 (1.7 - 4.8)	0.2 (0.1 - 0.3)
Guinea	84559.2 (29082 - 182918.2)	2440 (796 - 5452.3)	24.8 (8.8 - 54.1)	0.8 (0.3 - 1.8)	18116.2 (4152.7 - 46442.2)	630.2 (137.8 - 1611.2)	5.8 (1.4 - 14.5)	0.2 (0.1 - 0.6)
Guinea-Bissau	14961.4 (4194.1 - 54652.6)	464 (125.4 - 1650.4)	31.1 (9 - 107.9)	1.2 (0.3 - 3.8)	4299.8 (280.8 - 20705.4)	147 (8.5 - 648.6)	8.7 (0.6 - 38.9)	0.3 (0 - 1.4)
Guyana	10499.1 (7720.8 - 13464.4)	340.6 (248.8 - 436.7)	32.5 (24.2 - 41.3)	1.3 (0.9 - 1.6)	1667 (1093.2 - 2379)	53.6 (35.6 - 75.5)	4.8 (3.2 - 6.7)	0.2 (0.1 - 0.2)
Haiti	67123 (41747.2 - 93995.1)	2212.6 (1360.2 - 3151.2)	20.7 (12.9 - 29)	0.8 (0.5 - 1.1)	39735.2 (20971.4 - 63713.3)	1454.2 (748.1 - 2362.5)	11.3 (6 - 17.9)	0.5 (0.2 - 0.7)
Honduras	39563.7 (27247.3 - 57291)	1430.8 (941.4 - 2203.8)	16 (11 - 23.6)	0.7 (0.4 - 1)	12277.4 (4873.1 - 21578.7)	509 (173.3 - 986.5)	5 (1.9 - 8.7)	0.2 (0.1 - 0.4)
Hungary	333900.8 (306315.7 - 361592)	13972.5 (12794.3 - 15184.9)	50.6 (46.5 - 54.8)	2.1 (1.9 - 2.3)	161786.4 (147247.4 - 178705)	7597.7 (6724 - 8636.1)	19 (17.3 - 20.9)	0.8 (0.7 - 0.8)

Country or Territory	Male Smoking Attributable DALYs 2015 (95% UI)	Male Smoking Attributable Deaths 2015 (95% UI)	Male Smoking Attributable DALY Rate 2015, per 100 (95% UI)	Male Smoking Attributable Death Rate 2015, per 100 (95% UI)	Female Smoking Attributable DALYs 2015 (95% UI)	Female Smoking Attributable Deaths 2015 (95% UI)	Female Smoking Attributable DALY Rate 2015, per 100 (95% UI)	Female Smoking Attributable Death Rate 2015, per 100 (95% UI)
Iceland	2877<br>(2571.9 - 3195.3)	147.8<br>(131.5 - 163.6)	14.2<br>(12.7 - 15.8)	0.7<br>(0.6 - 0.8)	2063.5<br>(1794.2 - 2349.6)	118.3<br>(99.2 - 137.7)	9.4<br>(8.2 - 10.8)	0.5<br>(0.4 - 0.5)
India	17630850<br>(15397011 - 20250192)	627974.9<br>(542341.7 - 724523.6)	34.9<br>(30.4 - 39.9)	1.5<br>(1.2 - 1.7)	2922610.3<br>(2044289.9 - 3891200.5)	115509.6<br>(79022.3 - 156903.6)	6.1<br>(4.2 - 8.1)	0.3<br>(0.2 - 0.4)
Indonesia	4869017.5<br>(3597240 - 6275432.5)	156664<br>(112804.2 - 201676.5)	47.3<br>(34.9 - 60.4)	1.9<br>(1.4 - 2.4)	685382.3<br>(451664.3 - 994825.6)	23384.1<br>(15286.9 - 34030.2)	6.4<br>(4.2 - 9.2)	0.3<br>(0.2 - 0.4)
Iran	893756.8<br>(647892.1 - 1213038)	30808.7<br>(22207.3 - 41447.5)	28.7<br>(21 - 38.4)	1.2<br>(0.8 - 1.5)	117948.9<br>(59617.2 - 194806.6)	4234.9<br>(1941.9 - 7130.3)	4<br>(2 - 6.6)	0.2<br>(0.1 - 0.3)
Iraq	440150.9<br>(282377.7 - 638103.4)	14409.1<br>(8923.8 - 20395.7)	52.1<br>(33.5 - 72.5)	2.1<br>(1.3 - 2.8)	96339.5<br>(56742.6 - 150977.4)	3405.6<br>(1980.6 - 5313)	10.5<br>(6.2 - 16.2)	0.4<br>(0.3 - 0.7)
Ireland	58278.2<br>(53222.9 - 63713.5)	3070.9<br>(2785.9 - 3359.3)	21.6<br>(19.8 - 23.6)	1.2<br>(1.1 - 1.3)	32161.6<br>(27751.7 - 37228.2)	2098.3<br>(1749.4 - 2514.3)	10.6<br>(9.2 - 12.4)	0.6<br>(0.5 - 0.7)
Israel	69718<br>(63457.3 - 76580.4)	3341.1<br>(3008.3 - 3687.2)	17.9<br>(16.3 - 19.7)	0.8<br>(0.8 - 0.9)	27416.3<br>(24326.4 - 30921.8)	1675.5<br>(1431.4 - 1964.5)	5.9<br>(5.2 - 6.6)	0.3<br>(0.3 - 0.4)
Italy	1140140.3<br>(1055475.1 - 1224550.6)	68685.4<br>(63029.5 - 74455.4)	22.3<br>(20.6 - 24)	1.2<br>(1.1 - 1.3)	385958.1<br>(341480.8 - 430356.4)	27232.2<br>(22933.7 - 31845)	6.3<br>(5.6 - 6.9)	0.3<br>(0.3 - 0.4)
Jamaica	29083.7<br>(23705.4 - 35659)	1287.8<br>(1028.7 - 1586.4)	22.8<br>(18.6 - 27.9)	1<br>(0.8 - 1.3)	8931.2<br>(6591.9 - 11535.7)	442.2<br>(302.6 - 611.1)	6.6<br>(4.9 - 8.6)	0.3<br>(0.2 - 0.4)
Japan	2117245.3<br>(1947749.6 - 2283110.5)	133915.2<br>(123133.9 - 144860.6)	17.6<br>(16.1 - 19)	1<br>(0.9 - 1)	373176.3<br>(308593.4 - 433966)	32277.4<br>(26541.6 - 37497.1)	2.6<br>(2.2 - 3)	0.1<br>(0.1 - 0.2)
Jordan	56931.1<br>(45697.5 - 70567.4)	1835.1<br>(1469.3 - 2280)	26.5<br>(21.3 - 32.7)	1.1<br>(0.9 - 1.4)	8257.2<br>(5859.6 - 10977.2)	258.6<br>(166.5 - 363.9)	3.9<br>(2.7 - 5.3)	0.2<br>(0.1 - 0.2)
Kazakhstan	596421.9<br>(523003.3 - 675959.1)	20858.6<br>(18305.9 - 23629.2)	85.7<br>(75.2 - 97.2)	3.5<br>(3.1 - 4)	77207.8<br>(60652.9 - 95555.3)	2787<br>(2117.2 - 3577.7)	8.4<br>(6.5 - 10.4)	0.3<br>(0.2 - 0.4)
Kenya	182463.4<br>(137432.7 - 237868.7)	5696.1<br>(4267 - 7439.9)	17.4<br>(13.2 - 22.5)	0.7<br>(0.5 - 0.9)	17573.2<br>(12692 - 24516.4)	797.3<br>(605.1 - 1048.1)	2<br>(1.5 - 2.6)	0.1<br>(0.1 - 0.1)
Kiribati	4034.1<br>(3108.8 - 5057.8)	122.6<br>(95.9 - 153.1)	120.2<br>(94.9 - 148.8)	4.6<br>(3.7 - 5.6)	1574.8<br>(1186.9 - 2071.2)	56.5<br>(42.8 - 73.6)	42.9<br>(32.7 - 55.5)	1.8<br>(1.4 - 2.4)
Kuwait	24151.6<br>(18540.7 - 30626.6)	611.9<br>(464.7 - 797.6)	15.6<br>(11.9 - 19.9)	0.6<br>(0.5 - 0.8)	2081.2<br>(1410.1 - 2885.7)	56.2<br>(36.9 - 80.1)	2.6<br>(1.7 - 3.7)	0.1<br>(0.1 - 0.2)
Kyrgyzstan	121145.9<br>(106385.8 - 137778.4)	4134.2<br>(3614.1 - 4708)	65.4<br>(57.5 - 73.9)	2.6<br>(2.3 - 3)	11531.1<br>(8473.1 - 15050.5)	399<br>(291.5 - 531.5)	5.2<br>(3.9 - 6.9)	0.2<br>(0.1 - 0.3)
Laos	117420.3<br>(90370.5 - 146755.4)	4111.8<br>(3171.7 - 5071.5)	68.2<br>(53.5 - 84.1)	3<br>(2.4 - 3.6)	40114.8<br>(26632.9 - 57824.5)	1439<br>(925.2 - 2129.1)	20.3<br>(13.5 - 29)	0.9<br>(0.5 - 1.3)
Latvia	72130.3<br>(65285.4 - 79229.6)	3021.6<br>(2720.6 - 3358.9)	54.2<br>(49.1 - 59.4)	2.3<br>(2.1 - 2.5)	14779<br>(12504.9 - 17220.4)	674.5<br>(537.3 - 850.6)	7.7<br>(6.6 - 8.8)	0.3<br>(0.2 - 0.3)
Lebanon	72949.3<br>(45228.1 - 106272.3)	2924<br>(1713.8 - 4371.5)	27.4<br>(17.1 - 39.7)	1.2<br>(0.7 - 1.8)	41262.4<br>(25900.1 - 57853.1)	1830<br>(1138.1 - 2575.5)	16.8<br>(10.6 - 23.6)	0.8<br>(0.5 - 1.1)
Lesotho	57139.8<br>(20651.3 - 106448.6)	1938.6<br>(746.8 - 3415.7)	124.2<br>(46.5 - 225.3)	4.8<br>(2 - 8.1)	9700.3<br>(1875.3 - 25815.5)	395.7<br>(68.5 - 1032.4)	16.5<br>(3.1 - 43.7)	0.7<br>(0.1 - 1.8)
Liberia	18978.2<br>(9983.7 - 36035.7)	630.6<br>(321.1 - 1213.7)	18.4<br>(9.8 - 34.1)	0.8<br>(0.4 - 1.4)	4343.8<br>(1173.6 - 10826.8)	161<br>(38.5 - 390.8)	4.1<br>(1.1 - 9.8)	0.2<br>(0 - 0.4)
Libya	78366.4<br>(60007 - 99131.2)	2716.2<br>(2099.3 - 3491.4)	36.8<br>(28.4 - 46.6)	1.6<br>(1.2 - 2.1)	3590.1<br>(1515.2 - 5911.8)	112<br>(47.7 - 188.9)	1.6<br>(0.7 - 2.6)	0.1<br>(0 - 0.1)
Lithuania	99234.9<br>(90109.1 - 108809.6)	4066.1<br>(3681.1 - 4469.6)	52.8<br>(47.9 - 57.8)	2.2<br>(2 - 2.4)	15158.8<br>(12796.2 - 17772.4)	589.2<br>(465.7 - 733.3)	5.9<br>(5 - 6.8)	0.2<br>(0.2 - 0.2)
Luxembourg	7869.4<br>(7169 - 8555.7)	383.7<br>(347.4 - 416.9)	22.2<br>(20.2 - 24.1)	1.1<br>(1 - 1.2)	3278<br>(2868.1 - 3668)	191.3<br>(158.4 - 223.6)	8.2<br>(7.2 - 9.2)	0.4<br>(0.3 - 0.4)
Macedonia	59531.3<br>(53602.6 - 65409.1)	2302.7<br>(2058.4 - 2567.5)	46.3<br>(41.6 - 50.9)	1.9<br>(1.7 - 2.1)	19290<br>(16422.7 - 22354.2)	774.3<br>(630.3 - 946.1)	13.7<br>(11.7 - 15.8)	0.5<br>(0.4 - 0.7)
Madagascar	220907.3<br>(81685.3 - 462194.8)	6572.2<br>(2435.9 - 13736.2)	35.8<br>(13.9 - 72.5)	1.3<br>(0.5 - 2.6)	23645.5<br>(8381.8 - 62207.9)	767.6<br>(273.7 - 1940.4)	3.8<br>(1.5 - 9.7)	0.1<br>(0.1 - 0.3)

Country or Territory	Male Smoking Attributable DALYs 2015 (95% UI)	Male Smoking Attributable Deaths 2015 (95% UI)	Male Smoking Attributable DALY Rate 2015, per 100 (95% UI)	Male Smoking Attributable Death Rate 2015, per 100 (95% UI)	Female Smoking Attributable DALYs 2015 (95% UI)	Female Smoking Attributable Deaths 2015 (95% UI)	Female Smoking Attributable DALY Rate 2015, per 100 (95% UI)	Female Smoking Attributable Death Rate 2015, per 100 (95% UI)
Malawi	83949.1 (39601.6 - 176250.8)	2930.6 (1340.4 - 6182.9)	24.8 (11.9 - 52.4)	1 (0.5 - 2.1)	12967 (5943.8 - 28579.3)	588.4 (263.2 - 1299.4)	3.7 (1.7 - 8)	0.2 (0.1 - 0.4)
Malaysia	538144.7 (439569 - 655940.8)	20324.3 (16650.9 - 24718.1)	45.6 (37.7 - 55.3)	2.1 (1.7 - 2.5)	72127.9 (55144.6 - 90801.4)	3126.8 (2354.8 - 4119.9)	6.8 (5.3 - 8.6)	0.4 (0.3 - 0.5)
Maldives	1719.0 (1309.8 - 2187.6)	64.4 (46.5 - 87.3)	14.7 (11.1 - 18.9)	0.6 (0.5 - 0.9)	317.2 (226.8 - 428.3)	11.5 (7.8 - 17.4)	2.8 (2 - 3.8)	0.1 (0.1 - 0.2)
Mali	47457.6 (22331.4 - 106056.9)	1444.1 (652.8 - 3279.6)	13.5 (6.4 - 29.8)	0.5 (0.2 - 1.1)	10441.1 (2483.5 - 34367.7)	369.1 (81.3 - 1221.1)	2.9 (0.7 - 9.4)	0.1 (0 - 0.4)
Malta	6688.5 (5994.8 - 7375.7)	333.4 (299.7 - 366.5)	21.1 (19 - 23.3)	1.1 (0.9 - 1.2)	1379.8 (1128.9 - 1644.9)	67.8 (51.1 - 86.5)	4.1 (3.4 - 4.8)	0.2 (0.1 - 0.2)
Marshall Islands	1374.2 (1007.8 - 1795.4)	38.6 (28.4 - 50.4)	56.8 (42.4 - 73.5)	2 (1.5 - 2.5)	309.3 (206.2 - 439.7)	10.1 (6.8 - 14.3)	13.6 (9.2 - 18.9)	0.5 (0.4 - 0.8)
Mauritania	17063.9 (6488.2 - 34842.2)	511.3 (166.4 - 1053.4)	15.9 (5.9 - 31.4)	0.6 (0.2 - 1.2)	4792 (1287.2 - 12116.4)	165 (36 - 423.7)	4.4 (1.1 - 10.9)	0.2 (0 - 0.5)
Mauritius	22800.9 (19099.6 - 26605.7)	849.1 (722.6 - 985.6)	34.2 (28.8 - 39.8)	1.5 (1.3 - 1.7)	2408.6 (1676.6 - 3211.5)	96.8 (59.7 - 136.9)	3.2 (2.2 - 4.2)	0.1 (0.1 - 0.2)
Mexico	618476.8 (529328 - 705138.9)	28153.8 (24727.3 - 31386.1)	13.6 (11.7 - 15.4)	0.7 (0.6 - 0.8)	189667.6 (155782 - 220906.8)	10291.9 (8092 - 12368.1)	3.7 (3.1 - 4.3)	0.2 (0.2 - 0.3)
Moldova	106011.4 (95199.3 - 116008.2)	3892.1 (3461.1 - 4299)	48.3 (43.3 - 53.1)	1.9 (1.7 - 2.1)	9859.7 (7798.4 - 12233.1)	337.9 (256.9 - 424.5)	3.5 (2.8 - 4.3)	0.1 (0.1 - 0.1)
Mongolia	88578.9 (77302.8 - 100599.6)	2960.1 (2583 - 3351.1)	92.3 (81.1 - 104.4)	3.9 (3.4 - 4.4)	15261.2 (12423.3 - 18385.8)	602.4 (489.6 - 738.2)	15 (12.3 - 18.1)	0.7 (0.6 - 0.9)
Montenegro	19435.1 (17199.2 - 21745.4)	808.8 (708.1 - 911)	49.6 (44 - 55.4)	2.1 (1.8 - 2.4)	8147.4 (6962.1 - 9542.8)	361.5 (292.7 - 448.2)	18.3 (15.7 - 21.3)	0.8 (0.6 - 0.9)
Morocco	357418.1 (222432.7 - 549236.6)	12359.7 (7285.9 - 19968.4)	25.5 (16 - 39)	1 (0.6 - 1.6)	55833 (22026.4 - 100783.5)	2071.4 (801.2 - 3681.9)	3.7 (1.5 - 6.6)	0.2 (0.1 - 0.3)
Mozambique	245573.3 (86770.4 - 514699.9)	8210.7 (2779.2 - 16915.2)	42.8 (15.5 - 87.7)	1.7 (0.6 - 3.2)	51063.8 (19803.9 - 132607.7)	1951.2 (693.8 - 5141.3)	7.6 (3 - 19.4)	0.3 (0.1 - 0.8)
Myanmar	1292920.3 (765776.3 - 1955094.3)	47619.2 (27638.2 - 70344)	68.8 (41.8 - 100.1)	3.1 (1.9 - 4.4)	419944.3 (223735.4 - 702325.6)	17658 (9207.9 - 29431.1)	19.9 (10.8 - 32.7)	1 (0.5 - 1.6)
Namibia	17876.8 (8130.1 - 36533)	621.8 (280.7 - 1249.3)	32.1 (15.1 - 63.3)	1.4 (0.7 - 2.6)	6288.9 (3519.1 - 14037.5)	285.4 (155.9 - 602.6)	10 (5.6 - 21)	0.5 (0.3 - 1.1)
Nepal	315462 (202328.1 - 461320.4)	12000.8 (7757.1 - 17462.7)	34.4 (22.4 - 49.6)	1.5 (1 - 2.2)	202915 (119203.5 - 333597.2)	8549.6 (4770.7 - 14353.1)	20.9 (12.2 - 34.2)	1 (0.6 - 1.7)
Netherlands	336994.6 (310274.6 - 364049.4)	18226.2 (16739.3 - 19679.1)	26.4 (24.3 - 28.5)	1.4 (1.3 - 1.6)	206659.3 (189714.1 - 224153.1)	11861 (10685.1 - 13098.7)	14.8 (13.6 - 16)	0.7 (0.7 - 0.8)
New Zealand	45876.6 (41424.1 - 50614.5)	2483.7 (2231.6 - 2736)	15.9 (14.3 - 17.5)	0.8 (0.7 - 0.9)	35958.6 (32991.4 - 39212.5)	2041.7 (1853.1 - 2248.1)	11.2 (10.3 - 12.3)	0.6 (0.5 - 0.6)
Nicaragua	16125.6 (12007.7 - 20726.3)	615.3 (448.2 - 795.2)	8.5 (6.3 - 10.9)	0.4 (0.3 - 0.5)	6828 (4672.2 - 9404.3)	265.8 (164.1 - 406.9)	3 (2 - 4.2)	0.1 (0.1 - 0.2)
Niger	58424.3 (24013.8 - 135054)	1702.8 (635.2 - 4064.8)	12.2 (4.9 - 28)	0.4 (0.2 - 1)	7341.4 (1792.6 - 22936.9)	276.9 (59 - 894.5)	1.9 (0.5 - 6)	0.1 (0 - 0.3)
Nigeria	424405.4 (263517 - 906477.9)	13781.5 (8536.3 - 29389.5)	10.4 (6.6 - 21.3)	0.4 (0.3 - 0.9)	112242.4 (41536.8 - 324211.4)	4539 (1631.3 - 12859.9)	3.3 (1.2 - 8.8)	0.2 (0.1 - 0.5)
North Korea	880086.8 (633976.2 - 1188353.9)	32208.6 (23047.3 - 42797.4)	76.3 (55.9 - 99.9)	3.5 (2.6 - 4.5)	343090.7 (228294.4 - 494444.5)	17194.5 (11081.4 - 25070.5)	22.8 (15.2 - 33)	1.2 (0.8 - 1.8)
Northern Mariana Islands	1553.6 (1259.8 - 1886.5)	40.2 (33.2 - 49)	48.8 (41.7 - 57.2)	2.3 (2 - 2.6)	482.2 (375.3 - 600.5)	13.2 (10.5 - 16.4)	19.9 (16.4 - 23.9)	0.9 (0.8 - 1.1)
Norway	60426.2 (54797.1 - 66789.6)	3247.9 (2938.1 - 3594.5)	17.1 (15.5 - 18.9)	0.9 (0.8 - 1)	44016.5 (40085.6 - 48360.8)	2919.4 (2561.5 - 3312.9)	11 (10.1 - 12.1)	0.6 (0.5 - 0.7)
Oman	19579.9 (14148.2 - 25508.8)	498.6 (345.6 - 668.8)	11.4 (8 - 14.7)	0.4 (0.3 - 0.6)	1444.9 (895.8 - 2203.1)	42.7 (25 - 66.8)	1.7 (1 - 2.7)	0.1 (0 - 0.1)

Country or Territory	Male Smoking Attributable DALYs 2015 (95% UI)	Male Smoking Attributable Deaths 2015 (95% UI)	Male Smoking Attributable DALY Rate 2015, per 100 (95% UI)	Male Smoking Attributable Death Rate 2015, per 100 (95% UI)	Female Smoking Attributable DALYs 2015 (95% UI)	Female Smoking Attributable Deaths 2015 (95% UI)	Female Smoking Attributable DALY Rate 2015, per 100 (95% UI)	Female Smoking Attributable Death Rate 2015, per 100 (95% UI)
Pakistan	2999330.3 (2325953.8 - 3881463.3)	101955.5 (78295.9 - 131764.8)	50.1 (39 - 64.4)	1.9 (1.5 - 2.5)	647067.8 (397580.3 - 961277.4)	22000.6 (13356.5 - 33221.4)	11.4 (7.1 - 17)	0.4 (0.3 - 0.7)
Palestine	49575.3 (34435.2 - 69362.7)	1577 (1080 - 2187.4)	44.8 (31.4 - 61.4)	1.8 (1.3 - 2.4)	4932.1 (2983.3 - 7785)	148.2 (87.8 - 240.9)	4.2 (2.5 - 6.6)	0.2 (0.1 - 0.3)
Panama	13866.9 (10250.7 - 18019)	711.4 (534.1 - 926.4)	8.7 (6.5 - 11.2)	0.5 (0.4 - 0.6)	4578.1 (2784.1 - 6605.8)	283.8 (152.6 - 418.9)	2.6 (1.6 - 3.8)	0.2 (0.1 - 0.2)
Papua New Guinea	213877.2 (126971.4 - 345965.6)	6357.9 (3672.5 - 10137.8)	100.1 (60.2 - 154.5)	3.9 (2.4 - 5.9)	134868 (75862.5 - 227575.2)	4311.5 (2364.5 - 7351.4)	60.2 (34 - 101.1)	2.3 (1.2 - 3.8)
Paraguay	54176.2 (42769.8 - 68182.8)	2307.4 (1803.6 - 2885.9)	23.6 (18.7 - 29.6)	1.1 (0.9 - 1.4)	20224.7 (14398.8 - 27002.4)	974.1 (646.1 - 1386.7)	8.7 (6.1 - 11.6)	0.4 (0.3 - 0.6)
Peru	91182.4 (68790.5 - 118908.4)	3871.8 (2853.4 - 5170.7)	7.7 (5.8 - 10)	0.4 (0.3 - 0.5)	45468.6 (31073.3 - 61254.6)	2517.7 (1589.8 - 3457)	3.5 (2.4 - 4.7)	0.2 (0.1 - 0.3)
Philippines	2084914.6 (1820989.6 - 2367003.8)	70955.2 (61861.2 - 80987.5)	65.1 (56.9 - 73.8)	2.8 (2.4 - 3.2)	564413.4 (466925.3 - 673995.9)	23038.9 (18305 - 28632.6)	17 (14 - 20.5)	0.8 (0.6 - 1.1)
Poland	1184039.9 (1104374.4 - 1263770.3)	49459.8 (46325.6 - 52644.6)	47.2 (44.1 - 50.4)	2 (1.9 - 2.2)	391454.1 (355358.8 - 428930.5)	17315.3 (15199.9 - 19799.1)	12.5 (11.4 - 13.6)	0.5 (0.4 - 0.6)
Portugal	192728.3 (178994.2 - 206649.8)	9009 (8249.1 - 9766.1)	25.5 (23.7 - 27.4)	1.1 (1 - 1.2)	35892.9 (28376.5 - 42488.2)	1696.7 (972.9 - 2316.1)	4.2 (3.5 - 4.9)	0.1 (0.1 - 0.2)
Puerto Rico	30235.2 (25767.7 - 35181.6)	1539.2 (1313.3 - 1787.5)	14.4 (12.3 - 16.8)	0.7 (0.6 - 0.8)	14717.9 (12105.8 - 17566.1)	1093.3 (878.6 - 1342.1)	5.2 (4.2 - 6.2)	0.3 (0.3 - 0.4)
Qatar	7107.9 (4857.4 - 9849.4)	168.3 (105.1 - 251.5)	9.2 (6.1 - 13.3)	0.4 (0.3 - 0.6)	596 (265.8 - 1004.6)	13.4 (5.6 - 23.7)	1.6 (0.8 - 2.8)	0.1 (0 - 0.1)
Romania	659691.2 (602955.5 - 719945.8)	26231.4 (23825.2 - 28905.4)	49.5 (45.2 - 54)	1.9 (1.8 - 2.1)	158408.6 (137544.9 - 181821.6)	6530.2 (5422.1 - 7946.1)	10.2 (8.9 - 11.6)	0.4 (0.3 - 0.4)
Russia	6525610 (5964684.5 - 7088856)	249095.9 (227470.3 - 272119.3)	77.3 (70.6 - 83.9)	3.1 (2.8 - 3.4)	983974.8 (852629.5 - 1128219.1)	33482.6 (28361 - 39553.8)	8.6 (7.5 - 9.8)	0.3 (0.2 - 0.3)
Rwanda	48823.8 (23597.4 - 107571)	1613 (761.1 - 3593.7)	21.7 (10.6 - 47.2)	0.9 (0.4 - 1.9)	20127.4 (9430.3 - 44314.1)	793.5 (369.9 - 1704.7)	7.3 (3.6 - 15.4)	0.3 (0.2 - 0.7)
Saint Lucia	1540.5 (1247.8 - 1872.2)	60.1 (47.8 - 73.5)	17.6 (14.3 - 21.5)	0.7 (0.6 - 0.9)	330.5 (242.3 - 434.7)	16.8 (11.1 - 22.8)	3.4 (2.5 - 4.5)	0.2 (0.1 - 0.2)
Saint Vincent and the Grenadines	1136.3 (927.4 - 1369.7)	42.4 (34.4 - 51.7)	22.6 (18.4 - 27.3)	0.9 (0.8 - 1.2)	205.2 (153.4 - 261.1)	8.5 (6.1 - 11.1)	4 (3 - 5.1)	0.2 (0.1 - 0.2)
Samoa	2526.1 (1856.6 - 3442.5)	84.4 (61.8 - 115.3)	38.7 (28.7 - 51.9)	1.5 (1.1 - 2)	914.5 (608.2 - 1311.6)	33.3 (21.8 - 48.3)	13.5 (9 - 19.4)	0.5 (0.3 - 0.8)
Sao Tome and Principe	1096.8 (341.6 - 2314)	46.5 (15.6 - 91.6)	30.3 (10.2 - 61)	1.5 (0.5 - 2.8)	468.1 (63.6 - 1198.9)	20 (2.5 - 49.7)	10.9 (1.5 - 27)	0.5 (0.1 - 1.2)
Saudi Arabia	155050.9 (124064.1 - 187999.4)	4307.2 (3422.2 - 5296.4)	12.2 (9.9 - 14.8)	0.5 (0.4 - 0.6)	12090.6 (9132.4 - 15632.6)	432.1 (325 - 567.5)	1.6 (1.2 - 2.1)	0.1 (0.1 - 0.1)
Senegal	72018.6 (27286.4 - 190258.5)	2296 (857.5 - 5962.3)	22.6 (8.7 - 58.2)	0.9 (0.3 - 2.1)	17662 (2974 - 57540.7)	677.1 (102.7 - 2145.3)	5.1 (0.9 - 15.6)	0.2 (0 - 0.7)
Serbia	281211 (259115.1 - 303234.7)	11757.4 (10816.3 - 12721.2)	46.4 (42.7 - 49.9)	1.9 (1.8 - 2.1)	111039.8 (98883.8 - 125311.9)	4968.3 (4278.1 - 5806.2)	15.9 (14.2 - 17.7)	0.6 (0.6 - 0.7)
Seychelles	1781.8 (1448.5 - 2166)	63.2 (51.3 - 77)	39.7 (32.6 - 47.8)	1.7 (1.4 - 2)	239 (161.1 - 335.5)	10.5 (6.8 - 15.3)	5 (3.4 - 7)	0.2 (0.1 - 0.3)
Sierra Leone	57077.1 (25650.9 - 109585.4)	1747.2 (758.5 - 3373.4)	37.7 (17.5 - 70.7)	1.4 (0.7 - 2.7)	18300.8 (6917.1 - 39350.3)	592.6 (204.6 - 1275.5)	11.9 (4.5 - 25.1)	0.5 (0.2 - 1)
Singapore	39332.1 (34512.7 - 44637.4)	2101.2 (1846.3 - 2391.8)	17.5 (15.4 - 19.8)	1 (0.9 - 1.1)	10584.9 (8634.6 - 12492.9)	762.5 (599.6 - 931.6)	4 (3.3 - 4.7)	0.3 (0.2 - 0.3)
Slovakia	130554.1 (118922.2 - 142593.4)	5257.3 (4766 - 5748.4)	38.5 (35 - 41.9)	1.7 (1.5 - 1.8)	29231.3 (24821.3 - 33694.6)	1183.2 (968.7 - 1413.5)	7 (6 - 8.1)	0.3 (0.2 - 0.3)
Slovenia	36090.2 (32507.7 - 39897.1)	1691.5 (1521.8 - 1879.1)	23.2 (20.9 - 25.6)	1.1 (1 - 1.2)	11032.1 (9306.8 - 12990.1)	611.3 (483.2 - 768.8)	6.2 (5.2 - 7.3)	0.3 (0.2 - 0.3)

Country or Territory	Male Smoking Attributable DALYs 2015 (95% UI)	Male Smoking Attributable Deaths 2015 (95% UI)	Male Smoking Attributable DALY Rate 2015, per 100 (95% UI)	Male Smoking Attributable Death Rate 2015, per 100 (95% UI)	Female Smoking Attributable DALYs 2015 (95% UI)	Female Smoking Attributable Deaths 2015 (95% UI)	Female Smoking Attributable DALY Rate 2015, per 100 (95% UI)	Female Smoking Attributable Death Rate 2015, per 100 (95% UI)
Solomon Islands	13339 (7729.7 - 21423.4)	413.6 (244.6 - 656.3)	81 (49 - 126.6)	3 (2 - 4.7)	5741.5 (2931.9 - 10085)	181 (89.7 - 314)	35.6 (18.3 - 61)	1.4 (0.7 - 2.3)
Somalia	83031.3 (23769 - 240900.3)	2574.9 (721.9 - 7135.1)	34.8 (10.6 - 95.2)	1.3 (0.4 - 3.2)	16834.9 (4027.2 - 56540.2)	625.2 (153.4 - 1891.6)	7.2 (1.9 - 22.5)	0.3 (0.1 - 0.8)
South Africa	888779.3 (759187.1 - 1033806.1)	32080.3 (27363.8 - 37008.8)	52.5 (45.1 - 60.5)	2.2 (1.9 - 2.6)	350249.5 (287342.3 - 425409.9)	15362.9 (12551.4 - 18759.3)	16.5 (13.5 - 20)	0.8 (0.6 - 0.9)
South Korea	711734.8 (627420.6 - 800157.2)	34574.6 (30615.8 - 38560)	23.6 (20.9 - 26.4)	1.3 (1.1 - 1.4)	126413.1 (100243.4 - 153392.2)	8808.3 (6871.5 - 10911.6)	3.3 (2.6 - 4)	0.2 (0.2 - 0.3)
South Sudan	87261.6 (27489.5 - 287996.6)	2776.7 (841.5 - 8987.1)	28.5 (9.4 - 91.3)	1.1 (0.4 - 3.2)	19315.1 (4286 - 78225.6)	720.6 (164.1 - 2644.4)	6.4 (1.6 - 24.5)	0.3 (0.1 - 0.9)
Spain	864544.6 (811884.8 - 921016.1)	46569.7 (42980.8 - 50205.4)	25.5 (23.9 - 27.2)	1.3 (1.2 - 1.4)	158848.6 (132217.4 - 186128.9)	6608.3 (4078.4 - 9707.9)	4.5 (3.9 - 5.1)	0.1 (0.1 - 0.2)
Sri Lanka	212411.3 (144311.9 - 295003.8)	7582.1 (5072.5 - 10731)	21 (14.4 - 29.1)	0.8 (0.6 - 1.2)	10741.3 (5077.5 - 22410.8)	425.2 (195 - 850.9)	0.9 (0.4 - 1.9)	0 (0 - 0.1)
Sudan	99636.7 (48966.8 - 182443.3)	3534.6 (1720 - 6438.3)	10.5 (5.3 - 18.8)	0.5 (0.2 - 0.8)	32677.1 (2367.1 - 79099.5)	994.5 (64.9 - 2479.9)	2.9 (0.2 - 7.2)	0.1 (0 - 0.3)
Suriname	6945.7 (5524 - 8374.1)	247.6 (192.4 - 305.1)	30.3 (24 - 36.7)	1.3 (1 - 1.6)	2231.7 (1670.4 - 2955.4)	91.7 (66.6 - 127.1)	8.9 (6.8 - 11.8)	0.4 (0.3 - 0.6)
Swaziland	18879.8 (4703 - 39706.8)	668.2 (167.6 - 1350.6)	70 (18.1 - 142.2)	2.9 (0.8 - 5.5)	5975 (919.1 - 17610.9)	242.8 (43.2 - 668.8)	18.9 (3.2 - 53.2)	0.9 (0.2 - 2.2)
Sweden	90735.1 (82001.7 - 100261.1)	5071.4 (4587.2 - 5627.2)	11.7 (10.6 - 13)	0.6 (0.6 - 0.7)	86262.6 (78974.8 - 93507.7)	5384.6 (4876.1 - 5915.5)	10 (9.1 - 10.9)	0.5 (0.5 - 0.6)
Switzerland	104519.4 (94834.2 - 115423.5)	5622 (5033.1 - 6266.4)	17.3 (15.7 - 19.1)	0.9 (0.8 - 1)	47446.1 (42172.9 - 52678.6)	2796.3 (2369.8 - 3271.4)	7 (6.2 - 7.7)	0.3 (0.3 - 0.4)
Syria	152435.5 (113387 - 191215.8)	5236.5 (3803.9 - 6658.9)	28.6 (21.1 - 35.9)	1.2 (0.8 - 1.5)	56119.6 (37544.4 - 77240.8)	2096.8 (1355.6 - 2934.5)	10 (6.7 - 13.8)	0.4 (0.3 - 0.6)
Taiwan	466821.7 (362955.8 - 581950.8)	22732.9 (17720.6 - 28013.6)	30.8 (23.9 - 38.4)	1.5 (1.2 - 1.8)	122742.2 (90740.7 - 160887.9)	6468.6 (4664.6 - 8641)	7.6 (5.5 - 9.9)	0.4 (0.3 - 0.5)
Tajikistan	61704.6 (51324.9 - 74357.4)	1957.7 (1611.3 - 2386.3)	25.3 (20.9 - 30.6)	1 (0.8 - 1.2)	4407.5 (2116.3 - 7217.3)	124 (61.4 - 204.6)	1.7 (0.8 - 2.8)	0.1 (0 - 0.1)
Tanzania	274874.9 (131129.9 - 626488.3)	9154.4 (4281.8 - 20598.2)	23.5 (11.5 - 52.8)	0.9 (0.4 - 2)	40882.3 (13964.4 - 125523.5)	1607.6 (547.5 - 4771.5)	3.7 (1.3 - 10.8)	0.2 (0.1 - 0.5)
Thailand	1895502.5 (1421336.1 - 2362788)	81399.6 (60408.7 - 98011.3)	49.4 (37.5 - 60.6)	2.4 (1.8 - 2.8)	382285.8 (286869.7 - 491831.3)	19069.6 (14334.6 - 24379.7)	8.9 (6.7 - 11.4)	0.5 (0.4 - 0.6)
The Bahamas	3212.3 (2525.3 - 3961)	115.1 (93 - 141.2)	17.3 (14 - 21.1)	0.7 (0.6 - 0.9)	1027.8 (744.9 - 1349.9)	42.1 (30.1 - 56.9)	4.8 (3.5 - 6.3)	0.2 (0.1 - 0.3)
The Gambia	10621.1 (5941.8 - 19615.1)	332.2 (182.6 - 617.8)	25.7 (14.7 - 46.3)	1 (0.6 - 1.9)	899 (245 - 2267.5)	34.8 (8.4 - 90.2)	2.7 (0.7 - 6.8)	0.1 (0 - 0.3)
Timor-Leste	9817.3 (6520.7 - 14097.4)	340.4 (218.3 - 496.2)	29.8 (20 - 42.3)	1.2 (0.8 - 1.7)	4579.2 (2790.2 - 7102.3)	180.4 (107.4 - 290.2)	13.8 (8.4 - 21.1)	0.6 (0.4 - 1)
Togo	50449 (18836.7 - 97758.1)	1678.6 (622.1 - 3206.7)	32.7 (12.8 - 61.3)	1.3 (0.5 - 2.4)	8559.3 (2302.6 - 22794.3)	329.6 (80.9 - 874.6)	5.4 (1.5 - 14)	0.2 (0.1 - 0.6)
Tonga	2199.5 (1667.9 - 2901.5)	85.8 (66.2 - 108.9)	68.4 (52.4 - 88.4)	3 (2.3 - 3.7)	597.3 (439.3 - 793.5)	30.6 (21.9 - 39.9)	15.4 (11.3 - 20.3)	0.8 (0.6 - 1)
Trinidad and Tobago	22503.6 (17849.4 - 27068.8)	816.1 (648.3 - 994.2)	31.5 (25.1 - 37.9)	1.3 (1 - 1.6)	2909.4 (2160.1 - 3840.6)	98.9 (68.1 - 136.2)	3.7 (2.7 - 4.8)	0.1 (0.1 - 0.2)
Tunisia	209837.6 (153443.7 - 279340.9)	8490.8 (6044.4 - 11480.9)	42.2 (30.9 - 56.1)	1.9 (1.4 - 2.6)	20120 (10686 - 32353.4)	1053.1 (505.1 - 1742.6)	3.8 (2 - 6.1)	0.2 (0.1 - 0.4)
Turkey	1238143.1 (1118149.1 - 1369707.6)	47751.9 (43166.7 - 53354)	38.1 (34.5 - 42.1)	1.7 (1.5 - 1.9)	240818.6 (201083.1 - 282856.1)	8390.5 (6325.7 - 10618.5)	6.1 (5.1 - 7.2)	0.2 (0.2 - 0.3)
Turkmenistan	59722.1 (49848.9 - 69650.3)	1829.1 (1532.8 - 2153.7)	31.6 (26.4 - 37)	1.2 (0.9 - 1.4)	4631.2 (3019.2 - 6362.7)	136.7 (92.2 - 185.2)	2 (1.3 - 2.8)	0.1 (0 - 0.1)

Country or Territory	Male Smoking Attributable DALYs 2015 (95% UI)	Male Smoking Attributable Deaths 2015 (95% UI)	Male Smoking Attributable DALY Rate 2015, per 100 (95% UI)	Male Smoking Attributable Death Rate 2015, per 100 (95% UI)	Female Smoking Attributable DALYs 2015 (95% UI)	Female Smoking Attributable Deaths 2015 (95% UI)	Female Smoking Attributable DALY Rate 2015, per 100 (95% UI)	Female Smoking Attributable Death Rate 2015, per 100 (95% UI)
Uganda	164371.8 (51761.5 - 370578.6)	5001.4 (1487.6 - 11093.8)	22.7 (7.1 - 49.9)	0.8 (0.2 - 1.8)	53973.1 (11634.1 - 151282.9)	1898.1 (411.1 - 5263.2)	7.5 (1.7 - 20.5)	0.3 (0.1 - 0.8)
Ukraine	2071332.9 (1847358.1 - 2291530)	84003.9 (74205.7 - 94034.9)	73.8 (65.9 - 81.6)	3 (2.7 - 3.4)	261369.4 (211072.4 - 309769.7)	10153 (7913.1 - 12882.8)	6.9 (5.6 - 8.1)	0.2 (0.2 - 0.3)
United Arab Emirates	81172.7 (50942.2 - 123374.5)	2002.2 (1244 - 3062.6)	16.5 (11.2 - 23.9)	0.7 (0.5 - 1)	5039.7 (2309.2 - 8710)	129.6 (61.4 - 222.8)	3.9 (2.2 - 6.4)	0.2 (0.1 - 0.3)
United Kingdom	1061743.1 (996403.1 - 1138461.8)	60642.5 (57036.8 - 64702.4)	23.1 (21.6 - 24.8)	1.2 (1.2 - 1.3)	761888.9 (719594.3 - 806410.5)	52514 (49832.5 - 55434.6)	14.2 (13.3 - 15)	0.8 (0.8 - 0.8)
United States	5616730 (5251296 - 5975033.5)	262287.6 (246692.1 - 277053.7)	27.2 (25.5 - 29)	1.3 (1.2 - 1.3)	4007570.5 (3807703 - 4213899.5)	209741.8 (201028.8 - 219551)	17 (16.1 - 17.9)	0.8 (0.8 - 0.8)
Uruguay	79566.3 (74157.6 - 85456.4)	3908.3 (3634.5 - 4215.6)	42.7 (39.8 - 45.9)	2.1 (1.9 - 2.2)	25566.8 (22620.2 - 28495.2)	1457.2 (1247.4 - 1685.8)	10.7 (9.5 - 12)	0.5 (0.4 - 0.5)
Uzbekistan	334615.3 (282447.8 - 390872.5)	10867.8 (9075.2 - 12814)	32.5 (27.3 - 38.2)	1.2 (1 - 1.5)	65985.8 (47822.8 - 86248.6)	2314.4 (1612.2 - 3150.5)	5.6 (4 - 7.3)	0.2 (0.1 - 0.3)
Vanuatu	5685.9 (3576.5 - 8911.4)	186.9 (122.7 - 282.7)	69 (45.1 - 104)	2.8 (1.9 - 4)	1305.6 (663.6 - 2384.4)	45.7 (24.1 - 80.4)	16.4 (8.8 - 28.7)	0.7 (0.4 - 1.2)
Venezuela	277664.3 (219312 - 341340.8)	10732.6 (8512.2 - 13037.8)	23.7 (18.9 - 28.8)	1.1 (0.9 - 1.3)	135507.6 (104807.4 - 170189.5)	6469.2 (4932.6 - 8231.5)	10.5 (8.1 - 13.2)	0.6 (0.4 - 0.7)
Vietnam	1961614.8 (1279403.3 - 2786501.3)	79441.1 (53283.5 - 107920.7)	55.5 (37.6 - 75.4)	2.6 (1.8 - 3.4)	489263.1 (285319.9 - 689464.2)	30387.9 (19300.9 - 41284.5)	10.6 (6.1 - 15.1)	0.6 (0.4 - 0.8)
Virgin Islands, U.S.	1473.3 (1198.8 - 1761.5)	64.7 (53 - 77.3)	19.6 (16 - 23.4)	0.9 (0.7 - 1)	575.1 (471.9 - 700.9)	31.4 (26 - 38.4)	6.4 (5.2 - 7.8)	0.3 (0.3 - 0.4)
Yemen	244444.5 (129498.2 - 424792.5)	7799.7 (4082.6 - 13657.5)	40.8 (22.3 - 70.1)	1.6 (0.9 - 2.7)	137127.8 (61755.4 - 267606.9)	4388.1 (1932.2 - 8703.9)	21.1 (9.5 - 41.2)	0.8 (0.3 - 1.5)
Zambia	147954.4 (62210.1 - 254641.9)	4996.7 (2143.9 - 8386)	48.9 (21.2 - 82.1)	2 (0.9 - 3.2)	28822.1 (12347 - 60605.1)	1298.7 (565.6 - 2591.4)	9.7 (4.3 - 19.5)	0.5 (0.2 - 0.9)
Zimbabwe	161475.1 (69130.7 - 286960.5)	5959.2 (2653.9 - 10195)	54.9 (24.6 - 95)	2.4 (1.2 - 4)	28817 (9671.9 - 71444.2)	1561.4 (566.3 - 3593.6)	10.5 (3.7 - 25.2)	0.6 (0.2 - 1.4)
